# Ts66Yah, a mouse model of Down syndrome with improved construct and face validity

**DOI:** 10.1242/dmm.049721

**Published:** 2022-12-06

**Authors:** Arnaud Duchon, Maria del Mar Muñiz Moreno, Claire Chevalier, Valérie Nalesso, Philippe Andre, Marta Fructuoso-Castellar, Mary Mondino, Chrystelle Po, Vincent Noblet, Marie-Christine Birling, Marie-Claude Potier, Yann Herault

**Affiliations:** ^1^ Université de Strasbourg, CNRS, INSERM, Institut de Génétique et de Biologie Moléculaire et Cellulaire (IGBMC), Department of Translational Medicine and Neurogenetics, 1 rue Laurent Fries, 67404 Illkirch-Graffenstaden, France; ^2^ Université de Strasbourg, CNRS, INSERM, CELPHEDIA, PHENOMIN-Institut Clinique de la Souris (ICS), 1 rue Laurent Fries, 67404 Illkirch-Graffenstaden, France; ^3^ Paris Brain Institute ICM, Hôpital de la Pitié-Salpêtrière, 75013 Paris, France; ^4^ Institut National de la Santé et de la Recherche Médicale, U1127, Hôpital de la Pitié-Salpêtrière, 75013 Paris, France; ^5^ Centre National de la Recherche Scientifique, UMR 7225, Hôpital de la Pitié-Salpêtrière, 75013 Paris, France; ^6^ Sorbonne Université, Hôpital de la Pitié-Salpêtrière, 75013 Paris, France; ^7^ Université de Strasbourg, CNRS UMR 7357, ICube, FMTS, 67000 Strasbourg, France

**Keywords:** Behavior and cognition, Gene dosage, Gene expression, Mouse model

## Abstract

Down syndrome (DS) is caused by trisomy of human chromosome 21 (Hsa21). The understanding of genotype–phenotype relationships, the identification of driver genes and various proofs of concept for therapeutics have benefited from mouse models. The premier model, named Ts(17^16^)65Dn/J (Ts65Dn), displayed phenotypes related to human DS features. It carries an additional minichromosome with the *Mir155* to *Zbtb21* region of mouse chromosome 16, homologous to Hsa21, encompassing around 90 genes, fused to the centromeric part of mouse chromosome 17 from *Pisd-ps2/Scaf8* to *Pde10a*, containing 46 genes not related to Hsa21. Here, we report the investigation of a new model, Ts66Yah, generated by CRISPR/Cas9 without the genomic region unrelated to Hsa21 on the minichromosome. As expected, Ts66Yah replicated DS cognitive features. However, certain phenotypes related to increased activity, spatial learning and molecular signatures were changed, suggesting genetic interactions between the *Mir155-Zbtb21* and *Scaf8-Pde10a* intervals. Thus, Ts66Yah mice have stronger construct and face validity than Ts65Dn mice for mimicking consequences of DS genetic overdosage. Furthermore, this study is the first to demonstrate genetic interactions between triplicated regions homologous to Hsa21 and others unrelated to Hsa21.

This article has an associated First Person interview with the first author of the paper.

## Teaser

**Summary:** We report investigation of a new Down syndrome (DS) mouse model, Ts66Yah, with stronger construct and face validity for mimicking consequences of DS genetic overdosage than the premier (Ts65Dn) mouse model of DS.

## INTRODUCTION

Knowledge of the pathophysiology of Down syndrome (DS), commonly known as trisomy 21, has been acquired from mouse models. Animal research has allowed the identification of several major driver genes linked to the clinical features found in people with DS, such as *DYRK1A* and *CBS*, and made possible the pre-clinical validation of therapies with several drug candidates (Duchon and Herault, 2016; Herault et al., 2017; Nakano-Kobayashi et al., 2017; Faundez et al., 2018; Neumann et al., 2018; Nguyen et al., 2018; Marechal et al., 2019). Over the past decades, even more complex mouse models have been generated, carrying one human chromosome 21 (Hsa21; ‘Hsa’ for *Homo sapiens*) almost complete (O'Doherty et al., 2005; Kazuki et al., 2020) and various segmental duplications of regions homologous to Hsa21, making it possible to dissect genotype–phenotype relationships in DS (Herault et al., 2017; Duchon et al., 2021).

These studies were pioneered and strongly driven by the use of the Ts(17^16^)65Dn/J (Ts65Dn) mouse line (Davisson et al., 1990, 1993; Reeves et al., 1995), with more than 500 publications found in the PubMed database (November 2022). Originally, the Ts65Dn line was generated by the irradiation of DBA/2J males, which were then crossed with C57BL/6J females, with the progeny being checked for chromosomal abnormalities (Davisson et al., 1990, 1993). Translocations for mouse chromosome 16 (Mmu16; ‘Mmu’ for *Mus musculus*) homologous to Hsa21 were then mated with the C57BL/6J×C3H/HeJ F1 hybrid line. The genetic background of B6C3HF1 was selected to obtain large litters and maintain higher transmission. The translocated minichromosome, Ts65Dn, encompasses 90 protein-coding genes (PGCs) from *Mir155* to *Zbtb21*, linked to the centromeric part of mouse chromosome 17 (Mmu17), which contains a region non-homologous to Hsa21, from *Pisd-ps2* to *Pde10a*, with approximately 46 PGCs (Muñiz Moreno et al., 2020). The region *Pisd-ps2* to *Pde10a* is homologous to the region between *SCAF8* and *PDE10A* found on human chromosome 6. It encompasses many genes involved in neuronal function (*CEP43*, *NOX3*, *PDE10A*, *RNASET2*, *SERAC1*, *TULP4*) and genetic disorders [*ARID1B*, Coffin-Siris syndrome 1 (www.orpha.net; ORPHA:1465) or microdeletion syndrome 6q25.2-q25.3; (ORPHA:251056) (Nagamani et al., 2009); *SERAC1* deficiency and MEGDEL syndrome (ORPHA:352328); recessive mutations of *GTF2H5* and trichothiodystrophy (ORPHA:33364); *RSPH3* involved in primary ciliary dyskinesia (ORPHA:244); and *PDE10A* linked to infantile movement disorders (ORPHA:494541 and ORPHA:494526)].

Owing to trisomy of the *Pisd-ps2* to *Pde10a* region in the Ts65Dn, the genetic validity of the model has been a topic of debate for quite some time. On the one hand, the presence of a supernumerary freely segregating chromosome may contribute to producing additional phenotypes compared to models with intrachromosomic duplications (Goodliffe et al., 2016; Olmos-Serrano et al., 2016); but, on the other hand, the presence of 60 genes non-homologous to Hsa21 may have a phenotypic impact not related to DS and too often neglected in various studies. Somehow, the effect of this additional triplicated segment has been set aside, and its contribution to Ts65Dn phenotypes remains undetermined.

To solve this dilemma and have a model closer to DS, we developed a new line named Ts66Yah, derived from the Ts65Dn lineage but no longer carrying the duplicated centromeric part of Mmu17. Here, we report its first phenotypic and molecular characterization.

## RESULTS

### Structure of the *Scaf8-Pde10a* proximal region of Mmu17

Before creating the Ts66Yah model, we looked at the *Scaf8-Pde10a* region. We found it quite rearranged in several mouse lines, according to the Mouse Genome Informatics database. In particular, we closely investigated the corresponding segments in the DBA/2J line used to generate the first Ts65Dn chromosome, and also in the C57BL/6J and C3H/HeJ (as a proxy) mouse lines used to breed the Ts65Dn mouse carriers. Overall, the region was globally conserved in its organization but several loci were affected (Fig. S1A). More precisely, the *Snx9* locus was different in size in the three models and more perturbed in C3H/HeJ. Remarkably, the genetic interval encompassing *Tulp4*, *Tmem181a* and *Sytl3* was not found in the DBA/2J genome, and other loci for *Rpsh3* and *Rnaset2* were duplicated differently in the three genetic backgrounds (Fig. S1B). Interestingly, the region is well preserved in humans on chromosome 6, with an organization similar to that of C57BL/6J, except for large inversions near the *Pde10a* and *Rps6ka2-Rnaset2* genes that occurred during evolution.

### Creation, validation and transmission of the new Ts66Yah minichromosome

**Fig. 1.**
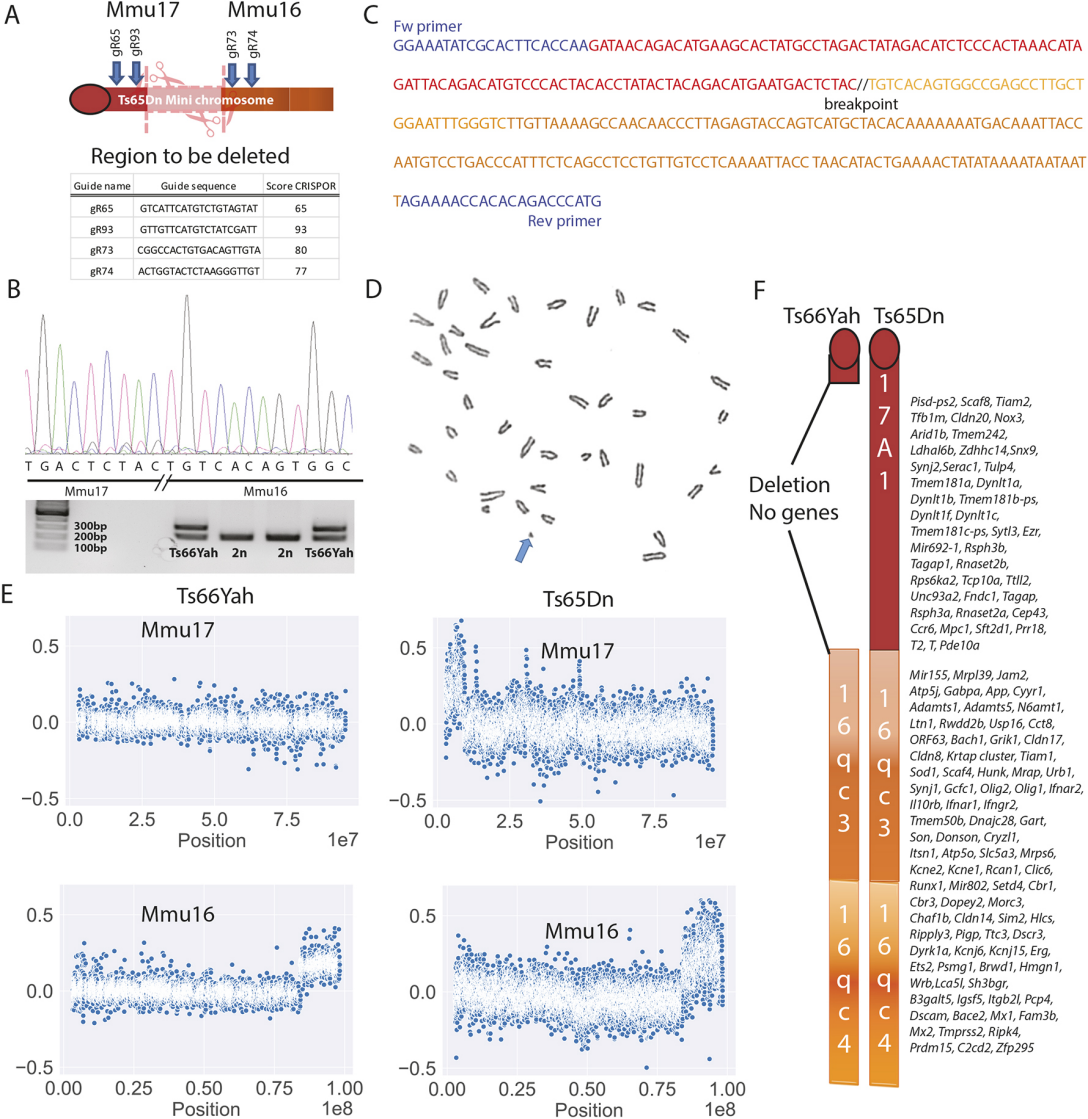
**Generation and validation of the new Ts66Yah mouse model.** (A) Representation of the deletion produced in Ts65Dn using CRISPR/Cas9 and two pairs of gRNAs. (B) Sequence electropherogram and PCR amplification products from the genotyping of Ts66Yah mice. (C) Genomic sequence of the new junction found in the deleted minichromosome of Ts66Yah mice. Blue font shows PCR primer localizations. (D) One metaphase spread showing the presence of an additional minichromosome (arrow) in Ts66Yah fibroblasts. (E) Comparative genomic hybridization (log2) of genomic DNA from Ts66Yah mice versus wild type (180K probes) compared to Ts65Dn mice (two 100K probes). (F) Comparison of the Ts66Yah and Ts65Dn minichromosomes. Orange and red colors show the sequence of Mmu17 and Mmu16, respectively. Numbers with letters represent the Giemsa banding.

Thus, we decided to remove the centromeric segment of Mmu17 located on the Ts65Dn minichromosome using the CRISPR/Cas9 technique. Embryos obtained from *in vitro* fertilization, taking sperm from selected fertile males from the Ts65Dn ‘1924’ line (Shaw et al., 2020) and wild-type (wt) F1B6C3B oocytes, were injected with CRISPR/Cas9 and the pairs of selected gRNAs based on the CRISPOR score (Concordet and Haeussler, 2018) (Fig. 1A). One founder carrying the recombined minichromosome, with the deletion of the centromeric part of Mmu17, was selected in the progeny and crossed with C57BL/6NCrl females. Two offspring, one male and one female, were used to start the new colony. The extent of the deletion was characterized by Sanger sequencing of the PCR fragment encompassing the deleted region (Fig. 1B,C), leaving a piece of Mmu16 encompassing *Mir155* to the end of the telomeric Mmu16 (∼13,856,661 bp). We characterized the new breakpoint between the genomic base number 3,071,436 on Mmu17 and 84,354,894 on Mmu16 [UCSC Genome Browser on Mouse December 2011 (GRCm38/mm10) Assembly)].

We confirmed the presence of an independent chromosome on metaphase spreads in Ts66Yah embryonic fibroblasts isolated from 14.5 days post coitum mutant embryos (Fig. 1D). We also performed comparative genomic hybridization (CGH) to check the copy number of the Mmu16 and Mmu17 chromosomes in both the Ts66Yah and Ts65Dn models (Fig. 1E). We were able to confirm that the Ts66Yah model indeed lacks the increase in gene dosage for the telomeric part of the Mmu17 region from *Pisd-ps2* to *Pde10a* seen in the Ts65Dn model (Fig. 1E,F), whereas the copy number of the region located on Mmu16 homologous to Hsa21 increased. Moreover, we checked that the change in the Mmu16 copy number in the Ts66Yah mice was located upstream of *Mrpl39* (Duchon et al., 2011), as expected, between two probes targeting the Mmu16 genetic interval 84,325,686-84,356,080 from the mouse genome assembly GRCm38/mm10.

We also checked the genetic transmission of the minichromosome. After establishing the line, the transmission rate (Table 1) began to stabilize at ∼30% on both F1B6C3B and C57BL/6J (B6J) genetic backgrounds. We thus decided to maintain the mixed genetic background used for the Ts65Dn mice, with the sighted C3H (C3B) males crossed with B6J females, namely B6C3B, that we commonly use in our laboratory. Currently, we have a stable ratio of transmission from both male and female germlines (Table 1), although not all the Ts66Yah males are fertile. We hypothesized that the increased fertility of Ts66Yah was a consequence of the selection of fertile Ts65Dn males when generating the model. To test this, we performed a sperm analysis to evaluate the quality of the sperm by looking at different parameters, such as the concentration of spermatozoids, their motility, their velocity and their progressivity, for both Ts66Yah and Ts65Dn lines (Fig. S2). As expected, the Ts65Dn mice showed poorer performance than wt littermates for all the sperm parameters analyzed. Similar differences were also observed in the Ts66Yah male sperm, which were lower quality than the sperm of the wt littermates. Nonetheless, we were able to isolate 31 fertile individuals out of the 49 males tested for fertility in the Ts66Yah line, compared to three fertile individuals out of 39 males in Ts65Dn, which is below the ranges reported previously for Ts65Dn in two centers (Moore et al., 2010).

**Table 1.**
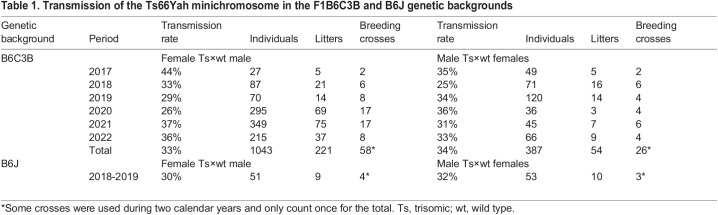
Transmission of the Ts66Yah minichromosome in the F1B6C3B and B6J genetic backgrounds

### Ts66Yah mice display lower locomotor activity than Ts65Dn mice in many tests

To study the behavioral phenotypes under the same environmental conditions, we produced separate cohorts for the two DS models from crosses of wt B6C3B males with Ts66Yah or Ts65Dn females. Then, we challenged the Ts66Yah, Ts65Dn and wt littermate animals to a battery of behavioral tests.

First, we assessed their adaptation to a novel environment and exploration activity in a brightly illuminated (120[Supplementary-material sup1]lux) square open field. The Ts66Yah mutant mice showed the same pattern of exploration as their wt littermates, with no significant differences between the two groups for either the distance traveled or the number of rears (Fig. S3A). In contrast, the Ts65Dn mice traveled further, notably more in the peripheral zone, and performed fewer rears in the central zone compared to their wt littermates. These results therefore indicate that only the Ts65Dn, but not the Ts66Yah, mutants presented an increase in anxiety-like behavior, as emphasized by reduced activity in the center of the arena and increased activity in the peripheral zone of the open field.

We then wondered whether the circadian activity of the mutant animals was altered, as reported previously for the Ts65Dn model (Ruby et al., 2010). We therefore tested the activity of the mice for 46 h (two nights). Ts66Yah and Ts65Dn mutants exhibited a similar pattern of circadian activity compared to their control littermates (Fig. S4A). However, we observed that the distance traveled by the Ts65Dn mutants was increased in the light and dark phases compared to that of the control mice, whereas the activity level of the Ts66Yah mice was comparable to that of the controls. This result was confirmed by the number of rears, which was also increased in the Ts65Dn line, even in the habituation phase (Fig. S3B). Similar increases in activity were observed in the number of Y-maze arm entries (Fig. S3C). Thus, the hyperactivity phenotype detected in the Ts65Dn mutants in several paradigms was absent in the Ts66Yah mice.

### Robust cognitive deficits in the Ts66Yah mouse models

Several learning and memory deficits have been described in the Ts65Dn mouse line by different teams, and are considered as robust and reproducible phenotypes (Reeves et al., 1995; Martinez-Cue et al., 2002; Fernandez et al., 2007; García-Cerro et al., 2017; Aziz et al., 2018; Duchon et al., 2021). Thus, we tested how much those robust phenotypes were present in the Ts66Yah individuals.

**Fig. 2.**
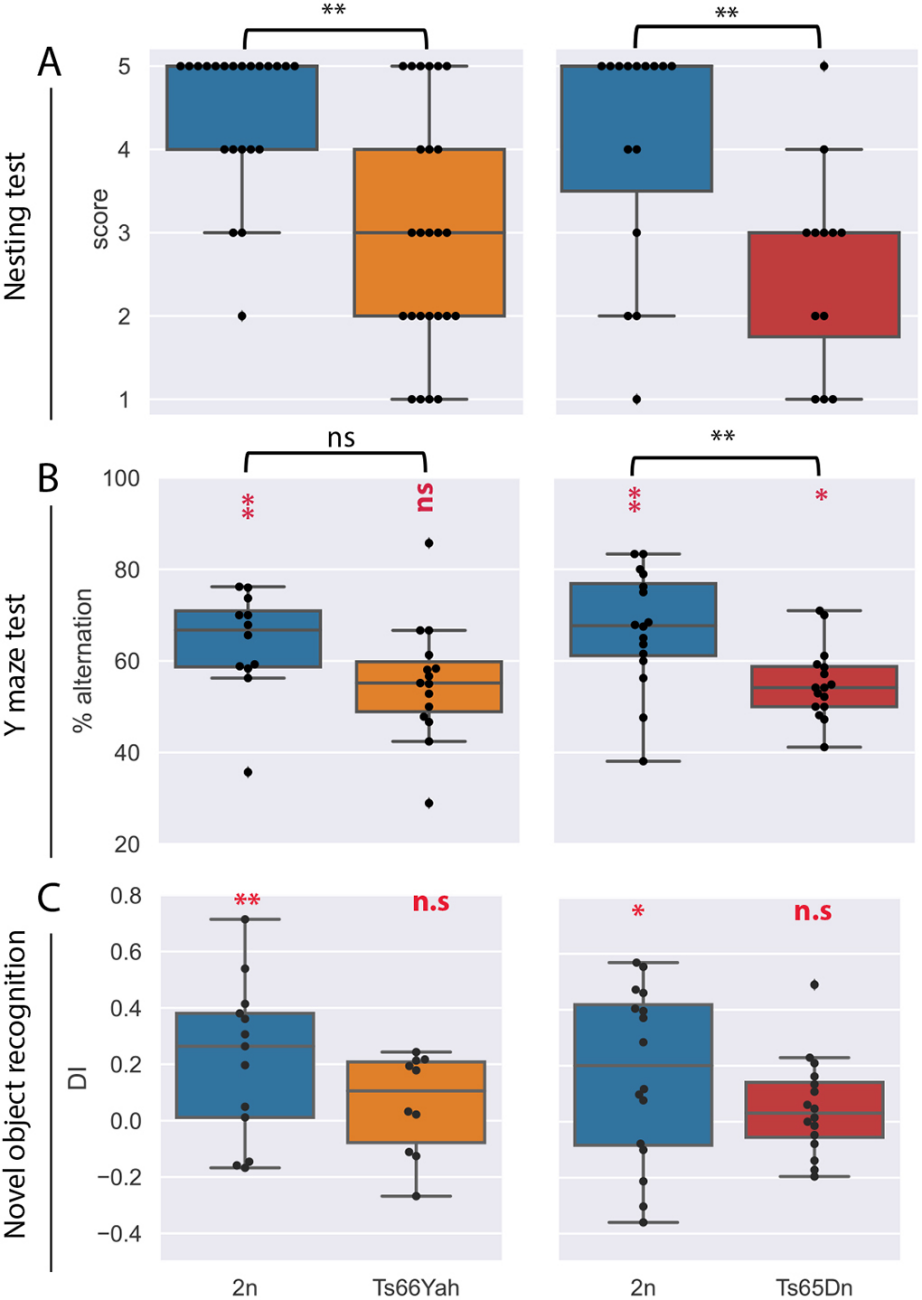
**Nesting activities and working memories display similar changes in male Ts66Yah and Ts65Dn mice.** (A) Mice with trisomy showed deficits in building a nest, whereas the majority of 2n mice were able to build a nest (22 2n males versus 25 Ts66Yah males, and 15 2n males versus 12 Ts65Dn males). (B) Although there was no significant difference in the percentage of alternation between 2n and Ts66Yah males, the level was significantly above 50% (chance level) in 2n, but not in Ts66Yah, mice. Conversely, the Ts65Dn line showed a strong difference in males, with a significantly lower percentage of alternation for the trisomic group compared to chance level, as well as reduced spontaneous alternation compared to 2n mice. (C) In the novel object recognition test, the discrimination index (DI) analysis indicated that Ts66Yah and Ts65Dn males were not able to distinguish the novel object (DI was close to 0). Box plots with the median and quartiles. Statistical significance of differences between genotype was inferred by an unpaired two-tailed *t*-test or Kruskal–Wallis non-parametric test, *P<0.05, **P<0.01. One-sample two-tailed *t*-test result versus 0 for DI in C, or versus 50 for Y maze in B, is indicated in red.

First, we started to analyze alterations linked to the normal innate behavior of rodents with the nest-building task, which is sensitive to HIP lesions and is an ancillary parameter assessed to predict cognitive defects (Heller et al., 2014). The organization of the nest was scored after one night, with a scale from 0, equivalent to the absence of a nest, to 5 when a full dome is raised. For both lines, most control disomic mice built a nest with a dome (score higher than 3; Fig. 2A); nevertheless, the nesting score was lower for both trisomic lines compared to that of the control littermates. Intriguingly, nine out of 25 Ts66Yah males built a nest with a score above 3, whereas only two out of 12 Ts65Dn males reached this stage. Further investigation would be needed to confirm this observation.

Then, we assessed spatial working memory by placing the mice in a Y maze and leaving them to freely explore the three arms. The total number of entries to any arm was scored as an index of locomotor activity. In the Ts66Yah mouse line, the percentage of alternation of the mutant mice was significantly different from chance level, but no strong difference was observed between trisomic and control individuals. By contrast, there was a strong difference for the Ts65Dn mutants, which showed a lower percentage of spontaneous alternation than their control littermates, with a percentage of alternation for the mutant group significantly different from 50% (Fig. 2B).

Afterwards, we evaluated episodic non-spatial recognition memory using the novel object recognition (NOR) paradigm, with a retention time of 24 h. During the acquisition session, there was a difference in the exploration time between both Ts65Dn and Ts66Yah mutant animals versus their respective control groups (Fig. S3D). Indeed, both DS model mice spent more time exploring the objects. During the test session, 24 h after the acquisition session, when one of the two familiar objects was replaced by a new one, both DS mice models explored both objects at the same rate and did not show any preference for the novel object, in contrast to the control mice (Fig. 2C). The Y-maze and object recognition phenotypes were reproduced in independent groups of male and female mice, bred and tested in a second laboratory. Both trisomic males and females showed the same defects in the Y-maze and NOR tests without any sex effect (Fig. S5).

**Fig. 3.**
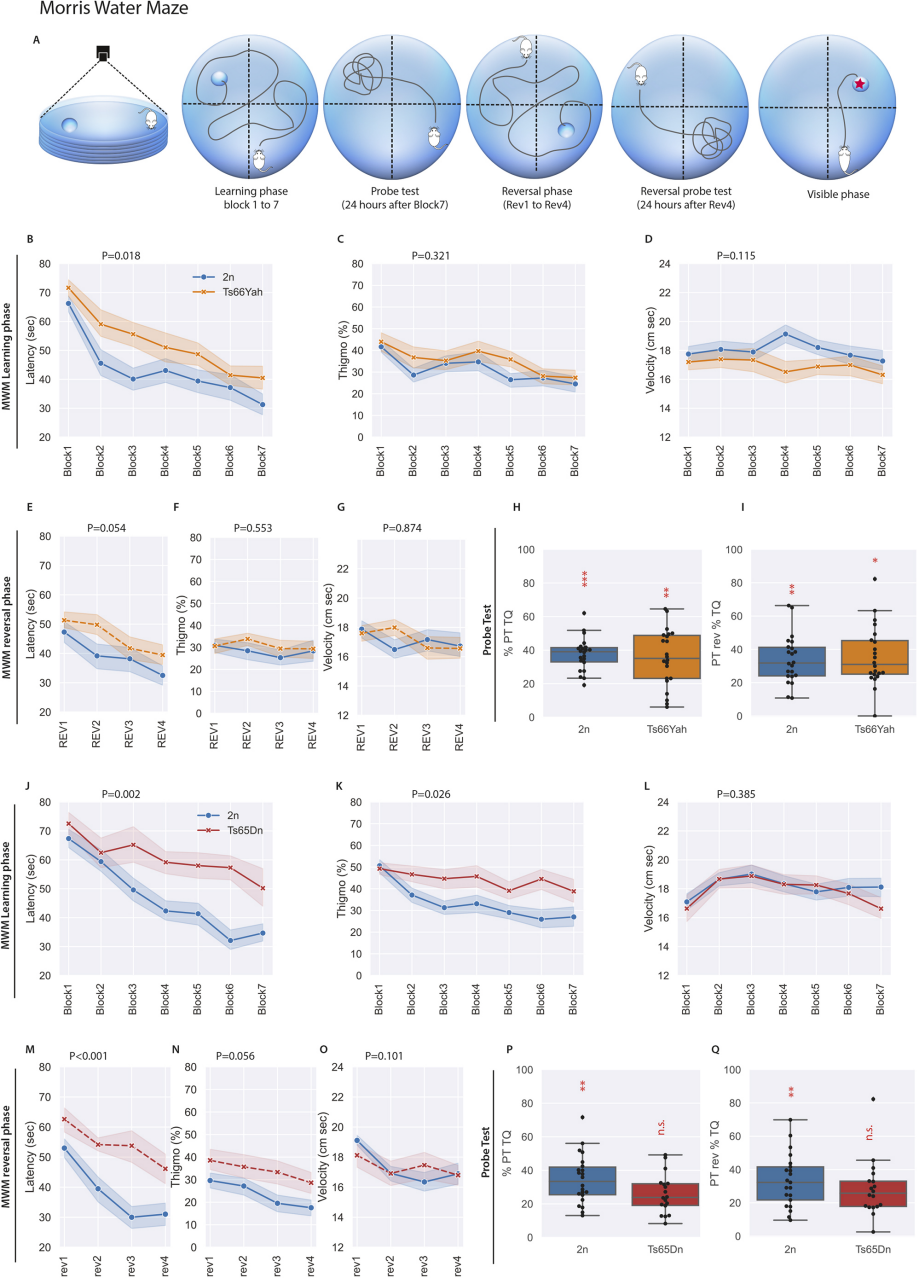
**Spatial learning and memory in the Morris water maze (MWM) task differs between male Ts66Yah and Ts65Dn mice.** (A) Schematic representation of the test. (B,J) Both trisomic mice (22 2n males versus 22 Ts66Yah males, B; and 20 2n males versus 18 Ts65Dn males, J) exhibited a delay in acquisition during the learning phase of the test, resulting in increased latency to find the platform. (C,D,K,L) In addition, the Ts65Dn mice presented increased thigmotaxic behavior (C,K), while velocity was stable regardless of the genotype (D,L). (H,P) In the probe test, only the Ts65Dn mice did not present increased exploration of the target quadrant, indicating a clear deficit in reference memory not observed in Ts66Yah mice. (E) During the reversal phase, the augmented latency to find the platform was close to the significant level for the Ts66Yah mice, whereas the difference was clearly increased for the Ts65Dn mice. (M) There was no difference in velocity (D,G,L,O) for both lines and the thigmotaxic behavior was not found in the Ts66Yah males compared to Ts65Dn (C,F versus K,N). (I,Q) For the reversal probe test, once again, only the Ts65Dn mice did not present preferences for the target quadrant. Box plots with the median and quartiles. Statistical significance of differences between groups was inferred by a repeated measures ANOVA. *P<0.05, **P<0.01, ***P<0.001. One-sample two-tailed *t*-test result versus 25% is indicated in red in the graph for PT. *P*-values for repeated measures ANOVA (genotype, block) are indicated for B-G and J-O. PT, probe test; TQ, target quadrant.

In the next step, we focused on the spatial reference memory in the Morris water maze (MWM) task. In this task, mice must learn how to escape from a circular pool of opaque water by localizing a hidden platform, set at a single fixed location, using distal spatial cues. Three different sessions were organized according to the diagram shown in Fig. 3A. First, a standard learning phase session in which a hidden platform is presented was performed, followed by a reversal session to detect memory flexibility. Both sessions ended with a probe test. Finally, a visible session was performed to assess the general capacity of the mice to perform the test and assess any visual or physical impairments. We measured the velocity, thigmotaxis and time spent by each individual to reach the platform. For the Ts66Yah line (Fig. 3B-I), there was no significant difference between the mutant and control group for thigmotaxis and swimming speed; however, the Ts66Yah mutants took longer to reach the platform during the sessions, indicating poorer performance than that of the control group. In addition, Ts66Yah mice learned the platform location in the last block of the trial with half the latency as in the first block of the trial. The same profile was observed in the reversal phase.

The retention memory of place location was assessed during a single probe trial with no platform available, 24 h after the last training session. In this probe test, all the mice remembered where the platform was located after the two learning sessions (learning and reversal phase). As expected, the Ts65Dn mutants (Fig. 3J-Q) presented an increased level of thigmotaxis compared to the controls and an increased latency, indicating that the mutants had difficulties in learning the platform location. This conclusion was supported by the analysis of the reversal-phase data. In addition, the Ts65Dn mutants did not present any preference for the target quadrant, with a level of time exploration close to 25% for both the learning and reversal phases.

Efficacy depends on swimming strategies to search for the platform. Thus, we analyzed the strategies to search for the platform undertaken by each individual along the test phase. Only the Ts65Dn mice showed a low and constant high level of non-spatial navigation; the level of spatial strategy increased in both the disomic (2n) mice and Ts66Yah animals during the initial learning and reversal-phase sessions, but not in Ts65Dn mice (Fig. S4C). In the visible session (Fig. S4C), the latency of Ts66Yah mice to reach the platform was higher that of the control mice, with a wider distribution. However, in both lines, the mean latency to reach the platform was statistically inferior (Fig. S4C) to the mean latency in the last session of the reversal phase when the platform was hidden (Fig. 3I,Q, *P*<0.001). This indicates that the mice did not have any deficit that could have prevented them from learning and passing the test. Overall, our results confirmed severely impaired spatial learning in Ts65Dn mice but not in the Ts66Yah mice.

Finally, both mutant lines were tested for contextual associative memory deficits in the Pavlovian fear conditioning (FC) test. To induce fear responses, the mice were placed in a ‘fear context’, a context box in which they were subjected to an electric shock. When the animals were re-subjected to the shock 24 h later in the same fear context, the level of freezing in all groups was increased compared to that in the habituation session, indicating that all groups developed a behavioral fear response during the training session (Fig. S4B). The Ts65Dn mutants presented a lower level of responses than the control group, especially at the end of the context session (the last 2 min of the session, named CONT3). By contrast, the Ts66Yah mutants did not show any difference in freezing time compared to that of the control group. Thus, we concluded that the hyperactivity of Ts65Dn mutants interfered with this test: the Ts65Dn mutants would not be subject to memory failure but instead could not stop moving during a session lasting 2 min.

### Identification of discriminating phenotypic variables between Ts66Yah and Ts65Dn

**Fig. 4.**
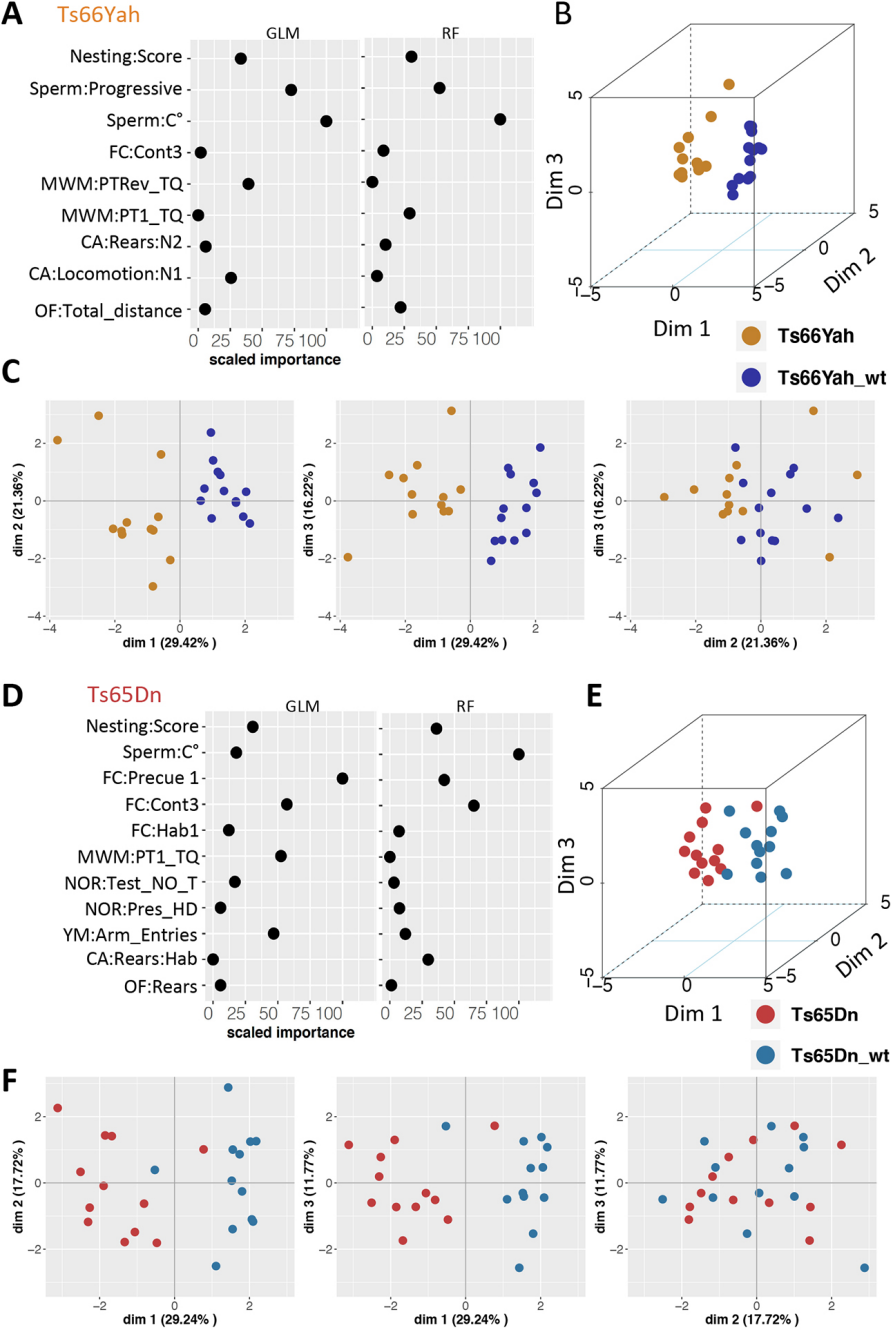
**Identification of the strongest phenotypic variables contributing to genotype discrimination in male Ts66Yah and Ts65Dn mice.** (A-D) Importance of each explanatory phenotypic variable in the genotype discrimination. The selected variables were those known to contribute more than 30% to the genotype discrimination. All measures of importance are scaled to obtain a maximum value of 100 for the variable contributing most to the discrimination in the comparison of Ts66Yah Down syndrome (DS) mutants versus wild types. (B,C) 3D principal component analysis (PCA) plots showing the individual animals clustering on the 3D space based on the PCA analyses performed with all the phenotypic variables and colored based on genotype and model as follows: dark blue, Ts66Yah wild type; yellow, Ts66Yah DS mutant. (C-F) Individual component map. The distribution in 2D space of the individual observation coordinates calculated based on the PCA analysis performed after the multiple factor analysis (MFA) of the MFAmix function. Ts66Yah mice are shown in A-C; Ts65Dn mice are shown in D-F.

Considering the differences observed in several phenotypic behavioral variables (Table S1), we wanted to assess the importance of each variable for the genotype-based classification using Gdaphen (see Materials and Methods). First, we found that several variables considered for the analysis (Table S2) were highly correlated (more than 86% correlation), and thus they were removed from the respective downstream analysis for both Ts65Dn and Ts66Yah with their respective wt controls (Table S3). The statistical classifiers GLM-Net and random forest (RF) identified several variables that discriminated the trisomic from the control individual in each model separately (Fig. 4). Using both classifiers, the three most important variables to discriminate Ts66Yah versus wt individuals were ‘sperm concentration’, ‘sperm:progressive’ and ‘percentage of time spent in the target quadrant in the first probe test’ (MWM:PTRev_TQ; GLM-NET; Table S4) with the ‘nesting score’ (RF; Fig. 4A; Table S4). In addition, the wt and trisomic individuals were easily separated in principal component analysis (PCA) (Fig. 4B), with 67% of the variance explained with the three first dimensions (Fig. 4C). To discriminate Ts65Dn versus wt individuals, the situation was different for both classifiers (Fig. 4D; Table S4). The most important variables were ‘FC:Precue1’ (GLM-Net) and ‘sperm concentration’ (RF), then ‘FC:CONT3’ (both), and in third position ‘MWM:PT1_TQ’ (GLM-Net) and ‘FC:Precue1’ (RF) (Fig. 4D; Table S4). Approximately 59% of the variance was explained with the three first dimensions of the PCA to separate Ts65Dn and wt individuals (Fig. 4F).

Then, we analyzed the discriminating variable combining all the genotypes (Table S5, Fig. S6). We found more behavioral variables to discriminate the wt and trisomic individuals. Aside from the first one, ‘sperm:progressive’, the next six variables were ‘FC:Hab2’, ‘MWM:PTRev_TQ’, ‘NOR:Test_NO’, ‘YM:Spont_Alter’, ‘CA:Rears:N2’ and ‘CA:Locomotion:Light’, although the percentage of variance explained was even lower in the three first dimensions, only 46%. The multiple factor analysis (MFA) computed the correlation between the qualitative or quantitative variables grouped by test or ungrouped, and the principal component dimensions. For both DS models, the MFA and the cosine diagrams highlighted almost the same variables as the main contributors to differentiate between Ts65Dn or Ts66Yah and their respective wts.

### Comparison of morphological alterations in the brain and skull of Ts66Yah and Ts65Dn mice

**Fig. 5.**
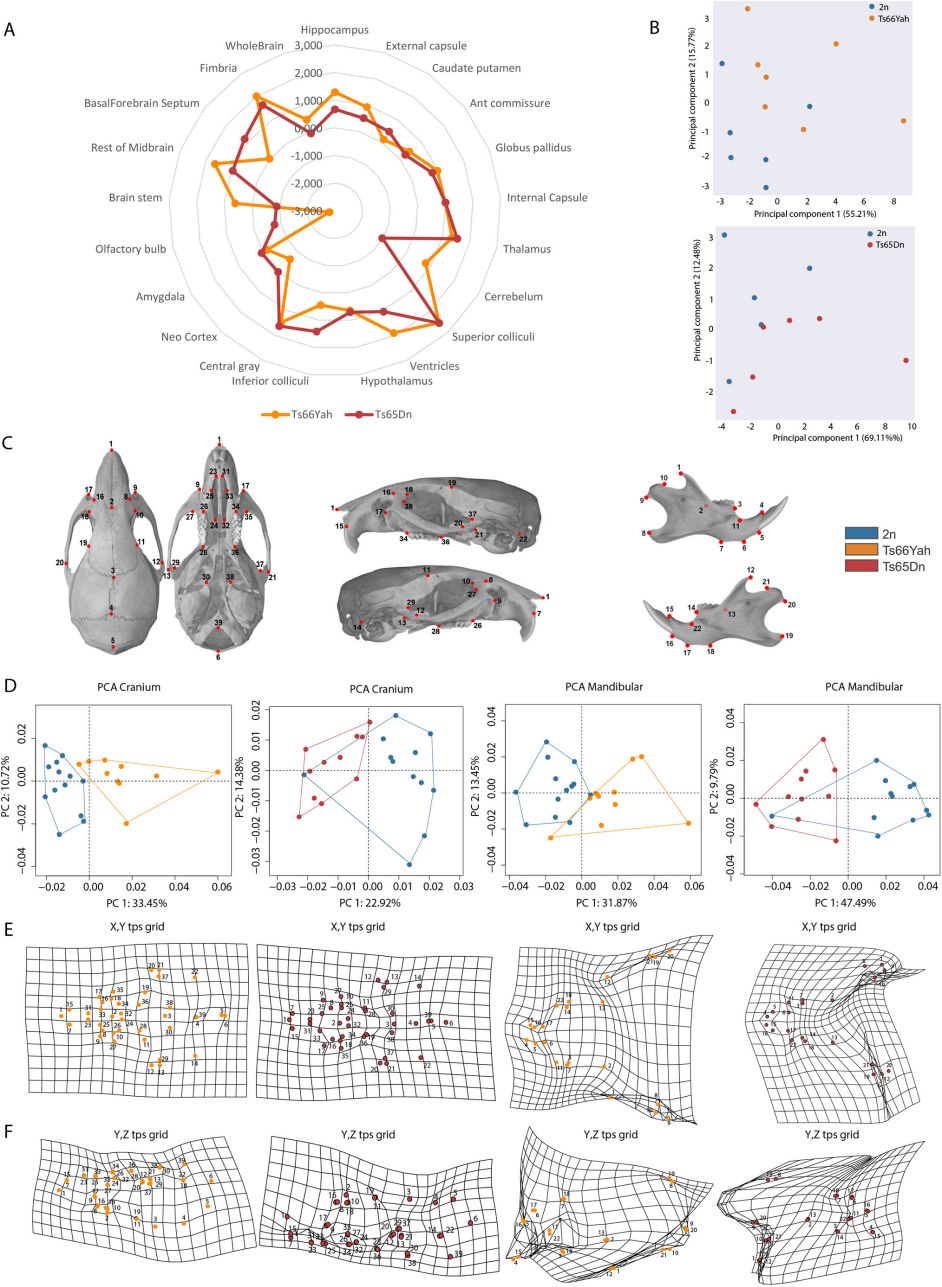
**Comparison of the morphological changes in the brain (magnetic resonance imaging) and skull (computed tomography scans) of male Ts66Yah and Ts65Dn mice.** We analyzed different brain regions/structures, taking into consideration the whole brain volume. (A) The *z*-score was calculated as the mean of control (wt) mice minus the mean of transgenic mice divided by each type of wt and trisomic (Ts) mouse. Changes were similar between the Ts66Yah (2n, *n*=6; Ts, *n*=7; males) and Ts65Dn (2n, *n*=5; Ts, *n*=6; males), although the amplitude of the changes was less drastic in Ts66Yah mice than in Ts65Dn mice. (B) PCA analysis indicated that Ts65Dn mice were more affected than their respective wt mice compared to the Ts66Yah mice. (C) The 39 landmarks used for craniofacial analysis. (D-F) Craniofacial analysis. (D) PCA analysis after generalized Procrustes indicated that, for cranial skull and mandibular, the 2n (*n*=13) and the Ts66Yah (*n*=10) male groups were well separated, whereas the Ts65Dn (*n*=15) males were less well separated from their control 2n littermates (*n*=16). (E,F) The shape differences between the means of groups was visualized graphically by obtaining the average landmark coordinates for each group and the overall mean and plotting the differences as thin-plate spline transformation grids for the two axes. The *x*-*y* axis was less affected than the *y*-*z* axis for both skull and mandible.

To detect brain morphological alterations of specific regions in the two DS models, we conducted a magnetic resonance imaging (MRI) study. We did not observe statistically significant changes for the whole-brain volume. Nevertheless, to be as accurate as possible, we considered the whole-brain volume and performed a *z*-score standardization (Fig. 5). Globally, we observed that changes in the morphology of specific brain structures as well as the direction of the changes (increase or decrease of volume) were the same in both DS lines. However, the amplitude of the changes was less severe, but statistically significant, in Ts66Yah than in Ts65Dn (for example, at ventricles, the rest of the midbrain, fimbria, superior colliculi; Fig. 5; Fig. S7). PCA (Fig. 5B) comparing mutants with their respective 2n controls for both lines pointed to a more pronounced difference between the Ts65Dn mutants and their controls than for the Ts66Yah mutants and their controls.

People with DS have very specific craniofacial changes, which have also been found in Ts65Dn (Richtsmeier et al., 2000; Starbuck et al., 2014). Thus, we investigated the Ts66Yah mice, searching for similar craniofacial changes using a landmark-based analysis (Fig. 5C; Table S6). PCA computed on Euclidian distance calculated from well-defined landmarks (Fig. 5D) showed that control and trisomic mice were well separated in the Ts66Yah line compared to the Ts65Dn line. The shape differences between groups for skulls and mandibles can be visualized graphically, showing that the deformation was more pronounced in the *y*-axis than in the *x*-axis (Fig. 5E,F), with the Ts65Dn skull smaller in global size than the Ts66Yah skull and with more pronounced deformations. Altogether, our brain and skull morphology results demonstrated convergence with DS features in both Ts66Yah and Ts65Dn, but with additional changes due to the presence of trisomy of the *Scaf8-Pde10a* genetic interval in Ts65Dn.

### Ts66Yah and Ts65Dn models show a strong tissue-specific dysregulation profile in the entorhinal cortex (EC) and hippocampus (HIP), with differences in functional alterations

**Fig. 6.**
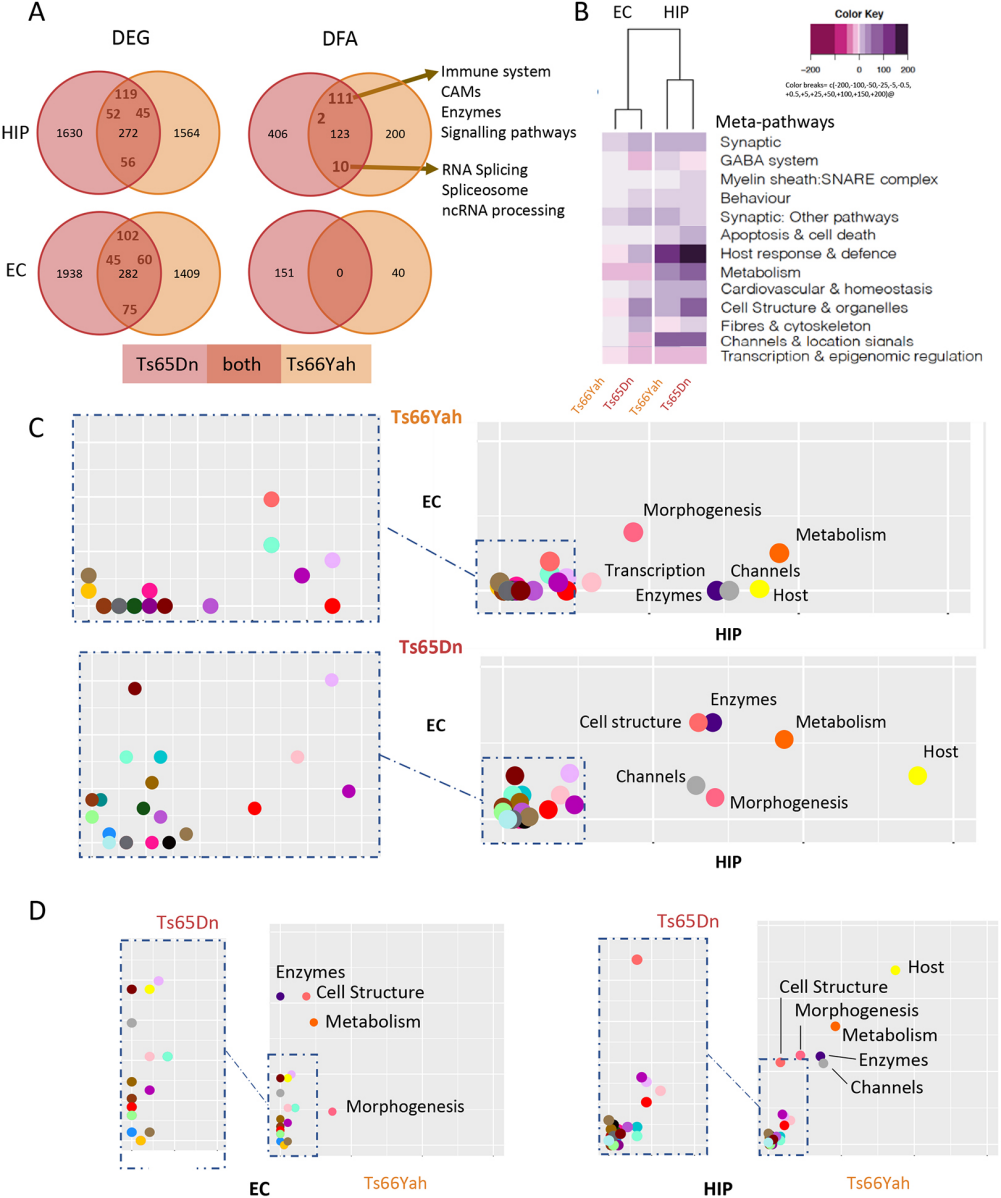
**Functional analysis of expressed genes and pathways altered in the Ts66Yah compared to Ts65Dn DS models in hippocampi (HIP) and entorhinal cortices (EC) from male mice.** (A) Venn diagrams for the differentially expressed genes found in common between the Ts66Yah and Ts65Dn HIP and EC. Right panel highlights the model-specific and common pathways altered between Ts66Yah and Ts65Dn HIP samples in the upper part and the EC datasets of both the Ts65Dn and Ts66Yah models in the lower part. DFA, differential functional analysis. (B) Heatmap representation of the number and regulation sense of the meta-pathways found in the Ts65Dn and Ts66Yah HIP and EC. The color key breaks represent the number of pathways within the meta-pathways. (C) Scatter plot showing the inter-tissue comparison of the percentage of pathways included on each meta-pathway, normalized by the total number of unique pathways per meta-pathway for Ts66Yah (upper panel) and Ts65Dn (lower panel) on the *x*-axis and *y*-axis, representing the HIP and EC, respectively, for the Ts66Yah or Ts65Dn models,. (D) Similar representation showing the inter-model comparison with the percentage of pathways included on each meta-pathway (group of pathways) normalized by the total number of unique pathways per meta-pathway found in the HIP of Ts66Yah (*x*-axis) compared to that of Ts65Dn (*y*-axis).

The HIP and EC are two brain regions contributing to NOR (Cohen and Stackman, 2015; Leal and Yassa, 2015; Schultz et al., 2015). Because NOR is affected in both the Ts66Yah and Ts65Dn DS models (Duchon et al., 2021), we wanted to identify genes and molecular pathways altered in the two models and the two brain regions. Thus, we analyzed the expression profiles in both the Ts66Yah and Ts65Dn DS models by focusing on the HIP and EC using RNA sequencing (RNA-Seq). After normalizing the raw data, we were able to confirm the quality of all the biological replicates as the samples clustered well as a function of their genotype using PCA clustering and the Euclidian distance of all the differentially expressed genes (DEGs) identified (Fig. S8A,B). Then, we performed differential expression analysis (DEA) and identified 1902 and 2220 DEGs in Ts66Yah HIP and EC, respectively, and 1836 and 1691 DEGs in Ts65Dn HIP and EC, respectively (Fig. S8C, Table S7). All the DEGs found in both tissues were spread along all the chromosomes (Table S8). Interestingly, the misregulation was very specific in the HIP and the EC, with only 417 and 382 genes found misregulated in both regions (HIP∩EC) in Ts66Yah and Ts65Dn, respectively. First, in the Ts66Yah model, the 417 genes identified as being commonly dysregulated in both tissues were spread along all the chromosomes, and more than 50% were non-coding genes and pseudogene genomic elements. Of these, 72 had a tissue-specific regulatory sense, and more than 60% were non-coding genes and pseudogenes. Moreover, only 54 genes from Mmu16 were identified as DEGs in both tissues. We found similar results for Ts65Dn, with 382 common genes between the regions and 34 from Mmu16. Of these, 45 have a tissue-specific sense of regulation, and more than 70% are non-coding genes and pseudogenes. Thus, with only ∼20% of genes shared between the two brain regions, we concluded that strong tissue-specific dysregulation is found in both the Ts65Dn and Ts66Yah models. More globally, we compared the number of DEGs found in both tissues in Ts65Dn versus Ts66Yah (Fig. 6A). We found 272 common DEGs in the HIP and 282 in the EC (28 are triplicated in Ts65Dn); of these, 97 and 105 DEGs follow opposing regulatory senses in both models for the HIP and EC, respectively (34 are triplicated in Ts65Dn). Those opposing regulated genes could very well be responsible for the differences observed in behavioral analyses.

Then, we checked the fold change in triplicated genes along the chromosome regions on Mmu16 and Mmu17. We detected a similar fold change for most of the genes located in the Mmu16 region, homologous to Hsa21, in the HIP and EC (Fig. S9, Table S9). Out of the 133 protein-coding genes (PCGs) that are triplicated and located in the segment on Mmu16, 90 and 66 showed compensated expression in the Ts66Yah HIP and EC (67% and 49% of the triplicated PCGs), respectively, whereas 78 and 100 genes, respectively, were compensated in the Ts65Dn tissues (58% and 75%, respectively; Table S7), thereby supporting previous observations in which approximately half of the triplicated genes are compensated or partially compensated in partial DS models, with a strong effect of the tissue/organ analyzed (Aït Yahya-Graison et al., 2007; Duchon et al., 2021). As expected with the dose effect, the fold changes in the 51 genes (PCGs and non-coding genes) located on the proximal part of Mmu17 were around 1.02±0.25 and 1.01±0.25 (mean±s.d.), respectively, in the Ts66Yah HIP and EC, while they were between 1.54±1.22 and 1.63±1.2 in the Ts65Dn HIP and EC, respectively. Altogether, the Spearman correlation comparing trisomic expressed genes (TEGs) from Mmu16 in the HIP or EC in both models was low (between 26% and 30%) and was completely lost when looking at TEGs from the centromeric region of Mmu17 (Fig. S8D). Nevertheless, a few genes from this centromeric interval of Mmu17, triplicated in Ts65Dn but not in Ts66Yah, showed a distinct pattern of expression in the Ts66Yah HIP. As expected, nine genes – *Ezr*, *Pde10a*, *Scaf8*, *Tiam2*, *Tfb1m*, *Zdhhc14*, *Synj2*, *Serac1* and *Gtf2h5* – were differentially expressed in Ts65Dn but not in Ts66Yah; two genes, *Ccr6* and *Tagap*, upregulated in Ts65Dn, were downregulated in Ts66Yah.

We verified, by quantitative reverse transcription PCR (RT-qPCR), the expression of several genes in the HIP of control and mutant mice. We confirmed the overexpression of *Dyrk1a*, *Sod1* and *Sh3bgr*, as expected, in both models. We also verified that *Pde10a* and *Ezr* from the Mmu17 region were only upregulated in Ts65Dn HIP (Fig. S10), while *Ccr6* was downregulated in Ts66Yah HIP and upregulated in Ts65Dn HIP. Finally, we also identified genes from Mmu16, syntenic to Hsa21, with an at least 0.5-fold increase in Ts65Dn HIP compared to Ts66Yah HIP (*Kcne1*, *Kcnj15*, *Map3k7cl*), and in Ts65Dn EC compared to Ts66Yah EC (*Sh3bgr*, *Itgb2l*, *Ripk4* or *Ripply3*), suggesting that the increased dosage of the centromeric Mmu17 genes interferes with the regulation of these genes.

On the functional side, we identified 323 and 493 pathways altered in the HIP of the Ts66Yah and Ts65Dn models, and 40 and 135 pathways in the EC of both models (Tables S7 and S10). This observation suggested a more profound impact in the HIP versus the EC, and in the Ts65Dn versus the Ts66Yah conditions (Fig. 6B). In addition, a milder effect was found in both DS models in the EC samples compared to the HIP samples, and no common pathway was found between Ts65Dn and Ts66Yah in the EC (Fig. 6A). Next, we identified common alterations per brain region in both DS models; in the HIP, 123 common pathways were altered following the same regulatory sense. Of these, 111 were upregulated and were pathways involved mainly in the immune system, cell adhesion molecule and signaling pathways. However, only ten were downregulated, linked to RNA splicing and ncRNA processing (Fig. 6C,D; Table S10). The heatmap showing the average number of pathways contributing to meta-pathways with regulatory orientation for the Ts65Dn and Ts66Yah HIP and EC models supports strong brain-region-specific alteration observed in the DEA in both models (Fig. 6B). Interestingly, the samples were clustered by brain regions and not by models. Inside each meta-pathway, we identified those that showed a different intensity in the number of pathways altered or in the regulatory sense between both DS models.

In addition, the majority of altered pathways in the HIP for both models were upregulated as expected (Duchon et al., 2021). A lower number of pathways grouped on each meta-pathway was identified in the EC than in the HIP. As such, the HIP showed a higher number of altered pathways and a higher deregulation of ‘host response’, ‘channels’, ‘metabolism’ and ‘cell structure and organelles’. Interestingly, the effect was stronger in Ts65Dn compared to Ts66Yah mice (Fig. 6B). To better understand the region-specific alterations, we compared the HIP and EC meta-pathway maps for each model (Fig. 6C) and between the DS models (Fig. 6D). A few meta-pathways, such as ‘metabolism’, ‘morphogenesis and development’, ‘channels and location signals’ and ‘enzyme activity’, were strongly affected in both DS models, with slightly different levels in the HIP and EC (Fig. 6C). Surprisingly, ‘cell structure and organelle’ was more strongly affected in Ts65Dn than in Ts66Yah mice, and ‘host response’ was affected in both the Ts66Yah and Ts65Dn HIP but only in the Ts65Dn EC. Moreover, when we compared the HIP and EC alteration map (Fig. 6D) between Ts66Yah and Ts65Dn, we observed that Ts65Dn showed stronger alterations than Ts66Yah in the ‘host and immune response’-, ‘morphogenesis’- and ‘metabolism and cell structure’-related pathways in the HIP. In addition, the alteration in pathways in the EC was higher in the Ts65Dn model, considering the number of total pathways found altered (151 in Ts65Dn, 40 in Ts66Yah), and involved mainly the ‘metabolism’, ‘enzymes’, ‘cell structure’, ‘fibers and cytoskeleton’ and ‘host and immune response’ meta-pathways.

**Fig. 7.**
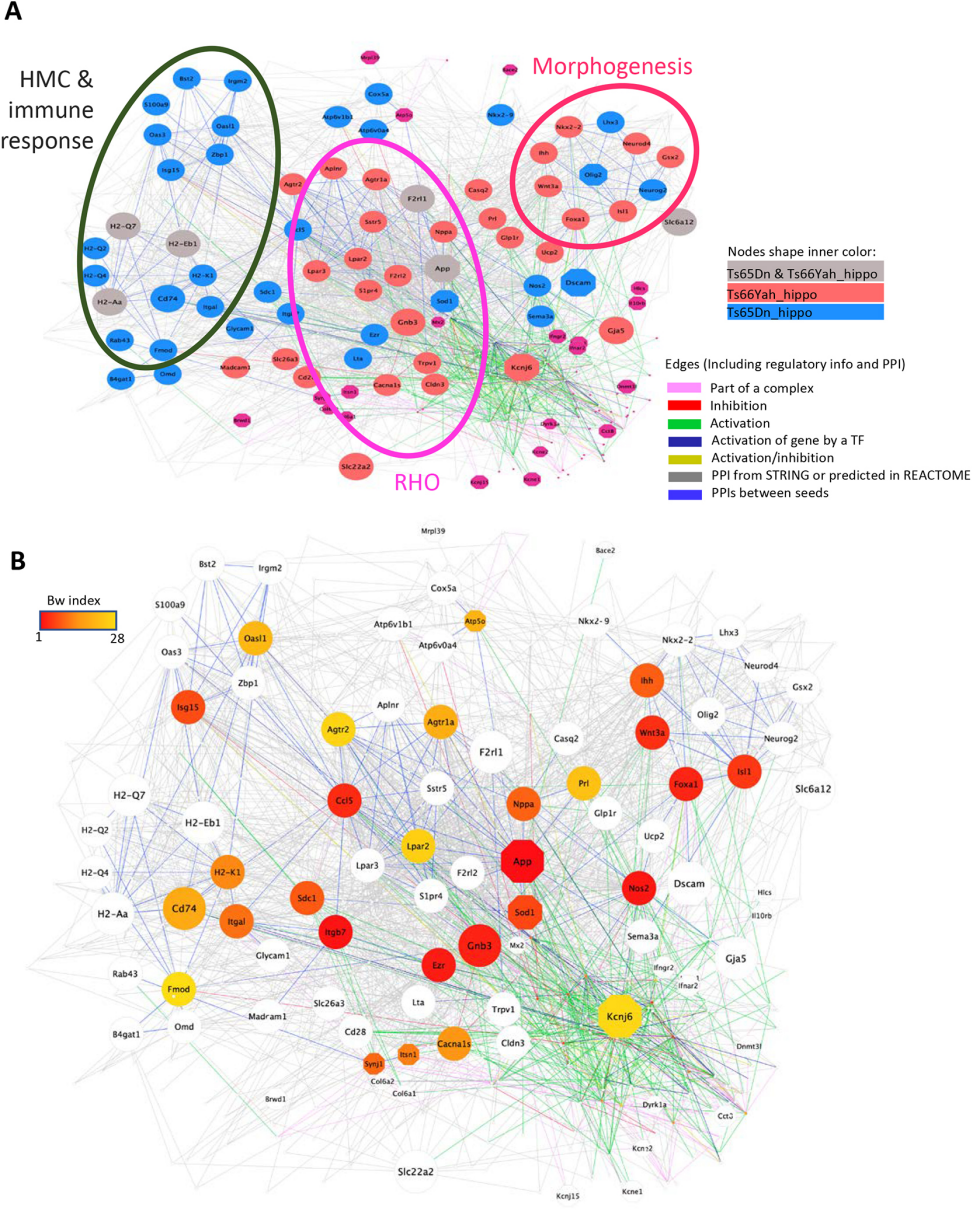
**Central protein–protein interaction and regulatory gene connectivity network (RegPPINet) involved in the synaptic meta-pathway identified in Ts65Dn and Ts66Yah males, highlighting the top 30 genes identified by betweenness centrality analysis.** (A) Central RegPPINet of genes involved in the synaptic meta-pathway identified in Ts65Dn and Ts66Yah, highlighting the main subnetworks found. (B) The same central RegPPINet, highlighting in color from red to yellow the proteins more central for the communication flow over the network identified by the centrality analysis using the betweenness index.

To further understand the nature of the Ts65Dn and Ts66Yah phenotype divergence, we built a regulatory and protein–protein interaction (PPI) network, noted RegPPINet, using all the genes identified by the generally applicable gene-set enrichment (GAGE) pathway analysis (Luo et al., 2009), for Ts65Dn and Ts66Yah, and known to contribute to the synaptic meta-pathway group. After performing a betweenness-based centrality analysis on the joined Ts65Dn–Ts66Yah RegPPINet, we identified three main subnetworks (Fig. 7A): the ‘MHC-immune response’ gathered members of the major histocompatibility complex (MHC) and of the IFN response, and is almost Ts65Dn specific, whereas the two others, ‘RHO’ and ‘morphogenesis’, were more affected in the Ts66Yah model. Remarkably, a few proteins more central to the network are not encoded in Hsa21 (Fig. 7B), such as CCL5, EZR, FOXA, GNB3, ISL1, ITGB7, NOS2 and WNT3A. Furthermore, our network analyses in Ts66Yah and Ts65Dn support the role of the DS subnetworks linked to RHOA, SNARE proteins (VAMPs and Sec protein interactors), DYRK1A and NPY (Fig. S11), which are deeply intertwined, as previously identified in seven other DS mouse models (Duchon et al., 2021).

Then, we focused on the Ts66Yah EC and HIP RegPPINet. Strikingly, the pathway dysregulation in the EC unraveled a mild effect of the trisomic model over this tissue structure with 40 pathways affected, mainly morphogenesis, metabolism, cell structure and synaptic meta-pathways (Fig. 6; Fig. S12A). The Ts66Yah EC RegPPINet revealed several well-connected subnetworks (Fig. S12B) linked mainly to ‘morphogenesis’, ‘metabolism’ and ‘host response and defense’ (Fig. S12C). Conversely, a more severe dysregulation with 323 altered pathways was revealed in the HIP (Tables S7 and S10, Fig. S13A). Although less functional alteration was found in Ts66Yah than in Ts65Dn, the stronger changes were found at the level of the ‘host response and defense’ and ‘metabolism’ meta-pathways, with important relations to genes also belonging to morphogenesis- and synaptic-related pathways (Fig. S13D). The network analysis identified several central genes distributed over all the subnetworks (Fig. S13B,C), highlighting the strong connectivity and importance of each subnetwork in the Ts66Yah HIP. Additionally, in the most closely connected subnetwork including the top central genes, we identified *App*, *Lpar3* and *Lpar2*, which were closely connected (Fig. S13C). Furthermore, most of the genes contributed to the alteration in different meta-pathways or functionalities due to gene multifunctionality, again pointing out the relevance of improving our understanding of comorbidity phenotypes in DS (Fig. S13D).

## DISCUSSION

Here, we report the general characterization of the new Ts66Yah line and compared several outcomes with the well-known Ts65Dn DS mouse model. Ts66Yah is certainly more genetically valid for DS studies than Ts65Dn as it does not contain trisomy of the *Scaf8-Pde10a* centromeric Mmu17 region unrelated to Hsa21. The general transmission of the recombined Ts66Yah chromosome was similar to that of the Ts65Dn chromosome (Moore et al., 2010), showing that the centromeric part of Mmu17 does not play a major role in the sterility of trisomic males. The male germline transmission property in the Ts66Yah may have arisen from the construction of the models and should be monitored to see if it is maintained over generations. Interestingly, sperm quality was also similar in the two models; probably the fertility issue is more connected to sperm fertility rather than to the parameters of concentration, mobility and velocity checked here.

More importantly, both DS models were found to replicate very classical DS cognitive features. Similarities were found in behavioral phenotypes in the two models for the nesting, object recognition and spontaneous alternation tests. In particular, defects in object recognition have been observed in many different laboratories for the Ts65Dn line and have been replicated in two independent laboratories for Ts66Yah. Thus, we must consider this phenotype as a very robust one. It would be very interesting to see if other phenotypes found in the Ts65Dn are also reproduced in Ts66Yah. For example, the impairment in spatial learning was found in the two models during the MWM test, but no spatial memory phenotype was observed in Ts66Yah compared to Ts65Dn. In addition, we found that the Ts66Yah line did not reproduce the anxiety and hyperactivity phenotypes observed in the open field with the Ts65Dn line. Similarly, circadian activity was normal in Ts66Yah, whereas we found increased activity for Ts65Dn, as observed previously in light–dark condition (Ruby et al., 2010). Although hyperactivity may be influenced by gene–environment interaction (Itohara et al., 2015), increased locomotor activity is common to many mutant mouse models, i.e. 678 significant genes out of 8641 mutants tested by the International Mouse Phenotyping Consortium. Hyperactivity could be a direct consequence of abnormal involuntary movement or motor coordination, or indirect consequence, such as being associated with stress, being in a novel environment or being influenced when raised by or in contact with mutant individuals. This increased activity has a major impact on behavioral testing in Ts65Dn, as shown recently by the contribution of distance traveled and thigmotaxis to novel object discrimination (Sierra et al., 2021). This phenomenon should depend on genes located in Mmu17 either directly or indirectly, because Ts66Yah was not affected. According to the Mouse Genome Informatics database, mutants from five genes of the region – *Arid1b*, *Cep43*, *Nox3*, *Pde10a* and *Serac1* – are linked to activity, motor and stereotypic behavior, and are strong candidates for which overdosage may enhance the activity of the Ts65Dn mouse model. Similarities were also observed in brain and craniofacial morphologies, with convergence found in both DS models. Nonetheless, the severities of the trisomy in these two features were slightly different: the Ts65Dn brain was larger than the Ts66Yah one in many different regions, while the shape of the cranium was affected differently, with more severe brachycephaly and increased mid-face shortening in Ts66Yah compared to Ts65Dn, a phenotype closer to human DS features.

Another intriguing result came from the expression analysis in the two brain regions, the HIP and EC, which are both involved in learning and memory. First, there was little overlap in the DEGs in the two models, and although some pathways were found dysregulated similarly in the HIP of both models, no real pathway convergence was observed in the EC, with more severe impact for Ts65Dn compared to Ts66Yah, presumably due to the trisomy of the centromeric region of Mmu17.

Altogether, the results from learning and behavior, craniofacial and brain morphologies, and gene expression in two brain regions highlighted the interference of the *Scaf8-Pde10a* trisomy in the Ts65Dn phenotypes. Considering the breeding of the two models on F1B6C3B, it would be highly recommended to genotype for the zygosity of the *Scaf8-Pde10a* region that can be found as heterozygous for B6 and C3B alleles, or homozygotes for both alleles. In addition, it would be important to control for any recombination event in the small region to reduce the risk of generating a subline with specific genotype in this region. Interestingly, some genes, such as *Ezr*, encoded in the Mmu17 interval, is known to interact with the RHOA pathway and is able to downregulate RHOA activity. Reversely, RHO can activate *Ezr* activity and lead to new specific phenotypes during neuritogenesis (Yonemura et al., 2002; Matsumoto et al., 2014; Hatano et al., 2018). *Ezr* was found to be a major hub in the top five influential HIP genes. We may therefore hypothesize that the *Ezr* overexpression found in the Ts65Dn brain acts on the downregulation of the RHOA pathway found altered in DS models (Duchon et al., 2021). For the lower-activity phenotypes found in Ts66Yah compared to Ts65Dn, *Serac1*, *Pde10a* and *Ccr6*, the loss of function of which impaired locomotor activity, are interesting candidates, while the defect in *Kcne1* induced hyperactivity. This means that the doses of those genes may explain the hyperactivity found to be more severe in Ts65Dn than in Ts66Yah.

In the network analysis of both the Ts65Dn and Ts66Yah HIP, we confirmed the dysregulation in the immune response, RHOA and morphogenesis pathways, as detected previously in several DS models (Duchon et al., 2021). From this common network, five proteins, encoded by the Mmu16 region homologous to Hsa21, appeared to have stronger effects, based on the betweenness centrality index (APP, SOD1, KCNJ6, SYNJ1, ITSN1), whereas others had less impact (ATP5O, BACE2, BRWD1, DYRK1A, DSCAM, HLCS, IL10RB, INFAR2, INFGR2, KCNE1, KCNE2, KCNJ15, MRPL39, MX2, OLIG2), supporting the multigenic dimension in DS models. Interestingly, genes from other regions homologous to Hsa21 were also detected in the HIP network, such as *Col6a1*, *Col6a2* and *Dnmt3l*.

The altered immune response has been reported previously in DS models (Ling et al., 2014; Guedj et al., 2016; Duchon et al., 2021). Here, we found that both the ‘synaptic’ and ‘host and immune response’ meta-pathways were closely connected by PPI between several members, and, more interestingly, some of the genes had dual synaptic and host response functionality, such as *App*, *Cldn19*, *F2rl1* (involved in the behavioral neurological phenotype), *Ihh*, *Trpv1*, *Nppa*, *Wnt3a*, *Isl1*, and a few genes coding for cell adhesion molecules, such as *H2-Q6* or *H2-Aa*.

Compared with the parental Ts65Dn line, the Ts66Yah line revealed the importance of the *Scaf8-Pde10a* trisomy. Interestingly, this region varies quite often in the mouse genetic inbred backgrounds used to generate and maintain the Ts65Dn minichromosome. Originally, the translocated minichromosome was from DBA/2J but then it was crossed to B6J and later bred to (C57BL/6J×C3H/HeJ)F1 (Davisson et al., 1993). Unfortunately, follow up of the DBA/2J alleles on Ts65Dn has never been done, although there was a proposal to use a single-nucleotide polymorphism in *Snx9* to detect the DBA/2J allele in the proximal part of the Ts65Dn minichromosome (Lorenzi et al., 2010). This variation in allelic composition of the *Scaf8-Pde10a* region may contribute to the fluctuation of the phenotypes observed from stock to stock in Ts65Dn (Shaw et al., 2020). Interestingly, Shaw et al. (2020) demonstrated several differences in the phenotypes of the 1924 and 5252 Ts65Dn mouse lines from different batches that have been further separated in 2010 to correct for retinal degeneration at The Jackson Laboratory: the 1924 subline keeping the original Ts65Dn genetic background and the 5252 rederived in a F1B6EiC3Sn.BLiAF1/J genetic background. Here too, no tracing of the Ts65Dn genotype was done in the sublines. Nevertheless, our results obtained here from the Ts65Dn 5252 subline (ordered in 2018) are coherent for the MWM memory phenotype found also in the cryopreserved stock in 2010, close to the time the 1924 and 5252 were separated. Thus, the loss of MWM memory phenotype observed in the Ts66Yah mouse compared to the Ts65Dn 5252 subline is indeed a direct consequence of the rescue of disomy of the *Scaf8-Pde10a* region. Nevertheless, more investigation should be done to control the other embryonic phenotypes found altered in the 5252 compared to other Ts65Dn sublines (Shaw et al., 2020).

As shown here, DS-related phenotypes in mouse models could be altered by change in genetic dosage of another genomic region, the *Scaf8-Pde10a* genetic interval. Conversely, this region may change penetrance or expressivity of features in individual with DS. Indeed, *SCAF8-PDE10A* on human chromosome 6 is also subjected to copy number variation according to the DECIPHER database (Firth et al., 2009). In human, the region is rearranged in the 3′ part (Fig. S1B) and contains the *ARID1B*, *SERAC1*, *GTF2H5*, *RPSH3* and *PDE10A* disease genes. Thus, the additional trisomy of *Scaf8-Pde10a* has a strong effect on the expression of several DS-related features in models. This phenomenon of additive effect due to genetic interaction has been observed in other copy number variation diseases (Pizzo et al., 2019). Overall, it highlights the need for complete evaluation of the genetic background in individuals with DS, to define potential interaction with other candidate disease-associated variants and to get a better understanding of the DS complexity. Our report introduced a new model with a freely segregating chromosome with a stronger genetic validity for DS, phenotypic variation in three main areas affected in individual with DS. Therefore, we hope that the DS research community will consider working with the newTs66Yah mouse model for DS, although this model remains a partial DS model because the triplicated *Mrpl39* to *Zbtb21* region encompasses 102/187 of the Hsa21 orthologous PCGs. The Ts66Yah model can be used to reduce interference with the Mmu17 region unrelated to Hsa21 in Ts65Dn, and obtain more refined analysis of DS over a lifetime, closer to the DS genetic condition.

## MATERIALS AND METHODS

All experiments were performed in accordance with the Directive of the European Parliament – 2010/63/EU, revising/replacing Directive 86/609/EEC – and with French Law (Decret no. 2013-118 01 and its supporting annexes, which came into force on 1 February 2013), relating to the protection of animals used in scientific experimentation. Y.H. was the principal investigator of this study (accreditation 67-369) in our animal facility (Agreement C67-218-40). Experimental procedures for the use of animals for research were approved by the Ministry of National Education, Higher Education and Research and with the agreement of the local ethical committee Com'Eth (no. 17) under the accreditation number APAFIS#13127-2018012210465339 v5 and APAFIS#21969-2019091215444738 v3.

### Mouse lines

The Ts65Dn [Ts(17^16^)65Dn] mice analyzed in the study, obtained from The Jackson Laboratory in 2018 from the 5252 subline (Shaw et al., 2020), were kept in an F1 B6C3B genetic background [with the C3B line as a C3H/HeN congenic line for the BALB/c allele at the *Pde6b* locus (Hart et al., 2005; Hoelter et al., 2008)]. Ts65Dn mice were genotyped according to published protocols (Duchon et al., 2011). Two primers were selected on both sides of the breakpoint to amplify a fragment of 396 bp (forward primer Fw_wtTs65Dn, 5′-GACTTAGTAAGAGCAAGTGGC-3′; reverse primer Rev_Ts65Dn, 5′-AGGTAGAAAGATGTGAGGACAC-3′), and a third primer was designed on the reverse strand of Mmu17 to amplify a fragment of 290 bp (5′-GGGCAACACTGGATCAATC-3′).

The generation of the new mouse line was done based on the Ts65Dn 1924 line (Shaw et al., 2020). Briefly, Ts65Dn male mice were selected and used for *in vitro* fertilization to produce fertilized eggs that were injected with two pairs of gRNAs (Fig. 1A), with one pair located on the centromeric region of Mmu17 and the other in the proximity of the breakpoint in the Ts65Dn minichromosome and on the Mmu16 region. The Ts66Yah line was obtained at PHENOMIN-ICS using CRISPR/Cas9 technology. The CRISMERE approach was selected to specifically obtain the deletion of the 6.2 Mb region located on the Ts65Dn minichromosome. sgRNAs were selected using the CRISPOR website (Haeussler et al., 2016) in order to generate double-strand breaks close to the Mmu17 centromere: gR65 and gR93 (GRCm39 17:3121550-3122030, at >43 kb from *Scaf8*) and 3.8 kb 3′ of the break point of the minichromosome; gR73 and gR74 (Mmu16:84151598-84152123, at 363 kb from *Mrpl39*) to avoid the repeats that are present at the Ts65 break point. Both gRNAs and Cas9 mRNA were synthesized by *in vitro* transcription (Birling et al., 2017). Microinjection of CRISPR reactive was performed in the pronuclei of fertilized oocytes obtained after *in vitro* fertilization of B6C3 F1 females with the sperm of aTs65Dn fertile male. Ninety-one oocytes were fertilized and microinjected with CRISPR/Cas9 reactive (the four sgRNAs and Cas9 mRNA). Fifty-three embryos that developed in two-cell embryos were reimplanted in three foster CD1 females. Fifteen pups were born and genotyped by junction PCR. One male pup had a clear new junction corresponding to the expected deletion. This male was still positive with the Ts65Dn junction, showing that this animal was mosaic. Noteworthily, out of the 15 pups born, only three had the Ts65 minichromosome junction. Droplet digital PCR was performed on the positive pup and a control pup with a probe located on *Dyrk1a* (located on Mmu16 and the minichromosome) and *Snx9* (located on Mmu17 and the minichromosome). Two copies of wt were detected with both probes on the wt control animal. On the new junction-positive animal, three copies were clearly detected with the *Dyrk1a* probe located on Mmu16, while a decrease to 2.5 copies of wt was observed with the *Snx9* probe located on Mmu17. These results confirmed the fact that the founder was mosaic and the intact Ts65 minichromosome was still present in some cells of the animal.

At 8 weeks of age, the male founder was bred with two wt C57BL/6NCrl females. Both wt females gave birth to a single litter with a total of 13 pups; no other pups were born afterwards. Five pups had the same new junction observed on the founder, but the Ts65 minichromosome junction was not observed on any of these F1 pups. For the five pups with the new junction, only two copies of wt were observed with the *Snx9* probe (Mmu17) and three copies were detected with both *App* and *Dyrk1a* probes (both located on Mmu16), confirming the presence of the engineered minichromosome in the pups. The recombined chromosome was selected to propagate upon breeding. As such, the full name of the mouse line should be Ts66YahIcs, shortened here to Ts66Yah.

Ts66Yah mice were genotyped with a specific primer encompassing the new break point between Mmu17 and Mmu16, with a forward primer Ts66Yah_wt-tg_up (5′-GGAAATATCGCACTTCACCAA-3′) and a reverse primer Ts66Yah_tg_dw (5′-CATGGGTCTGTGTGGTTTTCT-3′) to amplify a fragment of 322 bp. A third reverse primer was designed on the reverse strand of Mmu16 to amplify a wt fragment of 234 bp (5′-TCTAGGATCAGTGGGACTTTTGT-3′).

### Metaphase spread

Fibroblasts obtained from two Ts66Yah embryos were treated with 0.02 μg/ml colcemide for 2 h. Cells were then trypsinized, and the cell pellet was incubated in 0.56% KCl for 20 min in 5% CO_2_ at 37°C (hypotonic shock). Cells were then fixed in methanol–acetic acid 3:1 (v/v) for 20 min at room temperature, then washed three times with methanol–acetic acid and concentrated in a small volume. Drops of cell suspension were then plated on glass slides at 50°C. The cells were then allowed to dry and stained with 4% Giemsa as described previously (Codner et al., 2016). We analyzed 20 metaphase spreads.

### CGH

To confirm the increased copy number, we performed a CGH of the Ts65Dn and Ts66Yah models with their wt controls. For Ts65Dn, these CGH data have been previously published from the original 1924 Ts65Dn mouse line (Duchon et al., 2011); however, they were reused in this study for comprehensive comparison with CGH data from Ts66Yah. For Ts65Dn, the CGH was undertaken using NimbleGen mouse HD2 oligonucleotide arrays. Comparative analysis was done using DNA extracts from one wt animal that were fluorescently labeled with Cy5 and from one animal bearing the duplication labeled with Cy3. After sonication and labeling, DNA was hybridized to the CGH array, followed by washing the slide according to the manufacturer's instructions (Roche NimbleGen, Madison, WI, USA). Slides were scanned using a G2565 scanner at 3-lm resolution (Agilent Technologies, Palo Alto, CA, USA), and array images were analyzed using NimbleScan v2.5 software (Roche NimbleGen), with default parameters incorporating spatial correction. Arrays include 2,100,000 isothermal probes 50-75 bp in length with a median spacing of 1.1 kb throughout the genome.

For the Ts66Yah line, labeling was performed using a SureTag DNA Labeling Kit (Agilent Technologies) from 1 μg genomic DNA (based on Qubit assays). The samples were digested for 2 h at 37°C with restriction enzymes AluI and RsaI. After an inactivation step, the DNAs were denatured for 3 min at 98°C. The use of random primers and an exo-klenow fragment enabled the identification of samples with incorporation of dUTP coupled to Cy5 or Cy3. The targets thus synthesized were purified on 30 kDa columns (Agilent Technologies). wt and trisomic samples were labeled with Cy 3 and Cy5, respectively. Before carrying out the hybridization, the absorbances of the labeled DNAs were measured at 260 nm (DNA), 550 nm (Cy3) and 650 nm (Cy5) with a NanoDrop ND-1000 spectrophotometer. These values were used to assess performance and specific activity; the yield was between 9 μg and 14 μg. After labeling, the DNAs were hybridized on CGH 4×180K mouse slides (AMADID 027411, Agilent Technologies). Finally, the slides were scanned with a G2505C scanner (Agilent Technologies).

### Sperm analysis

Sperm analysis was done on 4- to 4.5-month-old males with IVOS (Hamilton Thorne) apparatus. After euthanasia, the vasa deferentia and cauda epididymis were dissected, and the sperm was sampled. The quality and quantity of semen was estimated according to four main parameters: concentration (millions/ml), motility (%), rapid cells (%) and progressivity (%). For this analysis, sperm was diluted 20 times in pre-warm (37°C) COOK^®^ solution (K-RVFE-50 COOK, Cook Medical, Bloomington, IN, USA). The suspension was gently agitated and placed for 3-4 min in a CO_2_ incubator before analysis with the IVOS system.

### Behavioral analysis laboratory 1 (Y.H. laboratory)

We generated several experimental animal cohorts by selecting mice from litters containing a minimum of two male pups. One cohort was used for nesting activity, working memory in the Y maze, exploration of a novel environment in the open field, spatial memory in the MWM, and circadian activity. Three independent cohorts of animals were used for sperm, craniofacial analysis and object recognition memory. Moreover, we also confirmed some phenotypes in another laboratory with a complete independent group of mice (17 2n males and 15 2n females compared to 11 Ts66Yah males and 14 Ts66Yah females). Independently of this line, a cohort of Ts65Dn mice was also built and assessed in the same behavioral pipeline, in order to permit direct comparison of both lines.

After weaning at 4 weeks of age, animals were sorted by litters into 39×20×16 cm cages (Green Line, Techniplast, Buguggiate, Italy) in which they had free access to purified water and food (D04 chow diet, Safe, Augy, France). The temperature was maintained at 23±1°C, and the light cycle was controlled as 12 h light and 12 h dark (lights on at 07:00). On testing days, animals were transferred to the antechambers of the experimental room 30 min before the start of the experiment. All the experiments were performed between 08:00 and 16:00. A resting period of 2 days to 1 week was used between two consecutive tests.

A series of behavioral experiments was conducted on male mice of ages ranging from 1.8 months at the start to 4.5 months for the last test. The tests were administered in the following order: circadian activity, nesting, Y maze, square open field, open field, NOR, MWM (standard hidden and reversal) and FC (contextual and cue). Behavioral experimenters were blinded to the genetic status of the animals. All the tests were performed with the experimenter out of the animal's sight. Further experiments for the evaluation of the spontaneous alternation and NOR were made in a different laboratory (Fig. S5), in which the two sexes were evaluated.

Circadian activity was measured to assess spontaneous activity behavior over the complete light/dark cycle. The actimeter (Imétronic, Pessac, France) used is composed of eight individual boxes (11×21×18 cm^3^), each of them equipped with an array of four parallel horizontal infrared beams and linked to a computer, allowing recordings of photocell beam breaks, providing automated measures of position and locomotor activity. Mice were put into cages at 11:00 on the first day and removed the next day at 19:00. The light cycle was controlled as 12 h light and 12 h dark (lights on at 07:00). The 32 h of testing were divided into three different phases: the habituation phase (from 11:00 to 19:00 on the first day); the night/dark phase (from 19:00 on the first day to 07:00 on the second day); and the day/light phase (from 07:00 to 19:00 on the second day).

The nesting test was performed by placing the mice individually in clean new housing cages 2 h before the dark phase, and the results were assessed the next morning. Normal bedding covered the floor to a depth of 0.5 cm. Each cage was supplied with a ‘nestlet’, a 5 cm square of pressed cotton batting. The nests were assessed on a five-point scale: 1, the nestlet was largely untouched (>90% intact); 2, the nestlet was partially torn up (50-90% remaining intact); 3, the nestlet was mostly shredded but often there was no identifiable nest site: <50% of the nestlet remained intact but <90% was within a quarter of the cage floor area, i.e. the cotton was not gathered into a nest but spread around the cage; 4, an identifiable but flat nest: >90% of the nestlet was torn up; the material was gathered into a nest within a quarter of the cage floor area, but the nest was flat with walls higher than mouse body height (curled up on its side) on less than 50% of its circumference; 5, a (near) perfect nest: >90% of the nestlet was torn up; the nest was a crater, with walls higher than mouse body height for more than 50% of its circumference (Deacon, 2006).

Short-term memory was assessed by recording spontaneous alternation in the Y-maze test (Hughes, 2004). The Y-maze test is based on the innate preference of animals to explore an arm that has not been explored previously, a behavior that, if occurring with a frequency greater than 50%, is called spontaneous alternation behavior (SAB). The maze was made of three enclosed plastic arms, each 40×9×16 cm, set at an angle of 120° to each other in the shape of a ‘Y’. The wall of each arm had a different pattern to encourage SAB. Animals were placed at the end of one arm (this initial arm was alternated within the group of mice to prevent arm placement bias), facing away from the center, and allowed to freely explore the apparatus for 8 min under moderate lighting conditions (70 lux in the centermost region). The time sequences of entries in the three arms were recorded (considering that the mouse enters an arm when all four paws were inside the arm). Alternation was determined from successive entries into the three arms on overlapping triplet sets in which three different arms are entered. The number of alternations was then divided by the number of alternation opportunities, namely, total arm entries minus one. In addition, total entries were scored as an index of locomotor activity.

A square open field was used to evaluate mice adaptation to a novel environment under stressful conditions. Mice were tested in an automated square open field (44.3×44.3×16.8 cm) made of PVC, with transparent walls and a black floor, and covered with translucent PVC (Panlab, Barcelona, Spain). The open-field arena was divided into central and peripheral regions and was homogeneously illuminated at 150 lux. Each mouse was placed on the periphery of the open field and allowed to explore the apparatus freely for 30 min. The distance traveled, the number of rearing episodes, and the stereotypes in the central and peripheral zones were recorded over the 30 min test session. Stereotypes is the number of samples in which the position of the subject is different from its position during the previous sample and equal to its position during the second sample back in time. The distance is the total distance (cm) traveled in the corresponding zone.

The NOR task is based on the innate tendency of rodents to explore novel objects over familiar ones (Bevins and Besheer, 2006). This test was done 24 h after the last open-field session performed in the same arena. On day 1, mice were free to explore two identical objects for 10 min. After this acquisition phase, mice returned to their home cage for a 24 h retention interval. Their memory was evaluated on day 2, using one familiar object (of those already experienced during the acquisition phase) and one novel object, which were placed in the arena with the mice free to explore the two objects for a 10 min period. Between trials and subjects, the different objects were cleaned with 70% ethanol to reduce olfactory cues. To avoid a preference for one of the two objects, the new one was different and counterbalanced between the different animal groups and genotypes. Similarly, to avoid a location preference, the emplacement of the novel object compared to the familiar one (left or right) was counterbalanced too. Object exploration was manually scored and defined as the orientation of the nose to the object, allowing a distance <1 cm. For the retention phase, the percentage of time exploring familiar versus novel objects was calculated to assess memory performance.

Spatial learning can be analyzed using the MWM task. This test was designed for the animals to learn to navigate a swimming tank (150 cm diameter) filled with opaque water, following the most direct path to a hidden submerged platform when starting from different, random locations around the perimeter of the tank. Two principal axes of the maze were defined, each line bisecting the maze perpendicular to one another to create an imaginary ‘+’. The end of each line demarcates four cardinal points – north (N), south (S), east (E) and west (W) – and four quadrants (NE, NW, SE and SW). The use of distal cues provides the most effective strategy to accomplish this task and avoid the aversive effect of cold water (22°C). This ability is controlled by HIP-dependent spatial cognition. The partially trisomic mice were trained in the standard version of the water maze as previously described (Vorhees and Williams, 2006; Arbogast et al., 2015). This standard version contains two different phases: a learning phase (using seven acquisition sessions) and a probe test (to assess memory performance). Each acquisition session contained four trials in which mice were placed at one of the starting locations in random order (N, S, E and W) and were allowed to swim until they located the platform situated in the target quadrant. Mice failing to find the platform within 60 s were gently guided and placed on it for 20 s (the same period of time as the successful animals). At the end of each learning and reversal phase, a probe test was done, with the platform removed, and the time spent in the target and non-target quadrants as well as the number of platform annulus crossings during 60 s were recorded.

At the end of this standard version, memory flexibility was tested with a reversal phase, in which the platform was positioned in the opposite quadrant (using four acquisition sessions) and a probe test. Reversal learning in the MWM reveals whether or not animals can extinguish their initial learning and acquire a direct path to the new goal position. Finally, a cue session was done to validate the test and to determine the swimming speed and visual ability using the visible platform, clearly indicated by a visible cue (black flag). All the trials were recorded with a video tracking system (Ethovision, Wageningen, The Netherlands). The swim paths for each mouse in each trial of the MWM test were categorized manually into one of the following search strategies: ‘thigmotaxis’, when mice have persistent swimming along the wall of the pool; ‘random’, when mice swim over the entire area of the pool in straight swims; ‘scanning’, when the search path is restricted to the central area of the pool; ‘chaining’, when mice swim in a circular manner at a fixed distance from the wall; ‘focal target’, when swim search is restricted to the target quadrant; ‘non-focal target’, when mice search the platform in an incorrect quadrant; and ‘directly’, when mice swim directly to the platform. Only ‘focal target’ and ‘directly’ are considered as spatial search.

To further challenge HIP-mediated cognitive behaviors, we used the FC test. FC is an associative learning paradigm for measuring aversive learning and memory, where a neutral conditioned stimulus (CS), such as light and tone, is paired with an aversive unconditioned stimulus (US) such as mild shock to the paw. Animals associate the spatial context cues with the CS concomitantly. After conditioning, the CS or the spatial context elicits a central state of fear in the absence of the US, translated into a reduced locomotor activity or total lack of movement (freezing) response (see Fig. S4B). Thus, immobility time is used as a measure of learning/memory performances (Crawley, 1999; Goeldner et al., 2009).

Experiments were conducted in four operant chambers (28×21×22 cm) with a metal bar floor linked to a shocker (Coulbourn Instruments, Allentown, PA, USA). Chambers were dimly lit with a permanent house light, and equipped with a speaker for tone delivery and infra-red activity monitor. The experimental procedure encompassed three sessions over 2 days, in which the activity/inactivity behavior was monitored continuously and the duration of inactivity per 2 s was collected. In day 1, for the conditioning session, the mouse was allowed to acclimate for 4 min, then a light/tone (10 kHz, 80 dB) CS was presented for 20 s and terminated by a mild shock in the paw (US) (1 s, 0.4 mA). After the paw shock, animals were left in the chamber for another 2 min. We defined total freezing time in the first 2 min and 4 min, and 2 min immediately after the paw shock as PRE1, PRE2 and POST, respectively. In day 2, the fear to context was tested by bringing back the mouse into the same chamber and allowing it to explore for 6 min without presentation of the light/auditory CS. The movements of the animal were monitored to detect freezing behavior consequence of recognizing the chamber as the spatial context (contextual learning). The total freezing time was calculated per 2 min time block as CONT2, CONT4 and CONT6. Finally, the cue testing was performed 5 h after the context testing. Animals were tested in modified conditioning chambers with walls and floor of different color and texture. The mouse could habituate for 2 min in the chamber, and then it was subjected to light and auditory cues for 2 min to evaluate conditioning fear. The total freezing time was calculated by 2 min block as PRECUE1, CUE1, PRECUE2 and CUE2.

### Behavioral analysis laboratory 2 (M.-C.P. laboratory)

Three-month-old Ts66Yah and 2n male and female mice were used in this study. The general health of mice was regularly checked throughout the experimental period. All experiments on animals were conducted in accordance with the ethical standards of French and European laws (European Communities Council Directive of 24 November 1986).

The SAB was assessed in a Y maze (wall height, 19.5 cm; arm length, 26 cm; arm width, 6.3 cm). Each arm was covered with different cues in its walls (arm ‘A’ with squares, arm ‘B’ with lines, arm ‘C’ with triangles). Mice were introduced into the maze alternating the arm of entry between mice. The 8 min test was video-recorded. An experimenter blind to the genotype analyzed the number of entries in each arm and the number of complete alternations. The percentage alternation was calculated as: [number of spontaneous alternations/(total number of arm entries−2)]×100. The total mice used for this test were 12 female Ts66Yah, 11 male Ts66Yah, 17 female 2n and 17 male 2n.

The NOR task was conducted in a V-maze apparatus (adapted from Catuara-Solarz et al., 2016) with black walls (Y-maze adapted; wall height, 19.5 cm; arm length, 26 cm; arm width, 6.3 cm). Each day, mice were introduced into the maze placed in the center (between the two arms of the V maze). The first day, mice were subjected to a 10 min habituation session during which they were allowed to explore the maze without any objects. The next day, mice went through a 10 min familiarization session in which two identical objects were situated at the end of each arm attached to the wall and the floor with adhesive tape. The next day, the recognition test session was conducted consisting of a 10 min trial in which a new object substituted one of the objects used at familiarization. The recognition of the new object was assessed by calculating the discrimination index (DI) using the following formula: DI=[(time exploring the novel object−time exploring the familiar object)/total exploration time)]. Two different pairs of objects were used. For each mouse, the type of object and the location of the novel object were randomized. The sessions were video-recorded and analyzed by an experimenter blind to the genotype. Mice exploring objects for less than 3 s in either of the phases were excluded from the analysis. When mice climbed on an object, the time the mice spent on the object was not counted as exploration in manual scoring. Between each trial, the arena and objects were cleaned with Aniospray (Dutscher, Bernolsheim, France) to reduce olfactory cues. The total mice used for this test were 11 female Ts66Yah, 11 male Ts66Yah, 14 female 2n and 13 male 2n. For 2n versus Ts66Yah comparisons, we used an unpaired two-tailed *t*-test.

### Statistical analysis

For each dataset, we performed the Shapiro–Wilk test and quantile-quantile plots to analyze whether the data were normally distributed and the Brown–Forsythe test to ascertain the homogeneity of variances. If the *P*-value was greater than the significance level (0.05), we assumed normality and equal variance. In this case, the statistical significance of differences between groups was inferred by an unpaired two-tailed *t*-test between genotype or repeated measures ANOVA (MWM, FC). The post hoc tests (Tukey test) were conducted only if *F* in repeated measures ANOVA achieved a 0.05 level. In the case of datasets in which the assumptions of normality or homogeneity of variances were not fulfilled, we used the Kruskal–Wallis non-parametric test. We performed a one-sample two-tailed *t*-test for the DI of the NOR versus no discrimination (0%), or for the percentage of spontaneous alternation versus 50% (hazard) in the Y maze, or 25% (hazard) for the probe test in the MWM.

The figures and statistical analyses for laboratory 2 were prepared/performed using GraphPad Prism 9. The phenotypes were compared between genotypes in male and female mice separately. The comparisons between 2n and Ts66Yah mice were made using an unpaired two-tailed *t*-test. The percentage of alternation and the discrimination index (NOR) was analyzed using a one-sample two-tailed *t*-test.

### Identifying the explanatory phenotyping variables in the trisomic lines

We used Gdaphen for ‘genotype discrimination using phenotypic features’ (Chidiac et al., 2021). Gdaphen is an R pipeline developed in our laboratory that allows the identification of the most important predictive qualitative and quantitative variables for genotype discrimination in phenotypic-based datasets without any prior hypothesis (available on github, https://github.com/YaH44/GDAPHEN).

Gdaphen also allows the identification of the most important predictor qualitative and quantitative variables for genotype discrimination in animal models of different diseases. We used Gdaphen (https://github.com/YaH44/GDAPHEN/releases/tag/Public) to identify the phenotypic explanatory variables recorded during the analysis more relevant to discriminate between mutant and wt genotypes from the Ts66Yah and Ts65Dn models. Moreover, we also identified which recorded variables were more relevant to discriminate between wts or mutants of each model to more deeply understand the differences and similarities in the relevance of the alterations in the phenotypic characterization performed for those mice models.

Gdaphen takes as input data an Excel table containing the information per animal in the rows and the variables recorded in the columns. As for some tests several variables were recorded, we grouped those variables with the same group label and identified the importance for the discrimination of each variable alone and the overall contribution of the group.

Pre-processing steps were carried out to get the data into shape for the analysis, after which Gdaphen was able to first identify the highly correlated variables (more than *r*=0.75) and remove them for downstream analysis. For example, in the sperm tests, several variables measured for the test were highly correlated with each other. The sperm percentage of rapid cells and the percentage of cell motility showed a coefficient of correlation *r*=0.987. Similarly, the percentage of rapid spermatozoa (‘sperm:rapid_cells’) was 0.97 correlated with the percentage of progressive cells (‘sperm:progressive’). Thus, we decided to keep only two variables: the sperm concentration (‘sperm:C°’, millions/ml) and the percentage of progressive cells (‘sperm:progressive’).

We decided to use two different classifiers to answer two different questions: (1) a generalized linear model (GLM) or GLM-Net model that will allow us to identify which phenotypic variables or ‘predicting variables’ are able to discriminate due to the fact that their linear combination influences the value of the dependent variable response; and (2) an RF, unsupervised algorithm that will be able to identify relevant phenotypic variables for the discrimination even though they may not originate from a linear distribution or exponential distribution family or have a linear relationship. Both functions are taken from the caret R and nnet R packages.

This method is able to deal with groups of both qualitative and quantitative variables recorded from the same individuals. The MFA performs a normalization or ‘weighting’ on each group by dividing all the variables belonging to the group by the first eigenvalue from the PCA of the group. Then, a PCA on all the weighted variables is applied so that we can identify the correlation between the grouped or ungrouped qualitative or quantitative variables, the principal component dimensions, and identify the individual coordinates of each observation on the PCA dimensions. The method is implemented using the MFAmix function from the PCAmixdata R package. Moreover, we chose a vectorization visualization approach like that implemented in PCAmixdata in which we included the cosine similarity distance to further highlight the parameters that follow the same trajectory as genotype. Consequently, these parameters contribute to the separation of the individual data on the same dimensions defined by their cosine similarity distance. We analyzed three different numbers of phenotypic predictor variables: (1) all phenotypic variables; (2) the phenotypic variables left after removing the highly correlated ones (correlation higher than 75%); and (3) the phenotypic variables contributing more than 30% in the discrimination after running the MFA using all the variables and observing the correlation between the quantitative ungrouped phenotypic variables with the main three dimensions of the PCA. Our reasoning was to try to decrease the noise added by variables that do not strongly contribute to the discrimination decrease the complexity of the model and the calculations, and increase the power on the discrimination because a lower number of variables is considered. Then, we calculated the variance of the data that we were able to explain using the first ten dimensions and the accuracy of the models to answer how well they can correctly predict each individual observation in the class of the dependent variable. We ran the Gdaphen pipeline to perform the genotype discrimination analyses on (1) Ts65Dn mutants and controls; (2) Ts66Yah mutants and their respective controls; and (3) Ts65Dn and Ts66Yah mutant and their respective control phenotypic data. In all these analyses, the model built using the phenotypic predictor variables known to contribute more than 30% to the discrimination was always able to explain a higher percentage of the variance in the data.

### MRI

Males from a dedicated cohort, aged between 3 and 4.5 months, were anesthetized and perfused with 30 ml of room temperature 1× PBS supplemented with 10% (% w/v) heparine and 2 mM ProHance Gadoteridol (Bracco Imaging, Courcouronnes, France) followed by 30 ml of 4% paraformaldehyde (PFA) supplemented with 2 mM ProHance Gadoteridol. Then, the brain structure was dissected and kept in 4% PFA supplemented with 2 mM ProHance overnight at 4°C. The next day, each specimen was transferred into 1× PBS 2 mM ProHance until imaging. Just prior to imaging, the brains were removed from the fixative and placed in plastic tubes (internal diameter, 1 cm; volume, 13 ml) filled with a proton-free susceptibility-matching fluid (Fluorinert^®^ FC-770, Sigma-Aldrich, St Louis, MO, USA). Images of excised brains were acquired on a 7T BioSpec animal MRI system (Bruker Biospin MRI GmbH, Ettlingen, Germany), with an actively decoupled quadrature-mode mouse brain surface coil for signal reception and a 86 mm birdcage coil for transmission, both supplied by Bruker. Two imaging protocols were used. The first protocol consisted of a three-dimensional (3D) T2-weighted rapid acquisition with relaxation enhancement (RARE). The parameters for this sequence were: repetition time, 325 ms; echo time, 32 ms; RARE factor, 6; interecho spacing, 10.667 ms; bandwidth, 92 kHz. The second imaging protocol consisted of a 3D T2*-weighted fast low angle (FLASH) sequence with the following parameters: repetition time, 50 ms; echo time, 25 ms; flip angle, 50°; bandwidth, 28 kHz. The output image matrices for both sequences were 195×140×90 over a field of view of 19.5×14.0×9.0 mm^3^, yielding an isotropic resolution of 100 μm, and were reconstructed using ParaVision 6.0.1. Each MRI image was segmented into 20 anatomical structures according to a multi-atlas label propagation framework. To this end, the ten manually segmented *in vitro* magnetic resonance images from the MRM NeAt Mouse Brain Database (https://github.com/dama-lab/mouse-brain-atlas/tree/master/NeAt) were considered (Ma et al., 2005). The image-processing pipeline consisted of the following steps: (1) a skull-stripping step was first performed using the tissue brain segmentation method provided in SPMMouse (https://github.com/dama-lab/mouse-brain-atlas/tree/master/NeAt) (Sawiak et al., 2009); (2) each magnetic resonance image was then corrected for bias field in homogeneity using N4ITK (Tustison et al., 2010); (3) the ten anatomically annotated images from the MRM NeAt Mouse Brain Database were registered in a deformable way on each mouse image using the ANTs registration toolbox (http://stnava.github.io/ANTs/) (Avants et al., 2008); and (4) anatomical labels were finally fused using the simultaneous truth and performance level estimation (STAPLE) (Warfield et al., 2004). By this way, the volumes of the 20 anatomical structures as well as the whole brain were computed for each image modality and each mouse. Finally, the volumes computed from the two image modalities were averaged out to obtain the final volume associated with each mouse. Morphological magnetic resonance images were compared across groups with a region-based analysis. The resulting region-based volume estimations were averaged out for each animal before the statistical analysis. For Ts65Dn, these data have been previously published by our group (Duchon et al., 2021) and were reanalyzed with the same analysis pipeline for comprehensive comparison of both models.

### Morphometrics

Mice used for the craniofacial study were sacrificed at 18 weeks of age, and carcasses were skinned, eviscerated and stored in 96% ethanol. Cranium morphology was assessed using a Quantum µCT scanner (Perkin Elmer, Waltham, MA, USA). All scans were performed with an isotropic voxel size of 20 µm, 160 µA tube current and 90 kV tube voltage. We applied a common approach to shape analysis named geometric morphometrics (GM) using the Geomorph software package in the R statistical computing environment (Adams and Otárola-Castillo, 2013). This approach used the coordinates of 39 relevant cranial landmarks that were recorded using Landmark software [Institute for Data Analysis and Visualization (IDAV) group at the University of California, Davis; Table S6]. A generalized Procrustes analysis was then used to superimpose the specimens on a common coordinate system by holding their position, size and orientation constant. From the Procrustes-aligned coordinates, a set of shape variables was obtained, which can be used in multivariate statistical analyses. Graphical methods were used to visualize patterns of shape variation. Taking a different approach, the PCA is a mathematical procedure that transforms a number of correlated variables into a number of uncorrelated variables. This permitted visualizing patterns of shape variation in shape space.

### RT-qPCR

cDNA synthesis was performed using a SuperScript^®^ VILO™ cDNA Synthesis Kit (Invitrogen, Carlsbad, CA, USA). PCRs were performed with TaqMan^®^ Universal Master Mix II and pre-optimized TaqMan^®^ Gene Expression assays (Applied Biosystems, Waltham, MA, USA), consisting of a pair of unlabeled PCR primers and a TaqMan^®^ probe with an Applied Biosystems™ FAM™ dye label on the 5′ end and minor groove binder (MGB) and nonfluorescent quencher (NFQ) on the 3′ end (listed in Table S4). mRNA expression profiles were analyzed by RT-qPCR using TaqMan™ Universal Master Mix II with UNG in a Realplex II Master Cycler (Eppendorf, Hamburg, Germany). The complete reactions were subjected to the following program of thermal cycling: one cycle of 2 min at 50°C, one cycle of 10 min at 95°C, 40 cycles of 15 s at 95°C and 1 min at 60°C. The efficiencies of the TaqMan assays were checked using a cDNA dilution series from extracts of HIP samples. Normalization was performed by amplifying four housekeeping genes (*Gnas*, *Pgk1*, *Actb* and *Atp5b*) in parallel and using the GeNorm procedure to correct the variations of the amount of source RNA in the starting material (Vandesompele et al., 2002). All the samples were tested in triplicate.

### Gene expression analyses

The HIP and EC from male Ts65Dn mice (*n*=6) and control littermates (*n*=6), and male Ts66Yah mice (*n*=6) and control littermates (*n*=5), at the age of 5-6 months, were isolated and flash frozen in liquid nitrogen. Total RNA was prepared using an RNA extraction kit (Qiagen, Venlo, The Netherlands) according to the manufacturer's instructions. Sample quality was checked using an Agilent 2100 Bioanalyzer (Agilent Technologies, Santa Clara, CA, USA).

The preparation of the libraries was done by the GenomEast platform, a member of the ‘France Génomique’ consortium (ANR-10-INBS-0009), using the TruSeq Stranded Total RNA Sample Preparation Guide - PN 15031048. Total RNA-Seq libraries were generated from a minimum of 150-300 ng total RNA using a TruSeq Stranded Total RNA LT Sample Prep Kit with Ribo-Zero Gold (Illumina, San Diego, CA, USA), according to the manufacturer’s instructions.

The molecules extracted from the biological material were polyA RNA. Whole-genome expression sequencing was performed by the platform using Illumina Hiseq 4000 and generating single-end RNA-Seq reads of 50 bp length. The raw sequenced reads were aligned by Hisat2 against the GRCm38.v99 mouse assembly. In total, 55,385 ENSEMBL gene IDs were quantified aligning with the GRCm38.v99 assembly. HTSeq-count was used to generate the raw counts. The downstream analyses were continued with in-house bash scripts and R version 3.6 scripts using FCROS (Dembélé et al., 2014) and DESeq2 (Love et al., 2014) packages to identify the DEGs. Raw reads and normalized counts have been deposited in Gene Expression Omnibus (GEO) (GSE213500 for Ts65Dn and GSE213502 for Ts66Yah).

We performed the differential functional analysis using GAGE pathway analysis (Luo et al., 2009) and grouped all the pathways into 25 functional categories (denoted meta-pathways). Then, to assess gene connectivity, we built a minimum fully connected PPI network (denoted MinPPINet) of genes known to be involved in the synaptic function as they were associated with synaptic pathways via the Gene Ontology (GO) (Ashburner et al., 2000) and Kyoto Encyclopedia of Genes and Genomes (KEGG) (Esling et al., 2015) databases. Regulatory information was also added to build the final RegPPINet. We used the betweenness centrality analysis to identify hubs, keys for maintaining the network communication flow.

To further study the genotype–phenotype relationship in those models, we combined the behavioral results and the RNA-Seq data to identify central genes altered in the models and linked to the phenotypes observed using the genotype–phenotype databases GO, KEGG and DisGeNET. First, we downloaded the list of experimentally validated genes known to be involved in hyperactivity or locomotion behavior from the human disease database DisGeNET (dataset: hyperactive behavior, C0424295, with 1263 genes) and annotated the genes with a high confidence ortholog in mouse. We added all the mouse genes involved in GO gene sets linked to locomotion or motor behavior (18 GO terms: GO:0007626, GO:0008344, GO:0031987, GO:0033058, GO:0035641, GO:0040011, GO:0040012, GO:0040013, GO:0040017, GO:0043056, GO:0045475, GO:0090325, GO:0090326, GO:0090327, GO:1904059, GO:1904060, GO:0036343, GO:0061744). Then, we queried our RNA-Seq data for these genes to identify those found deregulated in the datasets.

## Supplementary Material

10.1242/dmm.049721_sup1 Supplementary information

## References

[DMM049721C53] Adams, D. C. and Otárola-Castillo, E. (2013). geomorph: an r package for the collection and analysis of geometric morphometric shape data. *Methods Ecol. Evol.* 4, 393-399. 10.1111/2041-210X.12035

[DMM049721C1] Aït Yahya-Graison, E., Aubert, J., Dauphinot, L., Rivals, I., Prieur, M., Golfier, G., Rossier, J., Personnaz, L., Créau, N., Bléhaut, H. et al. (2007). Classification of human chromosome 21 gene-expression variations in Down syndrome: impact on disease phenotypes. *Am. J. Hum. Genet.* 81, 475-491. 10.1086/520000 17701894 PMC1950826

[DMM049721C54] Arbogast, T., Raveau, M., Chevalier, C., Nalesso, V., Dembele, D., Jacobs, H., Wendling, O., Roux, M., Duchon, A. and Herault, Y. (2015). Deletion of the App-Runx1 region in mice models human partial monosomy 21. *Dis. Model. Mech.* 8, 623-634. 10.1242/dmm.017814 26035870 PMC4457029

[DMM049721C2] Ashburner, M., Ball, C. A., Blake, J. A., Botstein, D., Butler, H., Cherry, J. M., Davis, A. P., Dolinski, K., Dwight, S. S., Eppig, J. T. et al. (2000). Gene ontology: tool for the unification of biology. *Nat. Genet.* 25, 25-29. 10.1038/75556 10802651 PMC3037419

[DMM049721C56] Avants, B. B., Epstein, C. L., Grossman, M. and Gee, J. C. (2008). Symmetric diffeomorphic image registration with cross-correlation: evaluating automated labeling of elderly and neurodegenerative brain. *Med. Image Anal.* 12, 26-41. 10.1016/j.media.2007.06.004 17659998 PMC2276735

[DMM049721C3] Aziz, N. M., Guedj, F., Pennings, J. L. A., Olmos-Serrano, J. L., Siegel, A., Haydar, T. F. and Bianchi, D. W. (2018). Lifespan analysis of brain development, gene expression and behavioral phenotypes in the Ts1Cje, Ts65Dn and Dp(16)1/Yey mouse models of Down syndrome. *Dis. Model. Mech.* 11, dmm031013. 10.1242/dmm.031013 29716957 PMC6031353

[DMM049721C61] Bevins, R. A. and , Besheer, J. (2006). Object recognition in rats and mice: a one-trial non-matching-to-sample learning task to study 'recognition memory. *Nat. Protoc.* 1, 1306-1311. 10.1038/nprot.2006.205 17406415

[DMM049721C57] Birling, M. C., Schaeffer, L., André, P., Lindner, L., Maréchal, D., Ayadi A., Sorg, T., Pavlovic, G. and Hérault, Y. (2017). Efficient and rapid generation of large genomic variants in rats and mice using CRISMERE. *Sci. Rep.* 7, 43331. 10.1038/srep43331 28266534 PMC5339700

[DMM049721C58] Catuara-Solarz, S., Espinosa-Carrasco, J., Erb I., Langohr, K., Gonzalez, J. R., Notredame, C. and Dierssen, M. (2016). Combined treatment with environmental enrichment and (-)-epigallocatechin-3-gallate ameliorates learning deficits and hippocampal alterations in a mouse model of Down syndrome. *eNeuro 3*, ENEURO.0103-16.2016. 10.1523/ENEURO.0103-16.2016 PMC5099603 27844057

[DMM049721C59] Chidiac, C., Xue Y., Muniz Moreno, M. D. M., Bakr Rasheed, A. A., Lorentz, R., Birling, M. C., Gaveriaux-Ruff, C. and Herault, Y. (2021). The human SCN10AG1662S point mutation established in mice impacts on mechanical, heat, and cool sensitivity. *Front. Pharmacol.* 12, 780132. 10.3389/fphar.2021.780132 34925037 PMC8671994

[DMM049721C4] Codner, G. F., Lindner, L., Caulder, A., Wattenhofer-Donzé, M., Radage, A., Mertz, A., Eisenmann, B., Mianné, J., Evans, E. P., Beechey, C. V. et al. (2016). Aneuploidy screening of embryonic stem cell clones by metaphase karyotyping and droplet digital polymerase chain reaction. *BMC Cell Biol.* 17, 30. 10.1186/s12860-016-0108-6 27496052 PMC4974727

[DMM049721C5] Cohen, S. J. and Stackman, R. W. (2015). Assessing rodent hippocampal involvement in the novel object recognition task. A review. *Behav. Brain Res.* 285, 105-117. 10.1016/j.bbr.2014.08.002 25169255 PMC7008635

[DMM049721C6] Concordet, J. P. and Haeussler, M. (2018). CRISPOR: intuitive guide selection for CRISPR/Cas9 genome editing experiments and screens. *Nucleic Acids Res.* 46, W242-W245. 10.1093/nar/gky354 29762716 PMC6030908

[DMM049721C62] Crawley, J. N. (1999). Behavioral phenotyping of transgenic and knockout mice: experimental design and evaluation of general health, sensory functions, motor abilities, and specific behavioral tests. *Brain Res.* 835, 18-26. 10.1016/s0006-8993(98)01258-x 10448192

[DMM049721C7] Davisson, M. T., Schmidt, C. and Akeson, E. C. (1990). Segmental trisomy of murine chromosome 16: a new model system for studying Down syndrome. *Prog. Clin. Biol. Res.* 360, 263-280. 2147289

[DMM049721C8] Davisson, M. T., Schmidt, C., Reeves, R. H., Irving, N. G., Akeson, E. C., Harris, B. S. and Bronson, R. T. (1993). Segmental trisomy as a mouse model for Down syndrome. *Prog. Clin. Biol. Res.* 384, 117-133. 8115398

[DMM049721C64] Deacon, R. M. J. (2006). Digging and marble burying in mice: simple methods for in vivo identification of biological impacts. *Nat. Protoc.* 1, 122-124. 10.1038/nprot.2006.20 17406223

[DMM049721C65] Dembélé, D. and Kastner, P. (2014). Fold change rank ordering statistics: a new method for detecting differentially expressed genes. *BMC Bioinformatics* 15, 14. 10.1186/1471-2105-15-14 24423217 PMC3899927

[DMM049721C9] Duchon, A. and Herault, Y. (2016). DYRK1A, a dosage-sensitive gene involved in neurodevelopmental disorders, is a target for drug development in Down syndrome. *Front. Behav. Neurosci.* 10, 104. 10.3389/fnbeh.2016.00104 27375444 PMC4891327

[DMM049721C10] Duchon, A., Raveau, M., Chevalier, C., Nalesso, V., Sharp, A. J. and Herault, Y. (2011). Identification of the translocation breakpoints in the Ts65Dn and Ts1Cje mouse lines: relevance for modeling Down syndrome. *Mamm. Genome* 22, 674-684. 10.1007/s00335-011-9356-0 21953411 PMC3224224

[DMM049721C11] Duchon, A., Del Mar Muñiz Moreno, M., Lorenzo, S. M., De Souza, M. P. S., Chevalier, C., Nalesso, V., Meziane, H., De Sousa, P. L., Noblet, V., Armspach, J.-P. et al. (2021). Multi-influential genetic interactions alter behaviour and cognition through six main biological cascades in Down syndrome mouse models. *Hum. Mol. Genet.* 30, 771-788. 10.1101/2020.07.08.193136 33693642 PMC8161522

[DMM049721C12] Esling, P., Lejzerowicz, F. and Pawlowski, J. (2015). Accurate multiplexing and filtering for high-throughput amplicon-sequencing. *Nucleic Acids Res.* 43, 2513-2524. 10.1093/nar/gkv107 25690897 PMC4357712

[DMM049721C13] Faundez, V., De Toma, I., Bardoni, B., Bartesaghi, R., Nizetic, D., de la Torre, R., Cohen Kadosh, R., Herault, Y., Dierssen, M., Potier, M.-C. et al. (2018). Translating molecular advances in Down syndrome and Fragile X syndrome into therapies. *Eur. Neuropsychopharmacol.* 28, 675-690. 10.1016/j.euroneuro.2018.03.006 29887288

[DMM049721C14] Fernandez, F., Morishita, W., Zuniga, E., Nguyen, J., Blank, M., Malenka, R. C. and Garner, C. C. (2007). Pharmacotherapy for cognitive impairment in a mouse model of Down syndrome. *Nat. Neurosci.* 10, 411-413. 10.1038/nn1860 17322876

[DMM049721C15] Firth, H. V., Richards, S. M., Bevan, A. P., Clayton, S., Corpas, M., Rajan, D., Vooren, S. V., Moreau, Y., Pettett, R. M. and Carter, N. P. (2009). DECIPHER: database of chromosomal imbalance and phenotype in humans using ensembl resources. *Am. J. Hum. Genet.* 84, 524-533. 10.1016/j.ajhg.2009.03.010 19344873 PMC2667985

[DMM049721C16] García-Cerro, S., Rueda, N., Vidal, V., Lantigua, S. and Martínez-Cué, C. (2017). Normalizing the gene dosage of Dyrk1A in a mouse model of Down syndrome rescues several Alzheimer's disease phenotypes. *Neurobiol. Dis.* 106, 76-88. 10.1016/j.nbd.2017.06.010 28647555

[DMM049721C50] Goeldner, C., Reiss, D., Wichmann, J., Kieffer, B. L. and Ouagazzal, A. M. (2009). Activation of nociceptin opioid peptide (NOP) receptor impairs contextual fear learning in mice through glutamatergic mechanisms. *Neurobiol. Learn. Mem.* 91, 393-401. 10.1016/j.nlm.2008.12.001 19100850 PMC4482120

[DMM049721C17] Goodliffe, J. W., Olmos-Serrano, J. L., Aziz, N. M., Pennings, J. L., Guedj, F., Bianchi, D. W. and Haydar, T. F. (2016). Absence of prenatal forebrain defects in the Dp(16)1Yey/+ mouse model of Down syndrome. *J. Neurosci.* 36, 2926-2944. 10.1523/JNEUROSCI.2513-15.2016 26961948 PMC4783496

[DMM049721C18] Guedj, F., Pennings, J. L., Massingham, L. J., Wick, H. C., Siegel, A. E., Tantravahi, U. and Bianchi, D. W. (2016). An integrated human/murine transcriptome and pathway approach to identify prenatal treatments for Down syndrome. *Sci. Rep.* 6, 32353. 10.1038/srep32353 27586445 PMC5009456

[DMM049721C51] Haeussler, M., Schönig, K., Eckert, H., Eschstruth, A., Mianné, J., Renaud, J. B., Schneider-Maunoury, S., Shkumatava, A., Teboul, L., Kent, J. et al. (2016). Evaluation of off-target and on-target scoring algorithms and integration into the guide RNA selection tool CRISPOR. *Genome Biol.* 17, 148. 10.1186/s13059-016-1012-2 27380939 PMC4934014

[DMM049721C19] Hart, A. W., Mckie, L., Morgan, J. E., Gautier, P., West, K., Jackson, I. J. and Cross, S. H. (2005). Genotype-phenotype correlation of mouse pde6b mutations. *Invest. Ophthalmol. Vis. Sci.* 46, 3443-3450. 10.1167/iovs.05-0254 16123450

[DMM049721C20] Hatano, R., Takeda, A., Abe, Y., Kawaguchi, K., Kazama, I., Matsubara, M. and Asano, S. (2018). Loss of ezrin expression reduced the susceptibility to the glomerular injury in mice. *Sci. Rep.* 8, 4512. 10.1038/s41598-018-22846-0 29540766 PMC5852236

[DMM049721C21] Heller, H. C., Salehi, A., Chuluun, B., Das, D., Lin, B., Moghadam, S., Garner, C. C. and Colas, D. (2014). Nest building is impaired in the Ts65Dn mouse model of Down syndrome and rescued by blocking 5HT2a receptors. *Neurobiol. Learn. Mem.* 116, 162-171. 10.1016/j.nlm.2014.10.002 25463650

[DMM049721C22] Herault, Y., Delabar, J. M., Fisher, E. M. C., Tybulewicz, V. L. J., Yu, E. and Brault, V. (2017). Rodent models in Down syndrome research: impact and future opportunities. *Dis. Model. Mech.* 10, 1165-1186. 10.1242/dmm.029728 28993310 PMC5665454

[DMM049721C23] Hoelter, S. M., Dalke, C., Kallnik, M., Becker, L., Horsch, M., Schrewe, A., Favor, J., Klopstock, T., Beckers, J., Ivandic, B. et al. (2008). “Sighted C3H” mice - a tool for analysing the influence of vision on mouse behaviour? *Front. Biosci.* 13, 5810-5823. 10.2741/3118 18508624

[DMM049721C55] Hughes, R. N. (2004). The value of spontaneous alternation behavior (SAB) as a test of retention in pharmacological investigations of memory. *Neurosci. Biobehav. Rev.* 28, 497-505. 10.1016/j.neubiorev.2004.06.006 15465137

[DMM049721C24] Itohara, S., Kobayashi, Y. and Nakashiba, T. (2015). Genetic factors underlying attention and impulsivity: mouse models of attention-deficit/hyperactivity disorder. *Curr. Opin. Behav. Sci.* 2, 46-51. 10.1016/j.cobeha.2014.09.002

[DMM049721C25] Kazuki, Y., Gao, F. J., Li, Y., Moyer, A. J., Devenney, B., Hiramatsu, K., Miyagawa-Tomita, S., Abe, S., Kazuki, K., Kajitani, N. et al. (2020). A non-mosaic transchromosomic mouse model of down syndrome carrying the long arm of human chromosome 21. *eLife* 9, e56223. 10.7554/eLife.56223 32597754 PMC7358007

[DMM049721C26] Leal, S. L. and Yassa, M. A. (2015). Neurocognitive Aging and the Hippocampus across Species. *Trends Neurosci.* 38, 800-812. 10.1016/j.tins.2015.10.003 26607684 PMC5218997

[DMM049721C27] Ling, K.-H., Hewitt, C. A., Tan, K.-L., Cheah, P.-S., Vidyadaran, S., Lai, M.-I., Lee, H.-C., Simpson, K., Hyde, L., Pritchard, M. A. et al. (2014). Functional transcriptome analysis of the postnatal brain of the Ts1Cje mouse model for Down syndrome reveals global disruption of interferon-related molecular networks. *BMC Genomics* 15, 624. 10.1186/1471-2164-15-624 25052193 PMC4124147

[DMM049721C28] Lorenzi, H., Duvall, N., Cherry, S., Reeves, R. and Roper, R. (2010). PCR prescreen for genotyping the Ts65Dn mouse model of Down syndrome. *BioTechniques* 48, 35-38. 10.2144/000113342 20095097 PMC2955870

[DMM049721C60] Love, M. I., Huber, W. and Anders, S. (2014). Moderated estimation of fold change and dispersion for RNA-seq data with DESeq2. *Genome Biol.* 15, 10.1186/s13059-014-0550-8 PMC4302049 25516281

[DMM049721C29] Luo, W., Friedman, M. S., Shedden, K., Hankenson, K. D. and Woolf, P. J. (2009). GAGE: generally applicable gene set enrichment for pathway analysis. *BMC Bioinformatics* 10, 161. 10.1186/1471-2105-10-161 19473525 PMC2696452

[DMM049721C63] Ma Y., Hof P. R., Grant S. C., Blackband S. J., Bennett R., Slatest L., McGuigan M. D. and Benveniste H. (2005). A three-dimensional digital atlas database of the adult C57BL/6J mouse brain by magnetic resonance microscopy. Neuroscience 135, 1203-1215. 10.1016/j.neuroscience.2005.07.014 16165303

[DMM049721C30] Marechal, D., Brault, V., Leon, A., Martin, D., Pereira, P. L., Loaëc, N., Birling, M.-C., Friocourt, G., Blondel, M. and Herault, Y. et al. (2019). Cbs overdosage is necessary and sufficient to induce cognitive phenotypes in mouse models of Down syndrome and interacts genetically with Dyrk1a. *Hum. Mol. Genet.* 28, 1561-1577. 10.1101/393579 30649339

[DMM049721C31] Martinez-Cue, C., Baamonde, C., Lumbreras, M., Paz, J., Davisson, M. T., Schmidt, C., Dierssen, M. and Flórez, J. (2002). Differential effects of environmental enrichment on behavior and learning of male and female Ts65Dn mice, a model for Down syndrome. *Behav. Brain Res.* 134, 185-200. 10.1016/S0166-4328(02)00026-8 12191805

[DMM049721C32] Matsumoto, S., Fujii, S., Sato, A., Ibuka, S., Kagawa, Y., Ishii, M. and Kikuchi, A. (2014). A combination of Wnt and growth factor signaling induces Arl4c expression to form epithelial tubular structures. *EMBO J.* 33, 702-718. 10.1002/embj.201386942 24562386 PMC4000088

[DMM049721C33] Moore, C. S., Hawkins, C., Franca, A., Lawler, A., Devenney, B., Das, I. and Reeves, R. H. (2010). Increased male reproductive success in Ts65Dn “Down syndrome” mice. *Mamm. Genome* 21, 543-549. 10.1007/s00335-010-9300-8 21110029 PMC3002156

[DMM049721C34] Muñiz Moreno, M. D. M., Brault, V., Birling, M.-C., Pavlovic, G. and Herault, Y. (2020). Modeling Down syndrome in animals from the early stage to the 4.0 models and next. *Prog. Brain Res.* 251, 91-143. 10.1016/bs.pbr.2019.08.001 32057313

[DMM049721C35] Nagamani, S. C., Erez, A., Eng, C., Ou, Z., Chinault, C., Workman, L., Coldwell, J., Stankiewicz, P., Patel, A., Lupski, J. R. et al. (2009). Interstitial deletion of 6q25.2-q25.3: a novel microdeletion syndrome associated with microcephaly, developmental delay, dysmorphic features and hearing loss. *Eur. J. Hum. Genet.* 17, 573-581. 10.1038/ejhg.2008.220 19034313 PMC2986272

[DMM049721C36] Nakano-Kobayashi, A., Awaya, T., Kii, I., Sumida, Y., Okuno, Y., Yoshida, S., Sumida, T., Inoue, H., Hosoya, T. and Hagiwara, M. (2017). Prenatal neurogenesis induction therapy normalizes brain structure and function in Down syndrome mice. *Proc. Natl. Acad. Sci. USA* 114, 10268-10273. 10.1073/pnas.1704143114 28874550 PMC5617268

[DMM049721C37] Neumann, F., Gourdain, S., Albac, C., Dekker, A. D., Bui, L. C., Dairou, J., Schmitz-Afonso, I., Hue, N., Rodrigues-Lima, F., Delabar, J. M. et al. (2018). DYRK1A inhibition and cognitive rescue in a Down syndrome mouse model are induced by new fluoro-DANDY derivatives. *Sci. Rep.* 8, 2859. 10.1038/s41598-018-20984-z 29434250 PMC5809559

[DMM049721C38] Nguyen, T. L., Duchon, A., Manousopoulou, A., Loaëc, N., Villiers, B., Pani, G., Karatas, M., Mechling, A. E., Harsan, L. A., Limanton, E. et al. (2018). Correction of cognitive deficits in mouse models of Down syndrome by a pharmacological inhibitor of DYRK1A. *Dis. Model. Mech.* 11, dmm035634. 10.1242/dmm.035634 30115750 PMC6176987

[DMM049721C39] O'Doherty, A., Ruf, S., Mulligan, C., Hildreth, V., Errington, M. L., Cooke, S., Sesay, A., Modino, S., Vanes, L., Hernandez, D. et al. (2005). An aneuploid mouse strain carrying human chromosome 21 with Down syndrome phenotypes. *Science* 309, 2033-2037. 10.1126/science.1114535 16179473 PMC1378183

[DMM049721C40] Olmos-Serrano, J. L., Kang, H. J., Tyler, W. A., Silbereis, J. C., Cheng, F., Zhu, Y., Pletikos, M., Jankovic-Rapan, L., Cramer, N. P., Galdzicki, Z. et al. (2016). Down syndrome developmental brain transcriptome reveals defective oligodendrocyte differentiation and myelination. *Neuron* 89, 1208-1222. 10.1016/j.neuron.2016.01.042 26924435 PMC4795969

[DMM049721C41] Pizzo, L., Jensen, M., Polyak, A., Rosenfeld, J. A., Mannik, K., Krishnan, A., McCready, E., Pichon, O., Le Caignec, C., Van Dijck, A. et al. (2019). Rare variants in the genetic background modulate cognitive and developmental phenotypes in individuals carrying disease-associated variants. *Genet. Med.* 21, 816-825. 10.1038/s41436-018-0266-3 30190612 PMC6405313

[DMM049721C42] Reeves, R. H., Irving, N. G., Moran, T. H., Wohn, A., Kitt, C., Sisodia, S. S., Schmidt, C., Bronson, R. T. and Davisson, M. T. (1995). A mouse model for Down syndrome exhibits learning and behaviour deficits. *Nat. Genet.* 11, 177-184. 10.1038/ng1095-177 7550346

[DMM049721C43] Richtsmeier, J., Baxter, L. and Reeves, R. (2000). Parallels of craniofacial maldevelopment in Down syndrome and Ts65Dn mice. *Dev. Dyn.* 217, 137-145. 10.1002/(SICI)1097-0177(200002)217:2<137::AID-DVDY1>3.0.CO;2-N 10706138

[DMM049721C44] Ruby, N., Fernandez, F., Zhang, P., Klima, J., Heller, H. and Garner, C. C. (2010). Circadian locomotor rhythms are normal in Ts65Dn “Down syndrome” mice and unaffected by pentylenetetrazole. *J. Biol. Rhythms* 25, 63-66. 10.1177/0748730409356202 20075302

[DMM049721C66] Sawiak, S. J., Wood, N. I., Williams, G. B., Morton, A. J. and Carpenter, T. A. (2009). SPMMouse: A new toolbox for SPM in animal brain. *Proc. Int. Soc. Mag. Res. Med.* 17, 1086.

[DMM049721C45] Schultz, H., Sommer, T. and Peters, J. (2015). The role of the human entorhinal cortex in a representational account of memory. *Front. Hum. Neurosci.* 9, 628. 10.3389/fnhum.2015.00628 26635581 PMC4653609

[DMM049721C46] Shaw, P. R., Klein, J. A., Aziz, N. M. and Haydar, T. F. (2020). Longitudinal neuroanatomical and behavioral analyses show phenotypic drift and variability in the Ts65Dn mouse model of Down syndrome. *Dis. Model. Mech.* 13, dmm046243. 10.1242/dmm.046243 32817053 PMC7522024

[DMM049721C47] Sierra, C., De Toma, I., Cascio, L. L., Vegas, E. and Dierssen, M. (2021). Social factors influence behavior in the novel object recognition task in a mouse model of Down syndrome. *Front. Behav. Neurosci.* 15, 772734. 10.3389/fnbeh.2021.772734 34803627 PMC8602686

[DMM049721C48] Starbuck, J. M., Dutka, T., Ratliff, T. S., Reeves, R. H. and Richtsmeier, J. T. (2014). Overlapping trisomies for human chromosome 21 orthologs produce similar effects on skull and brain morphology of Dp(16)1Yey and Ts65Dn mice. *Am. J. Med. Genet. A* 164A, 1981-1990. 10.1002/ajmg.a.36594 24788405 PMC4107150

[DMM049721C67] Tustison N. J., Avants B. B., Cook P. A., Zheng Y., Egan A., Yushkevich P. A. and Gee J. C. (2010). N4ITK: improved N3 bias correction. *IEEE Trans. Med. Imaging* 29, 1310-1320. 10.1109/TMI.2010.2046908 20378467 PMC3071855

[DMM049721C68] Vandesompele J., De Preter K., Pattyn F., Poppe B., Van Roy N., De Paepe A. and Speleman F. (2002). Accurate normalization of real-time quantitative RT-PCR data by geometric averaging of multiple internal control genes. *Genome Biol.* 3, research0034.1. 10.1186/gb-2002-3-7-research0034 12184808 PMC126239

[DMM049721C69] Vorhees C. V. and Williams M. T. (2006). Morris water maze: procedures for assessing spatial and related forms of learning and memory. *Nat. Protoc.* 1, 848-858. 10.1038/nprot.2006.116 17406317 PMC2895266

[DMM049721C70] Warfield S. K., Zou K. H. and Wells W. M. (2004). Simultaneous truth and performance level estimation (STAPLE): an algorithm for the validation of image segmentation. *IEEE Trans. Med. Imaging* 23, 903-921. 10.1109/TMI.2004.828354 15250643 PMC1283110

[DMM049721C49] Yonemura, S., Matsui, T., Tsukita, S. and Tsukita, S. (2002). Rho-dependent and -independent activation mechanisms of ezrin/radixin/moesin proteins: an essential role for polyphosphoinositides in vivo. *J. Cell Sci.* 115, 2569-2580. 10.1242/jcs.115.12.2569 12045227

