# Supplementary material for: Ts66Yah, a mouse model of Down syndrome with improved construct and face validity
Source: Dis Model Mech. 2022 Dec 6;15(12):dmm049721. doi: 10.1242/dmm.049721 (PMC9789398; doi:10.1242/dmm.049721)
Supplement: Supplementary information [file dmm-15-049721-s1.pdf]

A

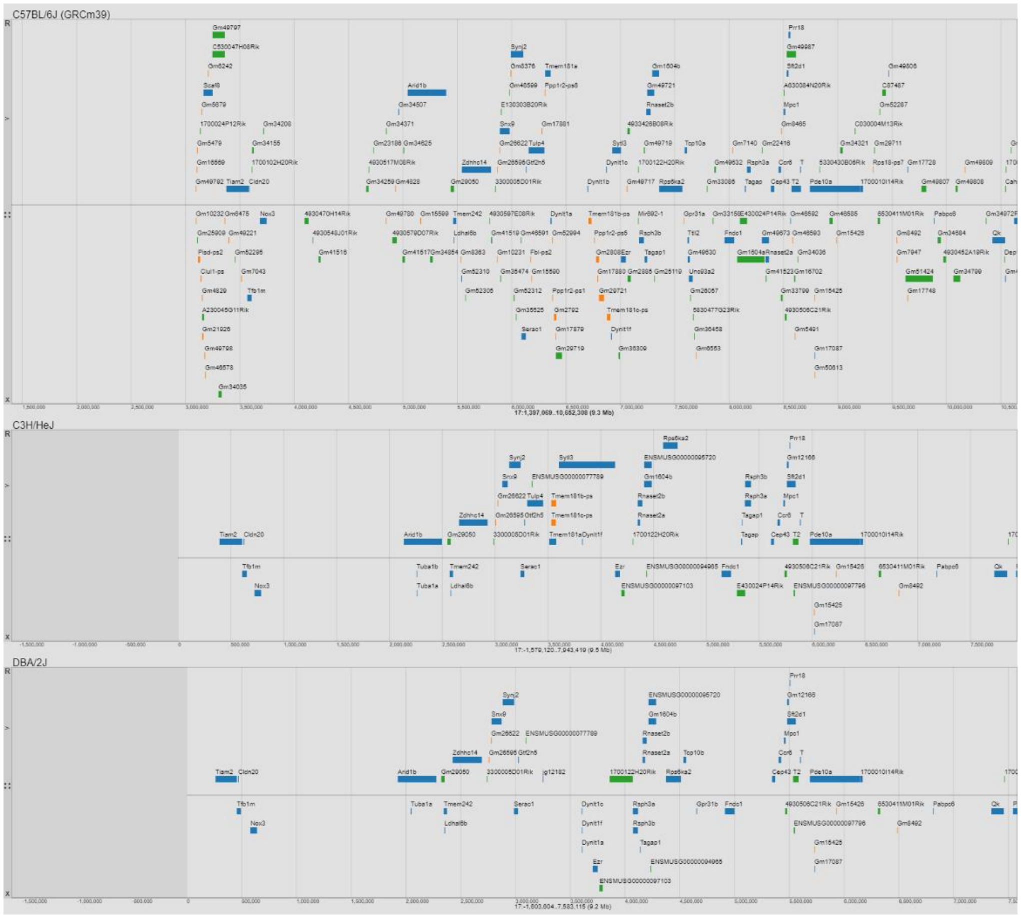

B

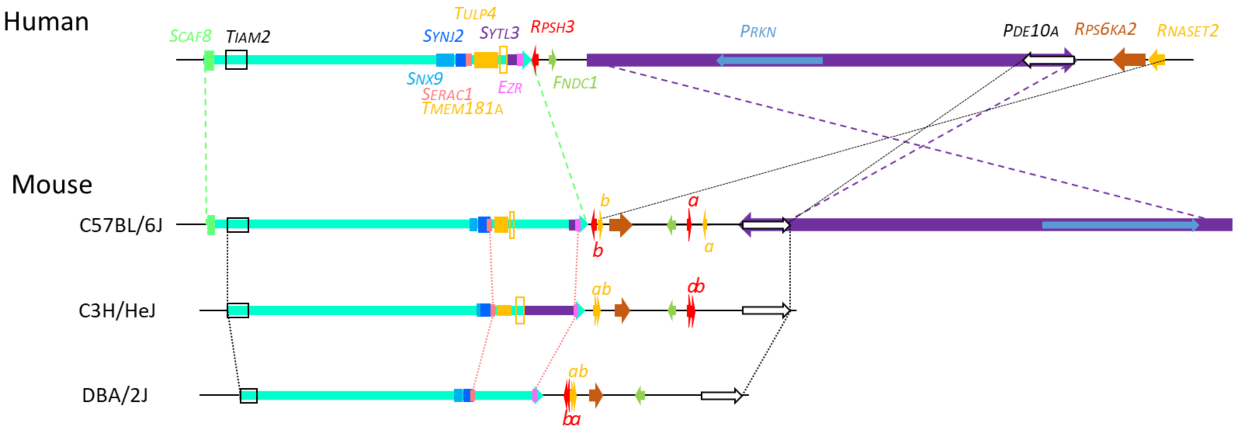

Disease Models & Mechanisms • Supplementary information

Fig. S1. The genomic organisation of the *Scaf8- Pde10a* region found on the centromeric part of the mouse chromosome 17 is conserved, but reorganized, in the C57BL/6J, C3H/HeJ and DBA/2J mouse strains, and showed several gene reorganization in the human genome. A. The copy was derived from the multiple genome viewer available at the Mouse genome informatics (www.informatics.jax.org/Mgv) including the region sequenced on C3H/HeJ::17:-1579120..7943419, on the C57BL/6::17:1397069..10652308 and DBA/2J::17:-1603604..7583115. with the GRCm39 reference genome. B. Schematic representation of the general organization of the *Scaf8-Pde10a* in the 3 mouse lines and in the human genome showing both preserved and altered loci.

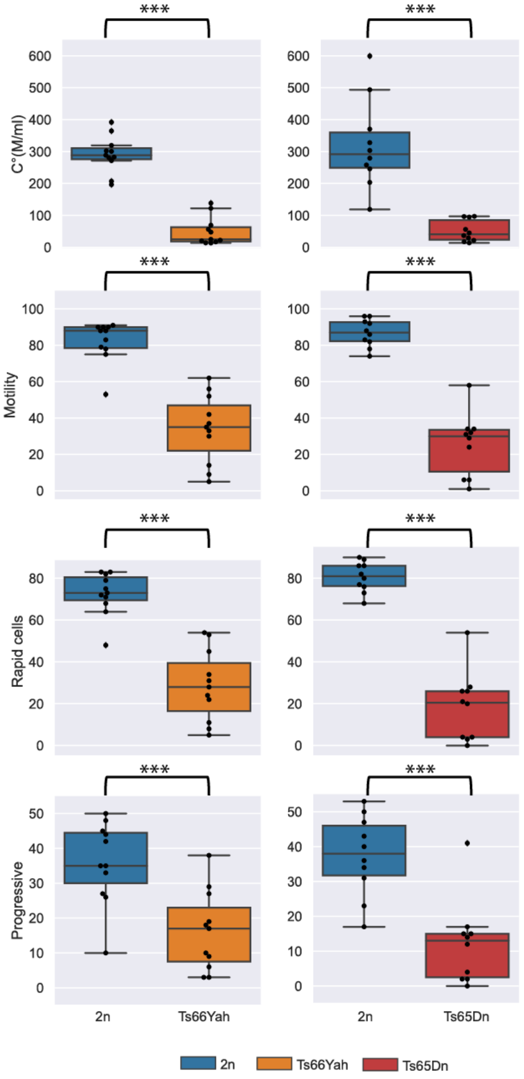

Fig. S2. Comparison of sperm analysis in the Ts66Yah and Ts65Dn DS models. Both models presented significant lower pattern of sperm quality in males as observed here with the spermatozoa concentration (C°, millions/ml), the percentage of motile cells (Motility %), the rapid cells (%) and the progressivity (%) in Ts66Yah (n = 11 for 2n control littermates and n = 11 for Ts animals; left panel) and Ts65Dn (n = 10 for 2n control littermates and n=10 for Ts animals; right panel) respectively. All parameters were significantly lower in trisomic mice. Box plots with the median and quartiles, ***$p<0.001$)

Disease Models & Mechanisms • Supplementary information

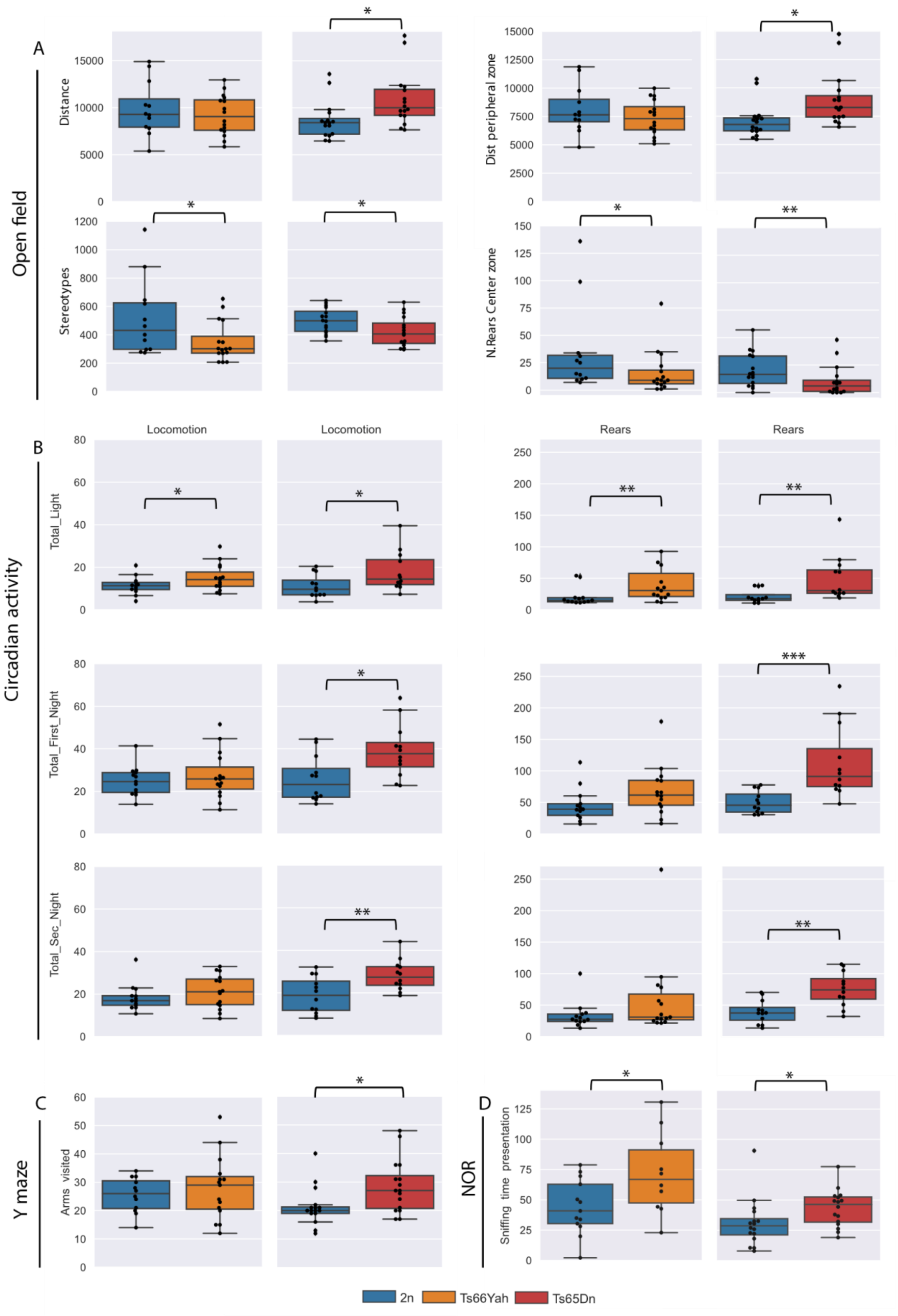

Disease Models & Mechanisms • Supplementary information

Fig. S3. Activities measured in different paradigms for the Ts66Yah and Ts65Dn males. A. In the open field, while Ts66Yah trisomic mice travelled the same distance as the 2n control mice, the Ts65Dn mice showed more activity in this paradigm. The number of stereotypes was increased in both trisomic mice. Only the Ts65Dn mice presented an increase in anxiety-like behaviour with a reduced activity in the centre and increased activity in the peripheral zone. The experiment was done with 2n (n = 12) and trisomic (Ts; n = 16) males for Ts66Yah and 2n (n = 16) and Ts (n = 16) males for Ts65Dn. B. left and right panels illustrate the locomotion and the number of rears respectively in the circadian activity during light, first and second night phases of the test. The locomotion pattern of Ts66Yah mice was similar to the 2n control mice regardless of test phases. On the contrary, Ts65Dn mice exhibited an increase in locomotion activity during light, first and second night phases. Similarly, rearing activity (right panel) was increased for the Ts65Dn trisomic mice during all phases of the test, whereas Ts66Yah trisomic mice showed the same activity level than the 2n control group of mice. This experiment was done with 2n (n = 12) and trisomic (Ts; n = 16) males for Ts66Yah and 2n (n = 12) and Ts (n = 12) males for Ts65Dn. C. Only the Ts65Dn mice showed hyperactivity in the Y maze test with increased arms entries compare to the 2n group of mice. This experiment was done with 2n (n = 12) and trisomic (Ts; n = 16) males for Ts66Yah and 2n (n = 16) and Ts (n = 16) males for Ts65Dn. D. in the novel object recognition test, both group of mice with trisomy (13 2n/10 Ts66Yah and 16 2n/16 Ts65Dn) presented an increased sniffing time during the presentation phase of the test. Data obtained in activity tests highlight a hyperactivity and an increased in anxiety-like behavior of Ts65Dn trisomic mice, observations that were not found in the Ts66Yah trisomic mice. Box plot representation with the median and quartiles, * $p<0.05$, ** $p<0.01$, *** $p<0.001$.

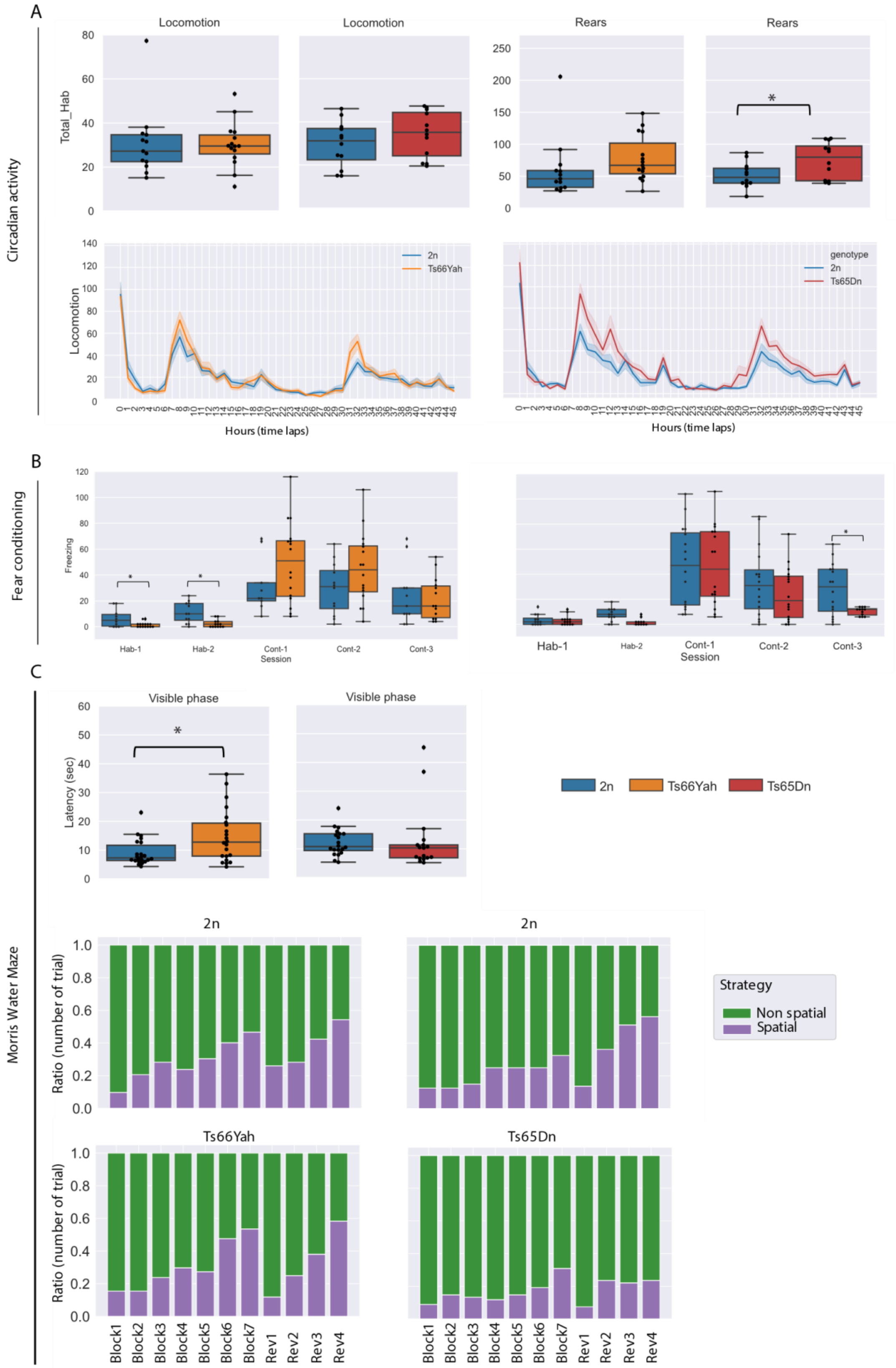
A
Circadian activity
Locomotion
Locomotion
Rears
Rears
Total_Hab
Locomotion
2n
Ts66Yah
genotype
2n
Ts65Dn
Hours (time laps)
Hours (time laps)
B
Fear conditioning
Freezing
Hab-1
Hab-2
Cont-1
Session
Cont-2
Cont-3
C
Morris Water Maze
Visible phase
Visible phase
Latency (sec)
2n
Ts66Yah
Ts65Dn
2n
2n
Ts66Yah
Ts65Dn
Ratio (number of trial)
Strategy
Non spatial
Spatial
Block1
Block2
Block3
Block4
Block5
Block6
Block7
Rev1
Rev2
Rev3
Rev4

Fig. S4. Investigating circadian activity, Morris water maze and fear conditioning comparing males from Ts66Yah and Ts65Dn DS models. A. upper panel illustrate the locomotion and number of rears respectively in the circadian activity during habituation phases of the test. There was no difference between the 2n control and trisomic mice for the locomotion pattern. Rearing activity was increased for the Ts65Dn trisomic mice only. Lower panel represent locomotion circadian activity for Ts66Yah and Ts65Dn mutants, both lines had a similar pattern than the control littermates. B. freezing time in seconds during the different phases of the FC test. All animals increased their freezing time after electrical shock. During the context session, 24 hours after the conditioning, all the mice showed a high level of freezing time, except for the Ts65Dn in the last minutes of the test (Cont-3), when mice recovered their activity at normal level. Ts66Yah, n = 12 for 2n and n = 16 for Ts individuals and Ts65Dn n = 16 for control and n = 16 for Ts individuals. C. upper panel represents latency taking by the mice to locate the PF during the MWM visible session. Lower panel represents the PF searching strategies undertaken by each mouse models along the test. Only the Ts65Dn mice showed a low and constant level of spatial navigation path compared to the control mice and the Ts66Yah line. This experiments were done with 2n (n = 22) and trisomic (Ts; n = 22) Ts66Yah males and 2n (n = 20) and Ts (n = 18) Ts65Dn males. Box plots with the median and quartiles, * $p<0.05$, ** $p<0.01$, *** $p<0.001$).

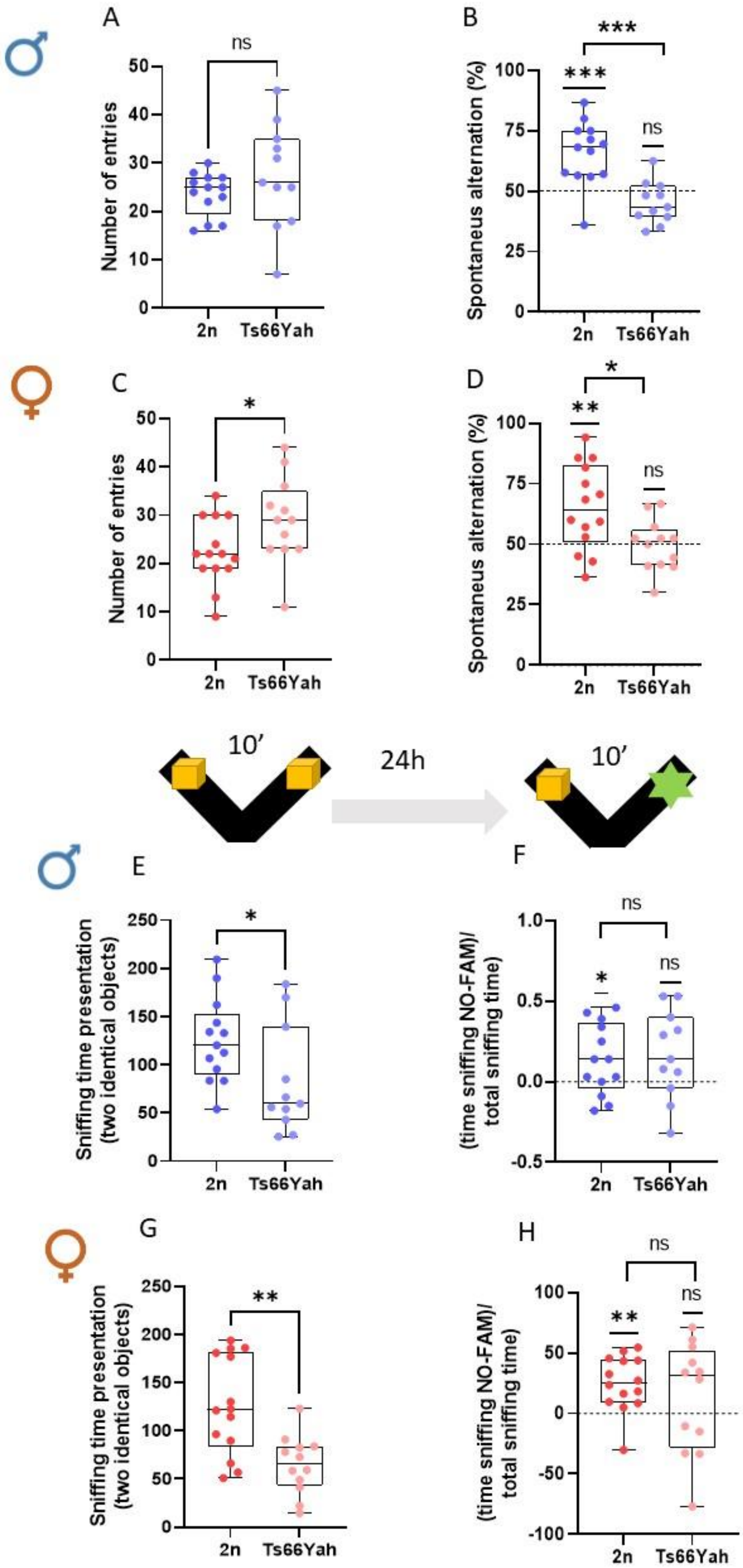

Disease Models & Mechanisms • Supplementary information

Fig. S5. Reproducibility of memory phenotypes affecting spontaneous alternation and object discrimination in both males and females from the Ts66Yah mouse line with no major sex effect.

During the Y maze test, the total number of entries (A-C) was not significantly different whereas the alternation rate (B-D) was lower in the Ts66Yah mice compared to control 2n littermates, and both sexes responded similarly. In the novel object recognition with 24-hour retention time, conducted in a V-maze (E-G), the total sniffing time of identical objects during the familiarization session was similar in males but not in females. During the test phase (F-H), the discrimination index showed no significant exploration of the novel object in both males and females Ts66Yah mice with no sex effect (ANOVA). One-sample t-test vs. 0% (hazard) showed that Ts66Yah male ($p = 0.07$) and female ($p = 0.341$) mice failed to recognize the new object. T-test for 2n vs. Ts66Yah comparisons. * $p<0.05$, ** $p<0.01$, *** $p<0.001$. Each dot represents an individual. Blue dots represent male mice, and red dots represent female mice; dark colors for 2n mice and light colors for Ts66Yah mice (Ts66Yah, Male, n = 13 for 2n and n = 11 for Ts; Female n = 14 for 2n and n = 12 for Ts). Box plots represent the median and quartiles).

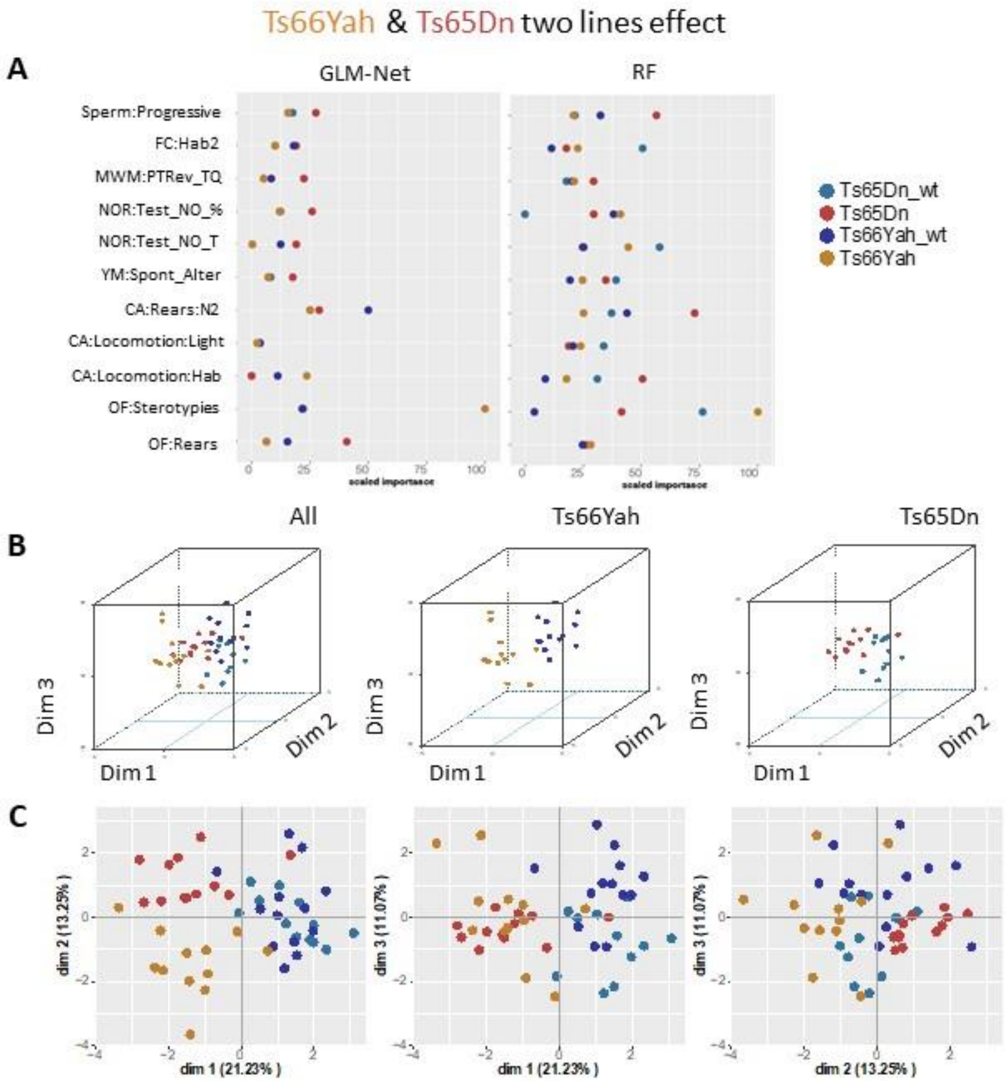

Fig. S6. Identification of discriminating phenotypic variables contributing the most to the genotype discrimination considering the two models Ts65Dn and Ts66Yah models together. A. Identification of the power of each explanatory phenotypic variable to the genotype discrimination. The explanatory variables selected were the ones known to contribute more than 30% to the genotype discrimination. Those variables were identified using a principal component analysis (PCA) after applying a multi-factor analysis of mixed data (MFA) implemented using MFAmix function from the PCAmixdata R package. The relevance of those variables to the genotype discrimination was analyzed using two different statistical classifiers: Lasso and Elastic-Net Regularized Generalized Linear Models, noted as GLM-Net taken from the caret R package, and Random forest, noted RF taken from the caret R package. All

measures of importance are scaled to have a maximum value of 100 in the variable contributing the most to the discrimination in the 2n control (wild type, wt) Ts66Yah. With GLM-Net and RF we can identify the importance of each variable to discriminate each genotype. B. 3D-PCA plots showing the individual animals clustering on the 3D space based on the PCA analyses performed with all the phenotypic variables and colored based on genotype and model as follows, in soft blue Ts65Dn control (wt), in red Ts65Dn DS trisomic, in dark blue the Ts66Yah wild-types (wt) and in medallion yellow Ts66Yah DS mutants. The upper panel shows all individuals from the two models, mixed genotypes, the middle plot shows only the Ts66Yah model and the bottom plot only the Ts65Dn. **C.** Individual component map. The distribution in 2D space of the individual observation coordinates calculated based on the PCA analysis performed after the MFA implemented using MFAmix function from the PCAmixdata R package.

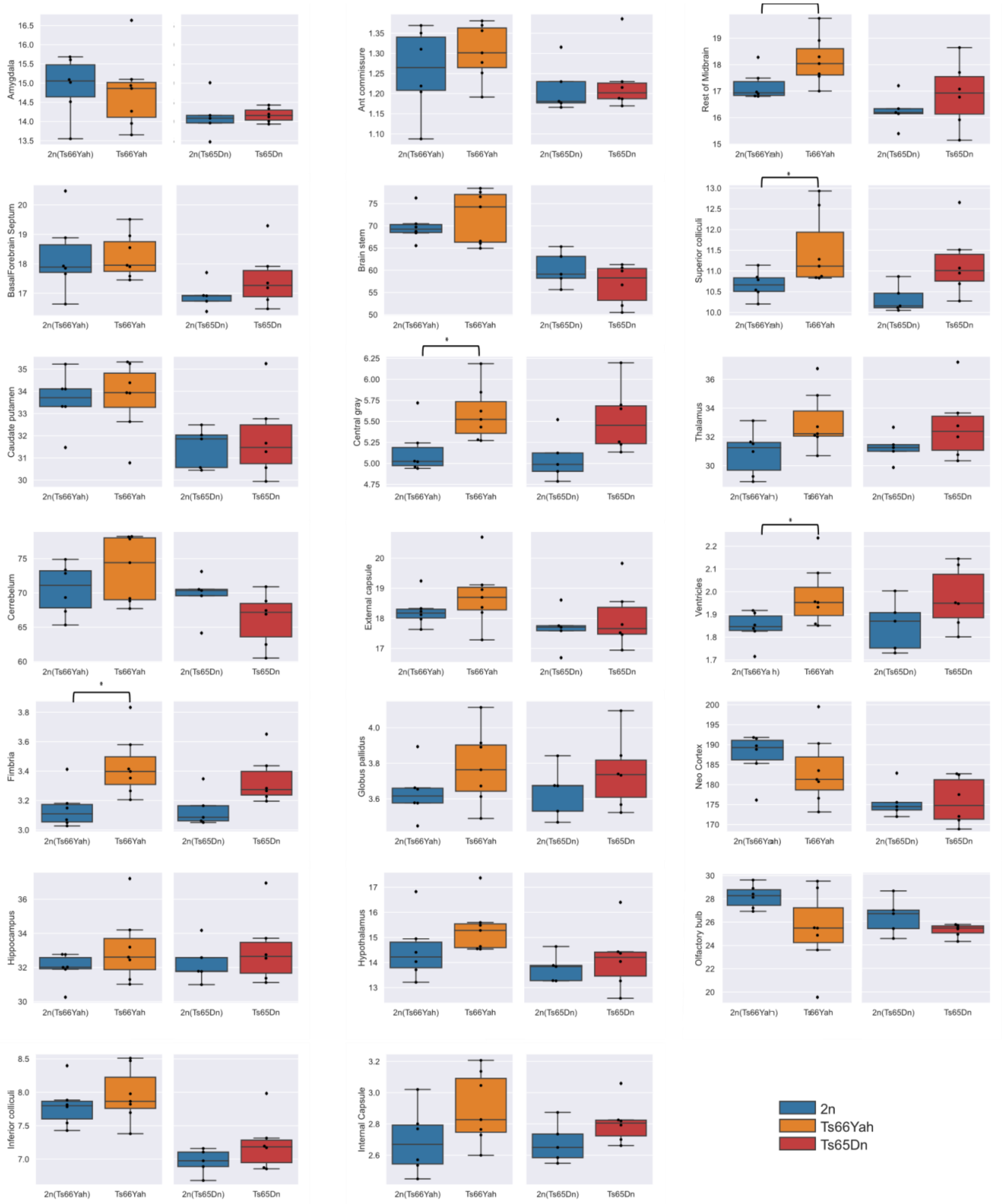

Fig. S7. Changes observed by brain structures using MRI. The changes in the morphology of specific brain structures and the direction of the changes (increase or decrease of volume) were the same in both DS lines. However, the amplitude of the changes was less severe in the Ts66Yah than in the Ts65Dn mice. Central grey, olfactory bulb or thalamus were not affected in the Ts66Yah mutants compared to the controls. Ts66Yah, n = 6 for 2n and n = 7 for Ts individuals and Ts65Dn n = 5 for control and n = 6 for Ts individuals. Box plots represent the median and quartiles, * p<0.05, **p<0.01, ***p<0.001.

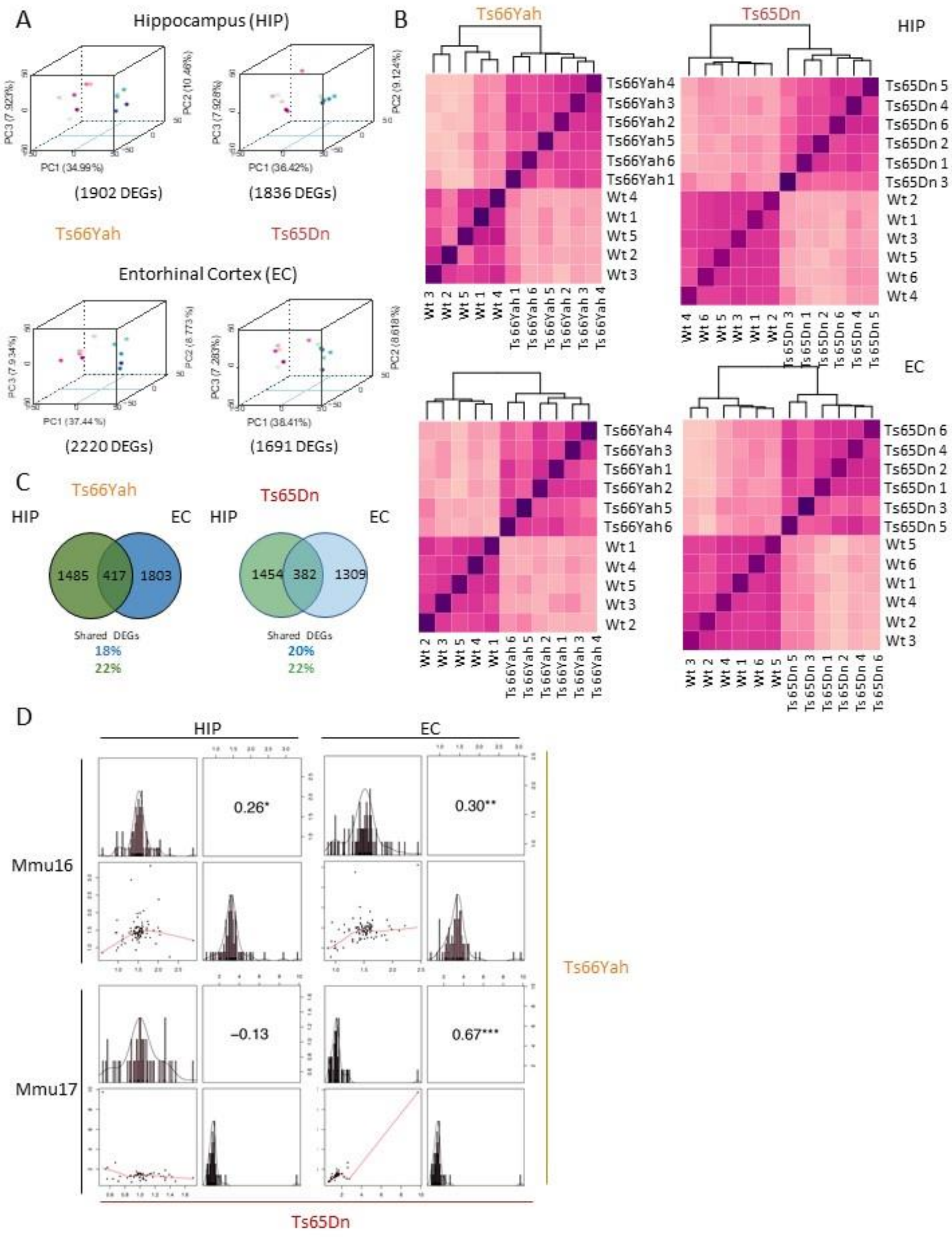

Disease Models & Mechanisms • Supplementary information

Fig. S8. Gene expression analysis in the HIP and EC of Ts66Yah and Ts65Dn mice. A. 3D-PCA on the DEGs for each adult hippocampal sample or entorhinal cortex sample allows to separate the animals carrying the Ts65Dn or Ts66Yah trisomies (pink) in comparison with the wild-type littermates (wt, blue). B. Homogenicity plot showing on the left how the Ts66Yah samples and the wt littermates cluster by Euclidean distance, on the right how the Ts65Dn samples and wt littermates cluster by Euclidian distance. C. Venn diagram showing the DEGs in common between Ts66Yah entorhinal cortex and hippocampi samples in the upper panel, and between Ts65Dn entorhinal cortex and hippocampi samples on the bottom panel. The shared DEGs correspond to 18% and 22% of the total DEGs identified respectively for the Ts66Yah entorhinal cortex and hippocampi samples; and 20% and 22 % for the Ts65Dn entorhinal cortex and hippocampi samples. In the intersection between the Venn diagrams circles are highlighted in the middle in bold the common genes regulated in mirroring sense, instead the common DEGs deregulated following the same regulatory sense specified in the upper (upregulated shared DEGs) and bottom (for the downregulated shared DEGs) part of the intersection between the Venn diagrams circles. D. Spearman correlation between Ts65Dn and Ts66Yah models based on TEGs identified in the Hippocampus (Hip) or the enthorinal cortices (EC) located in the Mmu16 homologous to Hsa21 or in the Mmu17 centromeric region.

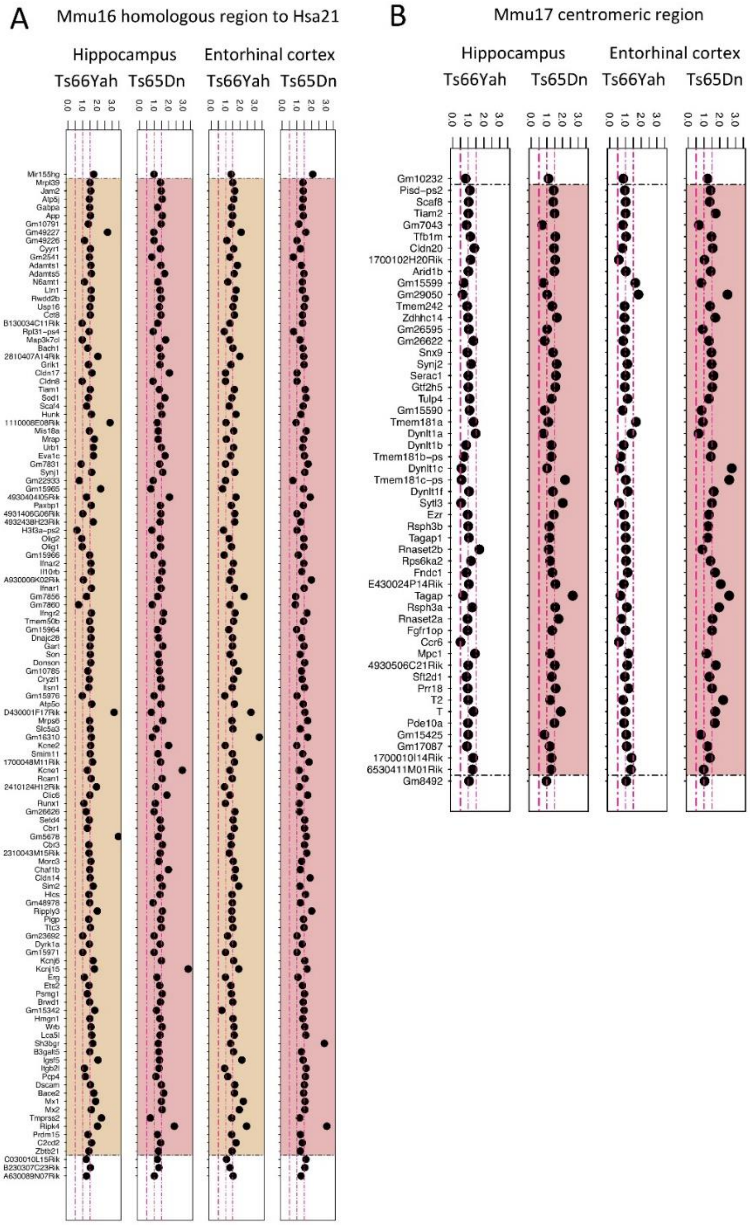

Fig. S9. Expression of genes found on mouse chromosome 16 homologous region to Hsa21 (A) and on the 17 centromeric region (B) in Ts65Dn and Ts66Yah models. **A**. The duplicated regions on Mmu16 found in the Ts65Dn and Ts66Yah models are highlighted in red or orange respectively whereas in **B**, the duplicated region on Mmu17 for the Ts65Dn model is highlighted in red and also for the Ts66Yah (no color). The genes are displayed following the order of their genomic start site coordinates from top to bottom.

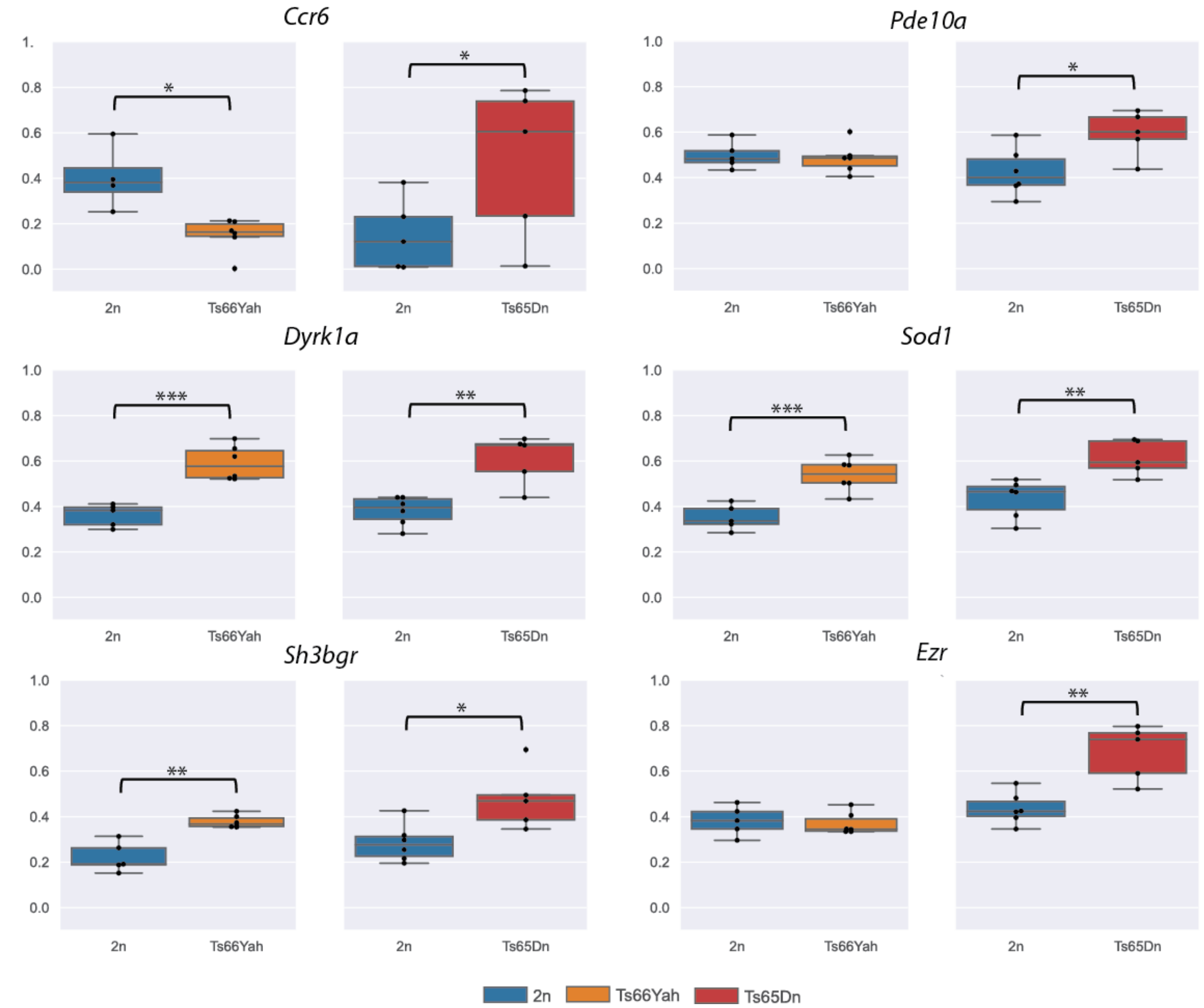

Fig. S10. Quantification of the relative expression of selected messenger RNA isolated from the hippocampi of the Ts65Dn and Ts66Yah mouse models.

Quantitative gene expression in trisomic mice compared to controls (2n: n=5; Ts66Yah n=6; 2n: n=6; Ts65Dn n=5) is reported here as boxplot. *Ccr6* was down-regulated in Ts66Yah and up-regulated in Ts65Dn. *Pde10a* was only upregulated in Ts65Dn, because is located in the Mmu17 deleted region. *Dyrk1a* and *Sod1* genes located in triplicated Mmu16 region were up regulated in both models. *Sh3bgr* and *Ezr* non-triplicated genes were dysregulated in different ways depending of the trisomic models (Box plots represent the median and quartiles, * $p<0.05$, ** $p<0.01$, *** $p<0.001$).

Disease Models & Mechanisms • Supplementary information

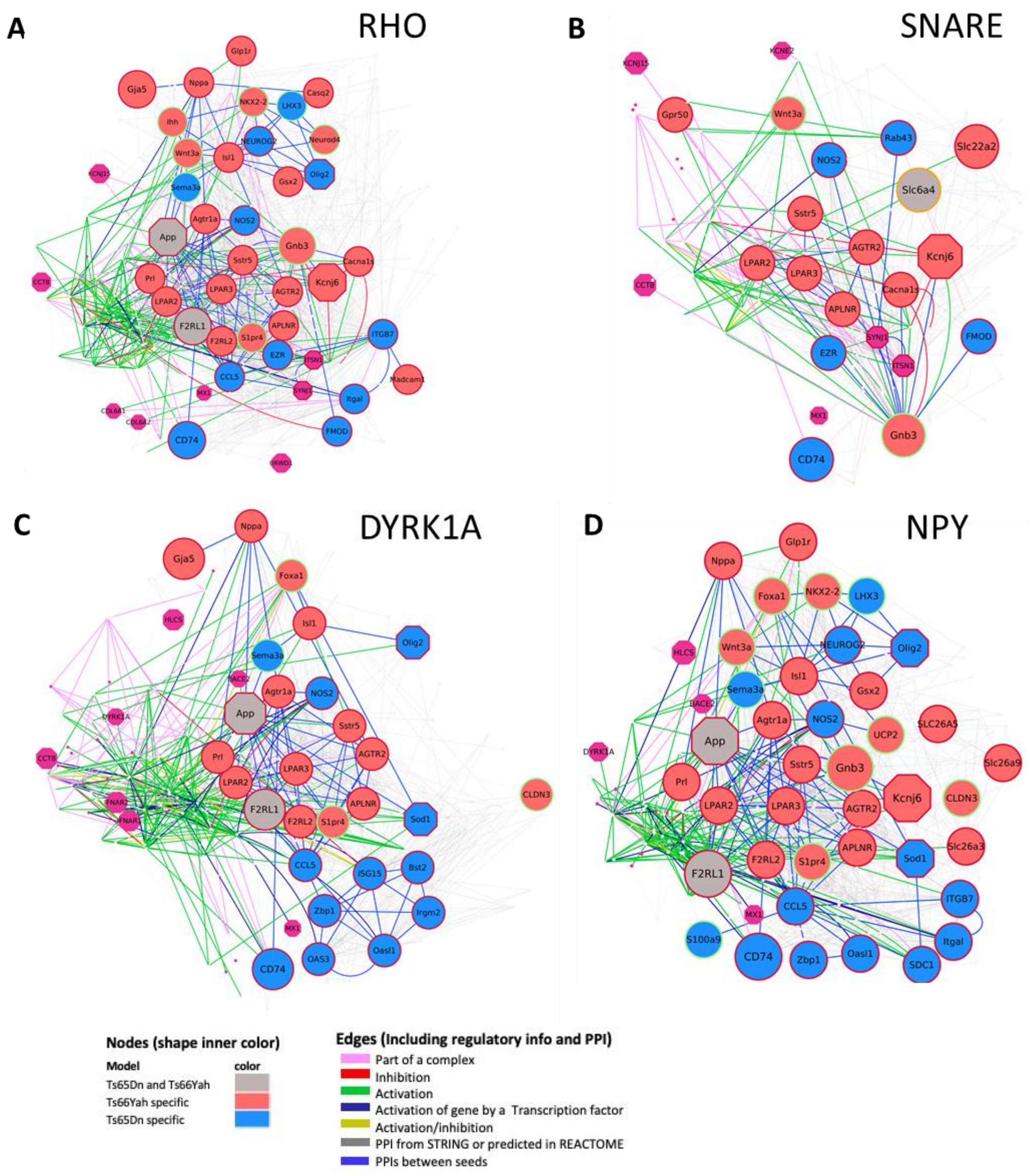

Fig. S11. Ts65Dn and Ts66Yah Protein-protein interaction and regulatory gene connectivity network (RegPPINets) of genes involved in the synaptic meta-pathway identified in Ts65Dn or Ts66Yah highlighting the main biological cascades with altered connectivity. These results in Ts66Yah and Ts65Dn support the role of the DS subnetworks centered on RHOA, SNARE proteins, NPY and DYRK1A cascades. A. Highlighting RHOA/RHOC/RHO 2nd interactors. B. Highlighting SNARE VAMPS/SEC proteins 2nd interactors. C. Highlighting DYRK1A 2nd interactors. D. Highlighting NPY 2nd interactors.

Disease Models & Mechanisms • Supplementary information

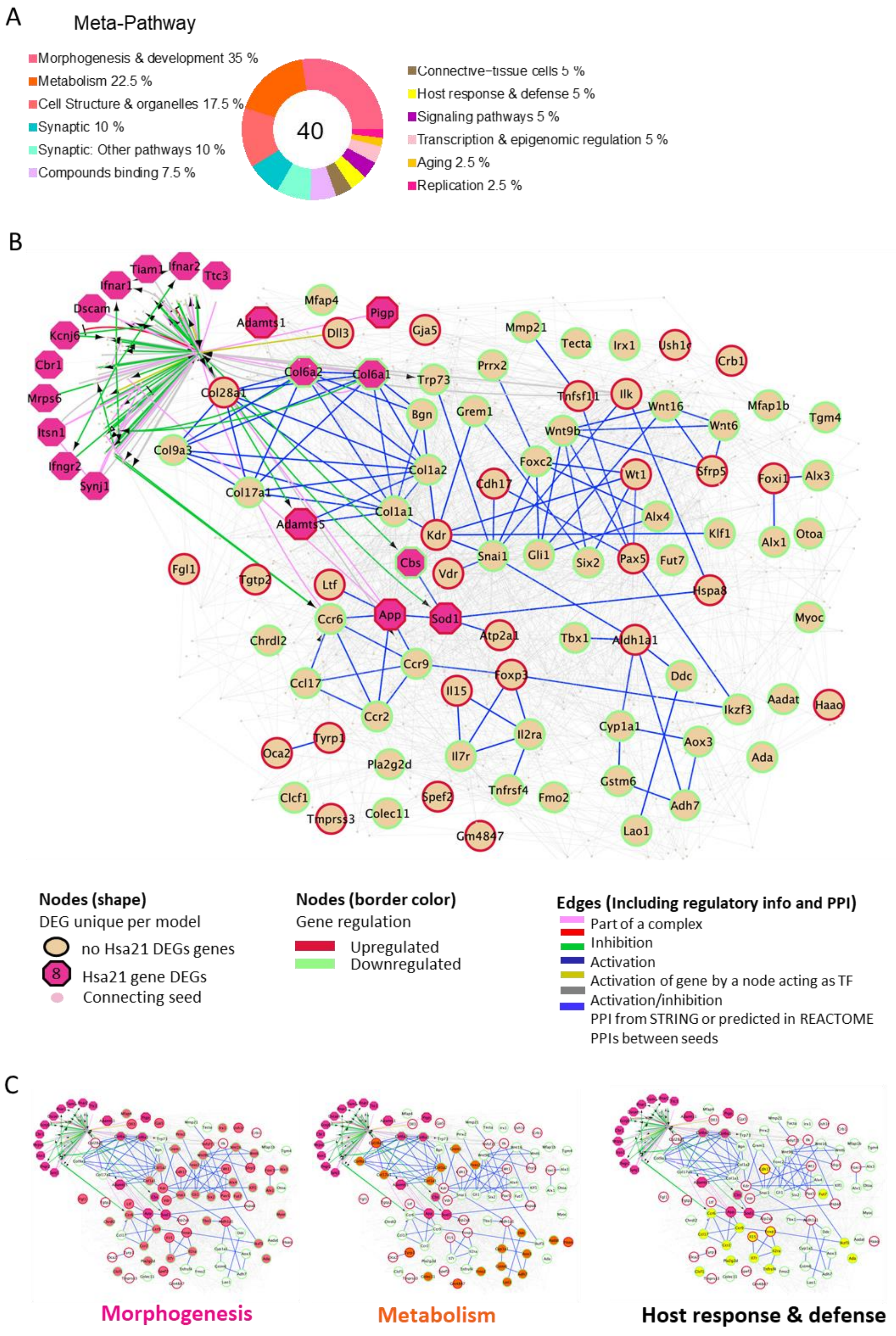

Disease Models & Mechanisms • Supplementary information

Fig. S12. Ts66Yah entorhinal cortex functional Network analysis. A. Donut plot representing the functional alterations grouped per meta-pathway in the Ts66Yah entorhinal cortex (noted as EC). In the centre of the donut plot the number of total altered pathways identified by gage; at left and right a summary showing the percentage of pathways included on each of the more populated meta-pathways. B. Full Ts66Yah entorhinal cortex RegPPINet network built using as seeds all the altered genes identified by gage in the Ts66Yah entorhinal cortex model visualized using the edge weighed spring embedded layout with the nodes ordered by betweenness index in Cytoscape. The full RegPPINet was built by querying STRING and selecting the PPIs with a medium confidence score (CS=0.4) coming from all sources of evidence. The shapes of the nodes and the inner colour represents the following information: Mustard circles: DEGs found altered in the model; Magenta octagons: represent HSA21 syntenic genes in mouse, some identified as contributing to those meta-pathway dysregulations by GAGE; Pallid pink Ellipses: In smaller size than the rest of the node shapes, represent connecting proteins added in the network to not have unconnected nodes assure the full connectivity of the network. The edge colour represents the type of interaction annotated by the PathPPI classification (TANG *et al.* 2015), and the ReactomeFIViz annotations as follows i) The GErel edges indicating expression were colored in blue and repression in yellow. ii) PPrel edges indicating activation were coloured in green, inhibition in red. Iii) Interactions between proteins known to be part of complexes in violet. Iv) Predicted interactions were represented in grey including the PPI interactions identified by STRING DB (SZKLARCZYK *et al.* 2017) after merging both networks. C. Ts66Yah entorhinal cortex RegPPINet network colored highlighting genes known to be involved in the following comorbidities, Host response & Immune system (yellow), genes involve in metabolism (orange), morphogenesis (magenta pink).

Disease Models & Mechanisms • Supplementary information

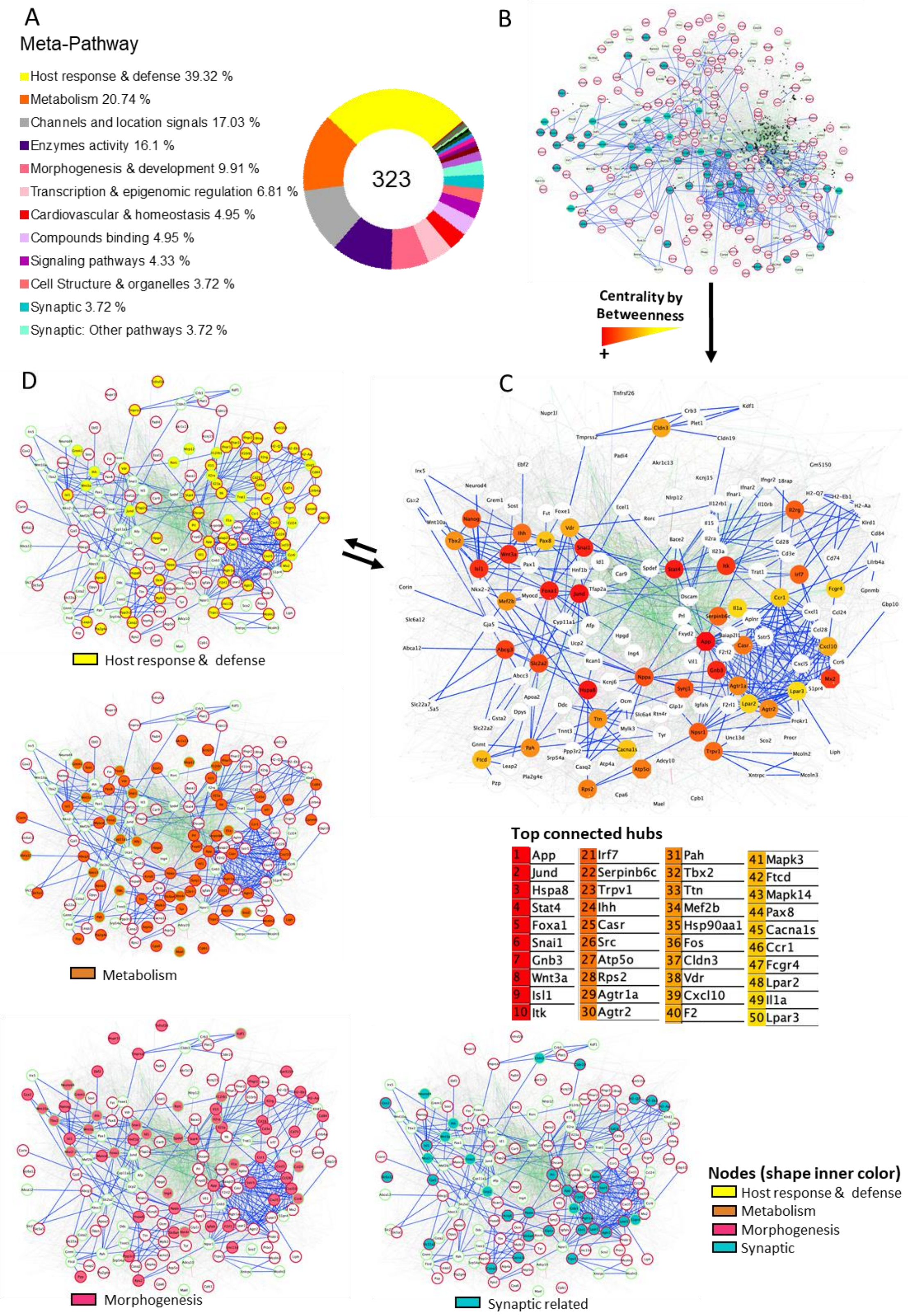
A
Meta-Pathway
Host response & defense 39.32 %
Metabolism 20.74 %
Channels and location signals 17.03 %
Enzymes activity 16.1 %
Morphogenesis & development 9.91 %
Transcription & epigenomic regulation 6.81 %
Cardiovascular & homeostasis 4.95 %
Compounds binding 4.95 %
Signaling pathways 4.33 %
Cell Structure & organelles 3.72 %
Synaptic 3.72 %
Synaptic: Other pathways 3.72 %
323
B
Centrality by Betweenness
+
C
D
Host response & defense
Metabolism
Morphogenesis
Synaptic related
Top connected hubs
1 App
2 Jund
3 Hspa8
4 Stat4
5 Foxa1
6 Snai1
7 Gnb3
8 Wnt3a
9 Isl1
10 Itk
21 Irf7
22 Serpinb6c
23 Trpv1
24 Ihh
25 Casr
26 Src
27 Atp5o
28 Rps2
29 Agtr1a
30 Agtr2
31 Pah
32 Tbx2
33 Ttn
34 Mef2b
35 Hsp90aa1
36 Fos
37 Cldn3
38 Vdr
39 Cxcl10
40 F2
41 Mapk3
42 Ftcd
43 Mapk14
44 Pax8
45 Cacna1s
46 Ccr1
47 Fcgr4
48 Lpar2
49 Il1a
50 Lpar3
Nodes (shape inner color)
Host response & defense
Metabolism
Morphogenesis
Synaptic

Fig. S13. Ts66Yah Hippocampi functional Network analysis. A, Donut plot representing the functional alteration per meta-pathway. In the centre the number of total altered pathways of the Ts66Yah Hippocampi and then the summary in percentages of the more populated meta-pathways. On the right, specifying the total number of pathways included on the synaptic meta-pathway**.** B, The full Ts66Yah Hippocampi RegPPINet network was built using as seeds all the genes identified by gage as altered in the Ts66Yah Hippocampi model visualized using the edge weighed spring embedded layout by betweenness index in Cytoscape. The full RegPPINet was built by querying STRING and selecting the PPIs with a medium confidence score (CS=0.4) coming from all sources of evidence. The shapes of the nodes represent the following information: Shapes: i) Pallid small pink ellipses: represent connecting proteins added to assure the full connectivity of the network; ii) Rest of the elliptic nodes represent genes identified by GAGE after q-val <0.1 cut off to be contributing even slightly, to any pathway of those found dysregulated inside the meta-pathway. Ii) Octagons represent the mouse Hsa21 syntenic genes. The edge colour represents the type of interaction annotated by following the PathPPI classification (Tang et al. 2015), and the ReactomeFIViz annotations as follows i) The GErel edges indicating expression were coloured in blue and repression in yellow. ii) PPrel edges indicating activation were coloured in green, inhibition in red. Iii) Interactions between proteins known to be part of complexes in violet. Iv) Predicted interactions were represented in grey including the PPI interactions identified by STRING DB (Szklarczyk et al. 2017) after merging both networks. * Represents those genes from the top 50 central genes list on the right side, known to be involved in synaptic pathways (they were linked to those functionalities in the GO/KEGG databases). C, Central Ts66Yah Hippocampi RegPPINet network colored i) in the upper panel highlighting the top 50 genes with the highest betweenness index value from red (higher index) to yellow (lower index) ii) in the lower panel highlighting the genes identified by gage to contribute to the alteration of synaptic related pathways. The list of the most central genes is included below the figure. D, Ts66Yah Hippocampi RegPPINet network colored highlighting genes known to be involved in comorbidities linked to morphogenesis, metabolism, and immune system highlighted in pink, orange and yellow on each network respectively.

Disease Models & Mechanisms • Supplementary information

Table S1. Detailed behaviour statistics results (statistically significant results are highlighted).

First environment	Ts66Yah	Ts65Dn
**Sperm Analysis**		
Number of individuals	11 2n/11 Ts	10 2n/10 Ts
Sexe	Male	Male
Nb of litters	8	7
Concentration(M/ml)	*t* (20) = 11.056 ; *p*<0.001	*H*(1) = 14.286 ; *p*<0.001
Motility %	*t* (20) = 7.287 ; *p*<0.001	*t*(18) = 10.338 ; *p*<0.001
Rapid cells %	*t* (20) = 7.323 ; *p*<0.001	*t*(18) = 10.857 ; *p*<0.001
Progressive %	*t* (20) = 3.964 ; *p*<0.001	*t*(18) = 4.776 ; *p*<0.001
**Nesting**		
Sexe	Male	Male
Nb of litters	15	7
Number of individuals	22 2n/25 Ts	15 2n/12 Ts
Nesting score	*H*(1) = 12.006 ; *p*<0.001	*H*(1) = 6.778 ; *p* = 0.012
**Square Open Field**		
Sexe	Male	Male
Nb of litters	10	11
Number of individuals	12 2n/16 Ts	16 2n/16 Ts
Distance total	*t* (26) = 0.713 ; *p* = 0.482	*H*(1) = 6.763 ; *p* = 0.009
Stereotypies	*H*(1) = 3.450; *p* = 0.063	*t*(30) = 2.249 ; *p*= 0.032
Velocity	*t* (26) = 0.708 ; *p*= 0.485	*H*(1) = 6.767 ; *p* = 0.009
Nb of rears	*H*(1) = 1.401 ; *p* = 0.236	*t*(30) = 0.348 ; *p*= 0.731
Nb of rears in center	*H*(1) = 4.180 ; *p* = 0.04	*H*(1) = 4.63 ; *p* = 0.03
Distance in peripheral zone	*t* (26) = 1.063 ; *p* = 0.297	*H*(1) = 8.865 ; *p* = 0.003
**Circadian Activity**		
Sexe	Male	Male
Nb of litters	10	8
Number of individuals	12 2n/16 Ts	12 2n/12 ts
Total Habituation Locomotion	*H*(1) = 0.0344 ; *p* = 0.852	*t*(22) = -1.072 ; *p* = 0.295
Total Habituation Rear	*H*(1) = 1.346 ; *p* = 0.245	*t*(22) = -2.309 ; *p* = 0.0307
Total Light Locomotion	*t* (26) = 5.175 ; *p* = 0.022	H(1) = 4.815 ; *p* = 0.028
Total Light Rear	*H*(1) = 9.108 ; *p* = 0.002	*H*(1) = 8.003 ; *p* = 0.005
Total First Night Locomotion	*t* (26) = 0.036 ; *p* = 0.971	*t*(22) = -2.761 ; *p* = 0.0114
Total First Night Rear	*H*(1) =0.551 ; *p* = 0.457	*H*(1) = 11.603 ; *p*<0.001
Total second Night Locomotion	*t* (26) =-0.757 ; *p* = 0.455	*t*(22) = -2.872 ; *p* = 0.0089
Total second Night Rear	*H*(1) =0.422 ; *p* = 0.515	*t*(22) = -3.783 ; *p* = 0.00102
**Y maze**		
Sexe	Male	Male
Nb of litters	10	11
Number of individuals	12 2n/16 Ts	16 2n/16 Ts

Arms visited within genotype	$t(25) = -0.671$; $p = 0.508$	$H(1) = 5.247$; $p = 0.022$
% spontaneous alternation within genotype	$t(25) = 1.88$; $p = 0.827$	$t(30) = 3.2$; $p = 0.003$
One sample t test spontaneous alternation vs 50% 2n	$t(11) = 4.266$; $p = 0.00133$	$t(15) = 5.371$; $p<0.001$
One sample t test spontaneous alternation vs 50% Ts	$t(11) = 0.117$; $p = 0.117$	$t(15) = 2.623$; $p = 0.019$
**NOR 24H**		
Sexe	Male	Male
Nb of litters	10	11
Number of individuals	13 2n/10 Ts	16 2n/16 Ts
One sample t test %NO vs 50% 2n	$t(12) = 2.744$; $p = 0.0178$	$t(15) = 2.211$; $p = 0.043$
One sample t test %NO vs 50% Ts	$t(9) = 1.056$; $p = 0.318$	$t(15) = 1.155$; $p = 0.266$
Object within genotype in habituation	$t(21) = -2.358$; $p = 0.0281$	$H(1) = 5.460$, $p = 0.019$
**Morris Water Maze**		
Sexe	Male	Male
Nb of litters	16	16
Number of individuals	22 2n/22 Ts	20 2n/18 Ts
**Hidden version**		
ANOVA RM Latency within genotype	$F(1,252) = 6.066$, $p = 0.018$	$F(1,210) = 10.841$, $p = 0.002$
ANOVA RM Velocity within genotype	$F(1,252) = 2.595$, $p = 0.115$	$F(1,210) = 0.198$, $p = 0.659$
ANOVA RM thigmotaxis within genotype	$F(1,252) = 1.011$, $p = 0.321$	$F(1,162) = 3.607$, $p = 0.068$
One sample t test %TQ vs 50% 2n	$t(21) = 5.966$; $p<0.001$	$t(21) = 3.085$; $p = 0.00561$
One sample t test %TQ vs 50% Tg	$t(21) = 2.852$; $p = 0.009$	$t(17) = 0.341$; $p = 0.737$
**Reversal**		
ANOVA RM Latency within genotype	$F(1,126) = 3.923$, $p = 0.054$	$F(1,105) = 16.361$, $p<0.001$
ANOVA RM Velocity within genotype	$F(1,126) = 0.0253$ $p = 0.874$	$F(1,105) = 0.000143$, $p<0.991$
ANOVA RM thigmotaxis within genotype	$F(1,126) = 0.358$ $p = 0.553$	$F(1,105) = 3.918$, $p = 0.056$
One sample t test %TQ vs 50% 2n	$t(21) = 2.871$; $p<0.009$	$t(20) = 2.388$; $p = 0.00269$
One sample t test %TQ vs 50% Ts	$t(21) = 2.796$; $p<0.011$	$t(17) = 0.847$; $p = 0.409$
**Cue version**		
ANOVA Latency within genotype	$H(1) = 4.414$, $p= 0.036$	$H(1) = 0.728$, $p= 0.393$
**Fear Conditioning**		
Sexe	Male	Male
Nb of litters	10	11
Number of individuals	12 2n/16 Ts	16 2n /16 Ts
**FC Hab1**		
Hab1 session	$H(1) = 3.95$; $p = 0.047$	$H(1) = 0.347$; $p = 0.852$
Hab2 session	$H(1) = 4.078$; $p = 0.043$	$t(22) = 3.264$; $p=0.00356$
**FC Context**		
Cont1	$t(26) = 0.006$; $p = 0.995$	$H(1) = 0.0128$; $p = 0.910$
Cont2	$t(26) = -1.74$; $p = 0.093$	$t(30) = 1.056$; $p = 0.299$
Cont3	$H(1) = 0.026$; $p = 0.870$	$H(1) = 4.031$; $p = 0.045$
**IRM analysis**		
Sexe	Male	Male
Nb of litters	5	3

number of individuals	6 2n/7 Ts	5 2n/6 Ts
Amygdala	*t*(11) = 0.277; *p*= 0.786	*t*(9) =-0.129; *p*=0.899
Ant commissure	*t*(11) = -0.959; *p*= 0.357	*H*(1) = 0.410; *p*= 0.521
BasalForebrain Septum	*t*(11) = -0.05; *p*= 0.955	*t*(9) = -1.145; *p*= 0.281
Brain stem	*t*(11) = -0.785 ; *p*= 0.448	*t*(9) = 1.325; *p*= 0.217
Caudate putamen	*t*(11) = -0.193 ; *p*= 0.850	*t*(9) = -0.463; *p*= 0.654
Central gray	*H*(1) = 5.22 ; *p* = 0.022	*t*(9) = -2.151; *p*= 0.059
Cerrebelum	*t*(11) = -1.22; *p*= 0.245	*t*(9) = 1.522; *p*= 0.162
External capsule	*t*(11) = -1.058; *p*= 0.312	*t*(9) = -0.643; *p*= 0.535
Fimbria	*t*(11) = -2.81; *p*= 0.016	*t*(9) = -2.18; *p*= 0.056
Globus pallidus	*t*(11) = -1.40 ; *p*= 0.187	*t*(9) = -1.021; *p*= 0.333
Hippocampus	*t*(11) = -1.27 ; *p*= 0.227	*t*(9) =- -0.757; *p*= 0.467
Hypothalamus	*H*(1) = -1.313 ; *p = 0.215*	*t*(9) = -0.633; *p*= 0.542
Inferior colliculi	*t*(11) = -0.72 ; *p*= 0.484	*t*(9) = -1.341; *p*= 0.212
Internal Capsule	*t*(11) = -1.712 ; *p*= 0.114	*t*(9) = -1.60; *p*= 0.142
Rest of Midbrain	*H*(1) = 5.224; *p*= 0.022	*t*(9) = -1.00 ; *p*= 0.341
Superior colliculi	*H*(1) = 4.59; *p*= 0.032	*t*(9) = -2.173; *p*= 0.057
Thalamus	*t*(11) = -2.077 ; *p* = 0.062	*t*(9) = -1.287; *p*= 0.230
Ventricles	*t*(11) = -2.222; *p* = 0.048	*t*(9) = -1.546; *p*= 0.156
WholeBrain	*t*(11) = -0.531 ; *p* = 0.605	*t*(9) = 0.089; *p*= 0.930
**qPCR analysis**		
Sexe	Male	Male
Nb of litters	5	7
number of individuals	5 2n/6 Ts	6 2n/5 Ts
Dyrk1a	*t*(9) = -6.033; *p* = 0.000236	*t*(9) =- 4.0958; *p* = 0.00594
Pde10A	*t*(9) = 0.316; *p* = 0.759	*t*(9) = -2.729; *p* = 0.0238
Sod1	*t*(9) = -4.892; *p* = 0.00086	*t*(9) = -3.675; *p* = 0.00522
Stat4	*t*(9) = -0.0297; *p* = 0.977	*H*(1) = 0.033 ; *p = 0.855*
Ccr6	*t*(9) = 3.267; *p* = 0.029	*t*(9) = -2.729; *p* = 0.0238
Ezr	*H*(1) = 0.3 ; *p = 0.584*	*t*(9) = -4.058; *p* = 0.00627
Sh3bgr	*t*(9) = -4.995; *p* = 0.00362	*t*(9) = -2.786; *p* = 0.0295

**Second environment**	**Ts66Yah**	**Ts65Dn**
**Y maze**		**Not done**
Sexe	Male	
Nb of litters	8	
number of individuals	13 2n/11 Ts	
Arms visited within genotype	*t*(22) = 1.141; *p* = 0.266	
% spontaneous alternation within genotype	*t*(22) = 4.436; *p* = 0.0002	
One sample t test spontaneous alternation vs 50% 2n	*t*(12) = 4.348; *p* = 0.0009	
One sample t test spontaneous alternationvs 50% Ts	*t*(10) = 1.814; *p* = 0.0997	
**Y maze**		**Not done**

Disease Models & Mechanisms • Supplementary information

Sexe	Female	
Nb of litters	8	
number of individuals	14 2n/12 Ts	
Arms visited within genotype	*t*(24)= 2.122; *p* = 0.044	
% spontaneous alternation within genotype	*t*(24) = 2.694; *p* = 0.0127	
One sample t test spontaneous alternation vs 50% 2n	*t*(13) = 3.235; *p* = 0.0065	
One sample t test spontaneous alternationvs 50% Ts	*t*(11) = 0.1530; *p* = 0.881	
ANOVA "nbr of entries" genotype/sexe for sexe	$F_{(1,46)}$ = 0.01; *p* = 0.921	
ANOVA "Spontaneous alternation" genotype/sexe for sexe	$F_{(1,46)}$ = 0.25; *p* = 0.619	
**NOR 24H**		**Not done**
Sexe	Male	
Nb of litters	8	
number of individuals	13 2n/11 Ts	
Object within genotype in habituation	*t*(22) = 2.091; *p* = 0.048	
One sample t test %NO vs 50% 2n	*t*(12) = 2.25; *p* = 0.044	
One sample t test %NO vs 50% Ts	*t*(10) = 2.017; *p* = 0.0713	
**NOR 24H**		**Not done**
Sexe	Female	
Nb of litters	8	
number of individuals	14 2n/12 Ts	
Object within genotype in habituation	*t*(24) = 3.676; *p* = 0.0012	
One sample t test %NO vs 50% 2n	*t*(13) = 4.088; *p* = 0.0013	
One sample t test %NO vs 50% Ts	*t*(11) = 0.9938; *p* = 0.341	
ANOVA "sniffing time presentation" genotype/sexe for sexe	$F_{(1,46)}$ = 0.411; *p* = 0.525	
ANOVA "RI" genotype/sexe for sexe	$F_{(1,46)}$ = 0.2009; *p* = 0.66	

Table S2. Phenotypic variables used for the Gdaphen analysis.

Variable	Detailed name	unit (SI)
OF:Total_distance	total distance travelled in the open field test	m
OF:Rears	total number of rears in the open field	
OF:Sterotypies	total number of stereotypies	
OF:V	Mean velocity during the open field session	cm/s
CA:Locomotion:Hab	nb of back and forth during the habituation period of the circadian activity	
CA:Rears:Hab	total number of rears during the circadian activity	
CA:Locomotion:Light	nb of back and forth during the light period of the circadian activity	
CA:Rears:Light	total number of rears during the light phase of the circadian activity	
CA:Locomotion:N1	nb of back and forth during the night phase 1 of the circadian activity	
CA:Rears:N1	total number of rears during the night phase 1 of the circadian activity	
CA:Locomotion:N2	nb of back and forth during the night phase 2 of the circadian activity	
CA:Rears:N2	total number of rears during the night phase 2 of the circadian activity	
YM:Arm_Entries	total number of arm entries in the Y maze	
YM:Spont_Alter	Percentage of spontaneous alternation in the Y maze	
NOR:Pres_HG	time sniffing the left object in the presentation of the NOR	s
NOR:Pres_HD	time sniffing the right object in the presentation of the NOR	s
NOR:Test_FO	time sniffing the familiar object in the test phase of the NOR	s
NOR:Test_NO	time sniffing the novel object in the test phase of the NOR	s
NOR:Test_NO	Percentage of time spent exploring the novel object in the NOR	
MWM:PT1_TQ	Percentage of time spent in the targeted quadrant of the probe test 1	
MWM:PTRev_TQ	Percentage of time spent in the targeted quadrant of the probe test for the reversal	
FC:Hab1	Freezing time during the habituation phase 1 of the fear conditioning	s
FC:Hab2	Freezing time during the habituation phase 2 of the fear conditioning	s
FC:Cont1	Freezing time during the contextual phase 1 of the fear conditioning	s
FC:Cont2	Freezing time during the contextual phase 2 of the fear conditioning	s
FC:Cont3	Freezing time during the contextual phase 3 of the fear conditioning	s
FC:Precue1	Freezing time during the precued phase 1 of the fear conditioning	
FC:Cue1	Freezing time during the cued phase 1 of the fear conditioning	
FC:Precue2	Freezing time during the precued phase 2 of the fear conditioning	
FC:Cue2	Freezing time during the cued phase 2 of the fear conditioning	
Sperm:C°	Sperm concentration	10^6/ml
Sperm:Motility	Percentage of motile spermatozoa	
Sperm:Rapid_cells	Percentage of rapid spermatozoa	
Sperm:Progressive	Percentage of progressive spermatozoa	
Nesting:Score	Standardized core of nesting activities	

Disease Models & Mechanisms • Supplementary information

Table S3. Highly correlated variables identified in the GDAPHEN analysis of Ts66Yah, Ts65Dn and the joint analysis of Ts65Dn and Ts66Yah behavioral data.

Models Analysed	Variable 1	Variable 2	Correlation coefficient
Ts66Yah vs wt	CA:Rears:N1	CA:Locomotion:N1	0.808
Ts66Yah vs wt	CA:Rears:Hab	CA:Locomotion:Hab	0.796
Ts66Yah vs wt	CA:Locomotion:Light	CA:Locomotion:N2	0.853
Ts66Yah vs wt	Sperm:Motility	Sperm:Rapid_cells	0.890
Ts66Yah vs wt	Sperm:Motility	Sperm:Progressive	0.779
Ts66Yah vs wt	Sperm:Rapid_cells	Sperm:Motility	0.890
Ts66Yah vs wt	Sperm:Rapid_cells	Sperm:Progressive	0.927
Ts66Yah vs wt	FC:Cue2	FC:Cue1	0.822
Ts66Yah vs wt	FC:Cue2	FC:Precue2	0.806
Ts66Yah vs wt	CA:Locomotion:Hab	CA:Rears:Hab	0.796
Ts66Yah vs wt	CA:Locomotion:Hab	CA:Locomotion:N1	0.770
Ts66Yah vs wt	CA:Rears:Light	CA:Rears:N2	0.967
Ts66Yah vs wt	OF:V	OF:Total_distance	1.000
Ts66Yah vs wt	FC:Cue1	FC:Precue2	0.811
Ts66Yah vs wt	FC:Cue1	FC:Cue2	0.822
Ts66Yah vs wt	FC:Hab1	FC:Hab2	0.801
Ts65Dn vs wt	CA:Rears:N1	CA:Rears:Hab	0.800
Ts65Dn vs wt	Sperm:Motility	Sperm:C°	0.820
Ts65Dn vs wt	Sperm:Motility	Sperm:Rapid_cells	0.981
Ts65Dn vs wt	Sperm:Motility	Sperm:Progressive	0.865
Ts65Dn vs wt	Sperm:Rapid_cells	Sperm:C°	0.775
Ts65Dn vs wt	Sperm:Rapid_cells	Sperm:Motility	0.981
Ts65Dn vs wt	Sperm:Rapid_cells	Sperm:Progressive	0.863
Ts65Dn vs wt	CA:Locomotion:N2	CA:Locomotion:Hab	0.782
Ts65Dn vs wt	CA:Locomotion:N2	CA:Locomotion:N1	0.782
Ts65Dn vs wt	CA:Locomotion:Light	CA:Rears:Light	0.925
Ts65Dn vs wt	OF:Total_distance	OF:V	1.000
Ts65Dn vs wt	CA:Locomotion:Hab	CA:Locomotion:N1	1.000
Ts65Dn vs wt	CA:Locomotion:Hab	CA:Locomotion:N2	0.782
Ts65Dn vs wt	FC:Cue2	FC::Cue1	0.767
Ts65Dn, Ts66Yah vs wt	Sperm:Rapid_cells	Sperm:Motility	0.987
Ts65Dn, Ts66Yah vs wt	Sperm:Rapid_cells	Sperm:Progressive	0.872
Ts65Dn, Ts66Yah vs wt	Sperm:Motility	Sperm:Rapid_cells	0.987
Ts65Dn, Ts66Yah vs wt	Sperm:Motility	Sperm:Progressive	0.849
Ts65Dn, Ts66Yah vs wt	CA:Rears:N1	CA:Locomotion:N1	0.763
Ts65Dn, Ts66Yah vs wt	CA:Locomotion:N2	CA:Locomotion:N1	0.777
Ts65Dn, Ts66Yah vs wt	CA:Rears:Light (nb)	CA:Rears:N2	0.864

Disease Models & Mechanisms • Supplementary information

Ts65Dn, Ts66Yah vs wt	CA:Locomotion:N1	CA:Locomotion:Hab	0.869
Ts65Dn, Ts66Yah vs wt	CA:Locomotion:N1	CA:Rears:N1	0.763
Ts65Dn, Ts66Yah vs wt	CA:Locomotion:N1	CA:Locomotion:N2	0.777
Ts65Dn, Ts66Yah vs wt	OF:Total_distance	OF:V	1.000
Ts65Dn, Ts66Yah vs wt	FC:Cue2	FC:Cue1	0.794

Table S4. Discriminating variables identified from the GLM-Net and RF analysis combining the Ts66Yah &Ts65Dn data.

	Ts65Dn		Ts66Yah	
Variable	2n	DS	2n	DS
GLMNET				
Sperm:Progressive	17.6	27.5	15.7	15.7
FC:Hab2	10.1	19.1	18.0	10.1
MWM:PTRev_TQ	5.2	22.4	8.4	5.2
NOR:Test_NO_%	12.1	25.9	12.3	12.1
NOR:Test_NO_T	0.3	19.1	12.5	0.3
YM:Spont_Alter	8.1	17.6	7.1	7.1
CA:Rears:N2	25.1	29.0	50.0	25.1
CA:Locomotion:Light	3.6	2.4	3.5	2.4
CA:Locomotion:Hab	0.0	0.0	11.2	23.7
OF:Sterotypies	22.1	21.7	21.7	100.0
OF:Rears	6.4	40.8	15.4	6.4
Random Forest				
Sperm:Progressive	30.3	58.2	39.6	18.0
FC:Hab2	50.0	13.9	13.4	19.5
MWM:PTRev_TQ	10.9	29.7	13.9	6.9
NOR:Test_NO_%	11.6	16.6	29.6	25.1
NOR:Test_NO_T	48.4	15.1	12.6	41.5
YM:Spont_Alter	34.0	31.1	15.5	21.9
CA:Rears:N2	27.9	72.4	25.3	13.6
CA:Locomotion:Light	24.4	14.0	8.5	21.0
CA:Locomotion:Hab	21.5	33.3	1.5	6.6
OF:Sterotypies	75.7	47.3	0.0	100.0
OF:Rears	18.5	5.3	15.4	5.1

Table S5. Comparison of the most discriminative variables identified between the genotypes for Ts66Yah vs wt, or for Ts65Dn vs wt, using the GLM-Net or RF methods.

Ts66Yah vs Wt

**GLM-Net**	**Sel model >30%: 9 Variables Accuracy: 0.941**	**RF**	**Sel model >30%: 9 Variables Accuracy: 0.925**
Overall	Variable	Overall	Variable
100	Sperm:C°	100	Sperm:C°
72.57	Sperm:Progressive	52.56	Sperm:Progressive
39.21	MWM:PTRev_TQ	30.58	Nesting:Score
33.28	Nesting:Score	29.14	MWM:PT1_TQ
25.62	CA:Locomotion:N1	22.07	OF:Total_distance
5.86	CA:Rears:N2	10.33	CA:Rears:N2
5.35	OF:Total_distance	8.66	FC:Cont3 (s)
1.94	FC:Cont3	3.64	CA:Locomotion:N1
0	MWM:PT1_TQ	0	MWM:: PTRev TQ

Ts65Dn vs wt

**GLM-Net**	**Sel model >30%: 11 Variables Accuracy: 0.7**	**RF**	**Sel model >30%: 11 Variables Accuracy: 0.891**
100	FC:Precue1	100	Sperm:C°
57.02	FC:Cont3	65.11	FC:Cont3
52.43	MWM:PT1_TQ	42.15	FC:Precue1
46.81	YM:Arm_Entries	36.11	Nesting:Score
30.66	Nesting:Score	29.68	CA:Rears:Hab
17.96	Sperm:C°	11.99	YM:Arm_Entries
16.82	NOR:Test_NO	7.39	NOR:Pres_HD
12.17	FC:Hab1	7.09	FC:Hab1
5.75	OF:Rears	3.13	NOR:Test_NO
5.75	NOR:Pres_HD	1.13	OF:Rears
0	CA:Rears:Hab	0	MWM:PT1_TQ (%)

Table S6. Landmarks used for craniofacial analysis of mouse models.

Cranium			Description
Right	Median	Left	
	1		Nasale
	2		Nasion
	3		Bregma
	4		Parietal-occipital junction
	5		Midline of the interparietal-occipital junction
	6		Dorsal midpoint of the foramen magnum
7		15	Dorsal-most point of the incisor alveoli
8		16	Parietal-premaxillar-maxillar junction
9		17	Anterior-most point of the zygomatic spine
10		18	Posterior-most point of the frontal-maxillary dorsal junction
11		19	Anterior-most point of the squamosal-parietal junction
12		20	Anterior-most point of the zygomatic-squamosal junction
13		21	Posterior-most point of the zygomatic-squamosal junction
14		22	Tip of the post-tympanic hook.
23		31	Anterior-most point of the anterior palatine foramen
24		32	Posterior-most point of the anterior palatine foramen
25		33	Ventral-most point of the premaxillar, maxillar and anterior palatine foramen junction
26		34	Mesial-most point of the first upper molar cervix
27		35	Point of greatest curvature of the posterior margin of malar process
28		36	Distal-most point of the third upper molar cervix
29		37	Point of greatest curvature of the squamosal retroversus process
30		38	Antero-medial projection of ectotympanic in basicranial
	39		ventral midpoint of the foramen magnum
Mandibule			
1		12	Tip of the coronoid process
2		13	Distal-most point of the third lower molar cervix
3		14	Mesial-most point of the first lower molar cervix
4		15	Dorsal-most point of the incisor alveoli
5		16	Inferior-most point of the incisor alveoli
6		17	Inferior-most point on border of ramus inferior to incisor alveolar
7		18	Superior-most point on inferior border of mandibular ramus
8		19	Tip of the mandibular angle
9		20	Ventral-most point of the mandibular condyle
10		21	Anterior-most point of the mandibular condyle
11		22	Mandibular foramen

Disease Models & Mechanisms • Supplementary information

Table S7. Summary of the differential expression analysis and differential functional analysis for each DS model dataset.

	Ts65Dn EC	Ts65Dn HC	Ts66Yah EC	Ts66Yah HC
Differentially Expressed Genes (DEGs) identified by FCROS	1691	1836	2220	1902
Genes from the Mmu17 centromeric genes				
Expressed genes	53	53	53	53
Upregulated Triplicated (TEGs)	41	37	7	10
Genes from the Mmu16 homologous region to Hsa21				
Expressed genes	94	94	94	94
Upregulated Triplicated (TEGs)	81	85	82	84
Number of GAGE KEGG and GOs (CC.BP.MF) terms dis-regulated in the trisomic models (FDR<0.1)	135	493	40	323
Number of GAGE KEGG and GOs upregulated in the trisomic models (FDR<0.1)	95	459	1	260

Table S8. Number of Differentially Expressed Genes (DEGs) found per chromosome and percentage of DEGs per chromosome after correcting by chromosome length.

Chr	Chr length (pb)	DEGs Ts66Yah HIP	DEGs Ts66Yah EC	DEGs Ts65Ds HIP	DEGs Ts65Dn EC	%DEGs Ts66Yah HIP /10^6bp	%DEGs Ts66Yah EC/10^6bp	%DEGs Ts65Dn HIP 10^6bp	%DEGs Ts65Dn EC/10^6bp
1	195154279	142	138	132	117	0.73	0.71	0.68	0.60
2	181755017	152	183	110	116	0.84	1.01	0.61	0.64
3	159745316	123	136	90	103	0.77	0.85	0.56	0.64
4	156860686	77	106	97	94	0.49	0.68	0.62	0.60
5	151758149	118	117	124	105	0.78	0.77	0.82	0.69
6	149588044	115	127	91	87	0.77	0.85	0.61	0.58
7	144995196	140	161	162	139	0.97	1.11	1.12	0.96
8	130127694	91	111	80	64	0.70	0.85	0.61	0.49
9	124359700	98	109	96	88	0.79	0.88	0.77	0.71
10	130530862	84	104	80	78	0.64	0.80	0.61	0.60
11	121973369	96	133	120	92	0.79	1.09	0.98	0.75
12	120092757	73	94	64	75	0.61	0.78	0.53	0.62
13	120883175	75	83	83	83	0.62	0.69	0.69	0.69
14	125139656	62	90	72	59	0.50	0.72	0.58	0.47
15	104073951	72	102	49	58	0.69	0.98	0.47	0.56
16	98008968	109	119	101	78	1.11	1.21	1.03	0.80
17	95294699	106	118	100	100	1.11	1.24	1.05	1.05
18	90720763	56	51	52	39	0.62	0.56	0.57	0.43
19	61420004	49	68	39	44	0.80	1.11	0.63	0.72
X	169476592	60	69	79	61	0.35	0.41	0.47	0.36
Y	91455967	2	1	2	1	0.02	0.01	0.02	0.01

Table S9. Expression of genes from the centromeric part of the Mmu17 and the telomeric part of Mmu16 in the Ts66Yah and Ts65Dn Hippocampi (HC) or Entorhinal cortex (EC) (in red with a FC >1.2).

Gene	Chr	Start	Feature Type MGI	MGI Gene/ Marker ID	Ts65Dn EC	Ts65Dn HC	Ts66Yah EC	Ts66Yah HC
Gm10232	17	3044014	pseudogene	MGI:3641637	1.23	1.11	0.86	0.83
Pisd-ps2	17	3076578	pseudogene	MGI:3612472	1.41	1.43	0.96	1.1
Scaf8	17	3114972	protein coding gene	MGI:1925212	1.35	1.4	1.02	1.03
Tiam2	17	3326573	protein coding gene	MGI:1344338	1.73	1.49	1.01	1.01
Gm7043	17	3461800	pseudogene	MGI:3647242	0.68	0.71	0.84	0.89
Tfb1m	17	3519256	protein coding gene	MGI:2146851	1.47	1.55	1.04	1.13
Cldn20	17	3532554	protein coding gene	MGI:3646757	1.55	1.45	0.82	1.39
1700102H20Rik	17	3557824	lncRNA gene	MGI:1915480	1.03	1.54	0.58	1.15
Arid1b	17	4994332	protein coding gene	MGI:1926129	1.42	1.48	1.02	1.01
Gm15599	17	5110766	pseudogene	MGI:3783046	0.82	0.78	1.63	0.71
Gm29050	17	5388763	lncRNA gene	MGI:5579756	2.49	1.01	1.82	0.64
Tmem242	17	5410870	protein coding gene	MGI:1917794	1.38	1.34	0.94	0.93
Zdhhc14	17	5492557	protein coding gene	MGI:2653229	1.71	1.63	0.94	0.98
Gm26595	17	5817472	pseudogene	MGI:5477089	0.91	1.01	1.00	1.04
Gm26622	17	5837968	pseudogene	MGI:5477116	1.31	0.86	0.85	1.33
Snx9	17	5841329	protein coding gene	MGI:1913866	1.47	1.39	0.99	0.94
Synj2	17	5941280	protein coding gene	MGI:1201671	1.48	1.62	1.12	1.18
Serac1	17	6042196	protein coding gene	MGI:2447813	1.6	1.58	1.00	1.01
Gtf2h5	17	6079786	protein coding gene	MGI:107227	1.52	1.54	0.96	1.01
Tulp4	17	6106437	protein coding gene	MGI:1916092	1.3	1.3	1.13	1.08
Gm15590	17	6131854	pseudogene	MGI:3831433	0.84	0.87	0.82	1.08
Tmem181a	17	6256860	protein coding gene	MGI:1924356	0.92	1.09	1.67	1.34
Dynlt1a	17	6306340	protein coding gene	MGI:3807506	0.65	0.78	1.39	1.47
Dynlt1b	17	6430112	protein coding gene	MGI:98643	1.54	1.29	0.88	0.86
Tmem181b-ps	17	6438524	pseudogene	MGI:3779544	1.42	1.28	0.71	0.73
Dynlt1c	17	6601671	protein coding gene	MGI:3807476	2.76	1.01	0.62	0.56
Tmem181c-ps	17	6613753	pseudogene	MGI:3780993	2.6	2.16	1.02	0.57
Dynlt1f	17	6646602	protein coding gene	MGI:3780996	1.62	1.38	1.15	1.05
Sytl3	17	6659093	protein coding gene	MGI:1933367	1.5	2.01	0.59	0.54
Ezr	17	6738041	protein coding gene	MGI:98931	1.31	1.44	0.9	0.96
Rsph3b	17	6904413	protein coding gene	MGI:3630308	1.26	1.15	0.99	1.00
Tagap1	17	6955011	protein coding gene	MGI:1919786	1.23	1.17	1.02	1.04
Rnaset2b	17	6970634	protein coding gene	MGI:3702087	0.89	1.12	1.04	1.71
Rps6ka2	17	7170115	protein coding gene	MGI:1342290	1.4	1.21	1.04	1.17
Fndc1	17	7738569	protein coding gene	MGI:1915905	1.7	1.35	1.14	0.88
E430024P14Rik	17	7881106	lncRNA gene	MGI:2445079	2.06	1.55	0.89	1.04

Tagap	17	7926000	protein coding gene	MGI:3615484	2.6	2.65	0.68	0.67
Rsph3a	17	7945614	protein coding gene	MGI:1914082	1.96	1.53	1.09	1.25
Rnaset2a	17	8115445	protein coding gene	MGI:1915445	1.54	1.74	0.74	0.95
Fgfr1op	17	8165501	protein coding gene	MGI:1922546	1.51	1.33	1.01	0.96
Ccr6	17	8236043	protein coding gene	MGI:1333797	9.69	9.74	0.58	0.5
Mpc1	17	8282904	protein coding gene	MGI:1915240	1.15	1.22	1.18	1.42
4930506C21Rik	17	8293366	lncRNA gene	MGI:1922310	1.75	1.51	1.11	1.00
Sft2d1	17	8311102	protein coding gene	MGI:1918689	1.34	1.31	0.96	0.88
Prr18	17	8337459	protein coding gene	MGI:2443403	1.51	1.56	1.2	0.97
T2	17	8355992	protein coding gene	MGI:104658	2.22	1.22	0.85	1,00
T	17	8434423	protein coding gene	MGI:98472	1.71	1.89	1.00	1.34
Pde10a	17	8525372	protein coding gene	MGI:1345143	1.69	1.48	0.91	0.98
Gm15425	17	8564258	pseudogene	MGI:3705642	0.79	0.84	1.05	1.00
Gm17087	17	8565852	protein coding gene	MGI:4937914	1.23	1.19	1.08	0.92
1700010I14Rik	17	8988333	protein coding gene	MGI:1914181	1.37	1.29	1.39	1.32
6530411M01Rik	17	9147719	lncRNA gene	MGI:1915041	0.98	1.27	1.35	1.28
Gm8492	17	9320395	pseudogene	MGI:3643249	1.03	0.97	1.05	1.05
Mrpl39	16	84717576	protein coding gene	MGI:1351620	1.41	1.44	1.49	1.50
Jam2	16	84774123	protein coding gene	MGI:1933820	1.43	1.49	1.63	1.56
Atp5j	16	84827866	protein coding gene	MGI:107777	1.42	1.56	1.57	1.45
Gabpa	16	84834925	protein coding gene	MGI:95610	1.38	1.22	1.42	1.48
App	16	84949685	protein coding gene	MGI:88059	1.44	1.57	1.49	1.53
Gm10791	16	84972214	lncRNA gene	MGI:3641949	1.13	1.48	1.38	1.39
Cyyr1	16	85421533	protein coding gene	MGI:2152187	1.24	1.42	1.56	1.53
Adamts1	16	85793827	protein coding gene	MGI:109249	1.30	1.46	1.81	1.56
Adamts5	16	85856173	protein coding gene	MGI:1346321	1.48	1.72	1.59	1.60
N6amt1	16	87354185	protein coding gene	MGI:1915018	1.38	1.26	1.15	1.11
Ltn1	16	87376651	protein coding gene	MGI:1926163	1.38	1.41	1.72	1.58
Rwdd2b	16	87433407	protein coding gene	MGI:1858215	1.57	1.48	1.62	1.59
Usp16	16	87454703	protein coding gene	MGI:1921362	1.50	1.36	1.51	1.50
Cct8	16	87483326	protein coding gene	MGI:107183	1.38	1.46	1.61	1.52
Rpl31-ps4	16	87549062	pseudogene	MGI:3649126	0.78	0.93	0.92	1.44
Map3k7cl	16	87553330	protein coding gene	MGI:2446584	1.20	1.78	1.29	0.97
Bach1	16	87698945	protein coding gene	MGI:894680	1.41	1.37	1.50	1.35
2810407A14Rik	16	87784075	lncRNA gene	MGI:1917461	1.46	1.47	1.98	2.04
Grik1	16	87895900	protein coding gene	MGI:95814	1.49	1.40	1.37	1.39
Cldn17	16	88505807	protein coding gene	MGI:2652030	1.00	2.05	1.00	1.63
Cldn8	16	88560828	protein coding gene	MGI:1859286	1.00	0.92	1.00	0.96
Tiam1	16	89787111	protein coding gene	MGI:103306	1.56	1.36	1.60	1.51
Sod1	16	90220754	protein coding gene	MGI:98351	1.62	1.74	1.43	1.39
Scaf4	16	90225680	protein coding gene	MGI:2146350	1.42	1.42	1.24	1.28

Disease Models & Mechanisms • Supplementary information

Hunk	16	90386013	protein coding gene	MGI:1347352	1.27	1.53	1.71	1.60
1110008E08Rik	16	90554164	lncRNA gene	MGI:1915750	0.94	1.21	1.03	2.88
Mis18a	16	90719312	protein coding gene	MGI:1913828	1.63	1.29	1.56	1.45
Mrap	16	90738207	protein coding gene	MGI:1924287	1.45	1.29	1.06	1.80
Urb1	16	90751527	protein coding gene	MGI:2146468	1.40	1.49	1.55	1.72
Eva1c	16	90826719	protein coding gene	MGI:1918217	1.50	1.75	1.35	1.74
Synj1	16	90936092	protein coding gene	MGI:1354961	1.54	1.59	1.64	1.62
Paxbp1	16	91014037	protein coding gene	MGI:1914617	1.44	1.42	1.40	1.59
4932438H23Rik	16	91053935	protein coding gene	MGI:1921637	1.26	1.41	1.63	1.72
H3f3a-ps2	16	91114189	pseudogene	MGI:1101758	1.00	0.85	0.87	0.60
Olig2	16	91225457	protein coding gene	MGI:1355331	1.47	1.42	1.24	0.94
Olig1	16	91269772	protein coding gene	MGI:1355334	1.47	1.43	1.41	0.97
Ifnar2	16	91372783	protein coding gene	MGI:1098243	1.46	1.55	1.54	1.56
Il10rb	16	91406164	protein coding gene	MGI:109380	1.34	1.53	1.42	1.58
Ifnar1	16	91485238	protein coding gene	MGI:107658	1.49	1.48	1.62	1.60
Ifngr2	16	91547072	protein coding gene	MGI:107654	1.69	1.65	1.65	1.60
Tmem50b	16	91574503	protein coding gene	MGI:1925225	1.45	1.58	1.55	1.46
Dnajc28	16	91614254	protein coding gene	MGI:2181053	1.33	1.31	1.48	1.57
Gart	16	91621186	protein coding gene	MGI:95654	1.48	1.59	1.48	1.50
Son	16	91647506	protein coding gene	MGI:98353	1.21	1.30	1.30	1.52
Donson	16	91676808	protein coding gene	MGI:1890621	1.53	1.36	1.55	1.55
Atp5o	16	91684398	protein coding gene	MGI:106341	1.43	1.46	1.61	1.62
Gm10785	16	91688898	lncRNA gene	MGI:3642149	1.32	1.36	1.87	1.35
Cryzl1	16	91689322	protein coding gene	MGI:1913859	1.36	1.42	1.55	1.45
Itsn1	16	91729281	protein coding gene	MGI:1338069	1.52	1.49	1.46	1.41
Mrps6	16	92058270	protein coding gene	MGI:2153111	1.73	1.60	1.44	1.47
Slc5a3	16	92058322	protein coding gene	MGI:1858226	1.22	1.15	1.51	1.52
Kcne2	16	92292389	protein coding gene	MGI:1891123	0.99	1.99	0.98	1.55
Smim11	16	92301286	protein coding gene	MGI:1916186	1.40	1.26	1.47	1.51
Kcne1	16	92345982	protein coding gene	MGI:96673	1.15	2.93	1.30	1.33
Rcan1	16	92391953	protein coding gene	MGI:1890564	1.55	1.56	1.47	1.58
2410124H12Rik	16	92478742	lncRNA gene	MGI:1924035	1.16	1.11	0.93	1.94
Clic6	16	92485736	protein coding gene	MGI:2146607	1.73	1.87	1.31	1.49
Runx1	16	92601466	protein coding gene	MGI:99852	1.16	1.08	0.99	1.08
Setd4	16	93583457	protein coding gene	MGI:2136890	1.53	1.41	1.52	1.45
Cbr1	16	93605853	protein coding gene	MGI:88284	1.51	1.47	1.59	1.32
Cbr3	16	93683215	protein coding gene	MGI:1309992	1.51	1.57	1.39	1.42
Morc3	16	93832121	protein coding gene	MGI:2136841	1.33	1.33	1.53	1.56
Chaf1b	16	93883901	protein coding gene	MGI:1314881	1.22	1.98	1.67	1.48
Cldn14	16	93919031	protein coding gene	MGI:1860425	1.90	1.39	1.61	1.51
Sim2	16	94084931	protein coding gene	MGI:98307	1.20	1.56	1.90	1.71

Hlcs	16	94128882	protein coding gene	MGI:894646	1.59	1.40	1.44	1.43
Ripply3	16	94328420	protein coding gene	MGI:2181192	2.01	1.57	1.43	2.00
Pigp	16	94358763	protein coding gene	MGI:1860433	1.45	1.45	1.45	1.41
Ttc3	16	94370618	protein coding gene	MGI:1276539	1.49	1.50	1.51	1.53
Gm23692	16	94429073	snRNA gene	MGI:5453469	1.00	1.00	1.14	1.00
Dyrk1a	16	94570010	protein coding gene	MGI:1330299	1.36	1.41	1.53	1.45
Kcnj6	16	94748636	protein coding gene	MGI:104781	1.54	1.50	1.55	1.71
Kcnj15	16	95257558	protein coding gene	MGI:1310000	1.69	3.34	1.90	1.80
Erg	16	95359169	protein coding gene	MGI:95415	1.06	1.19	0.99	1.11
Ets2	16	95702075	protein coding gene	MGI:95456	1.38	1.38	1.37	1.43
Psmg1	16	95979933	protein coding gene	MGI:1860263	1.51	1.56	1.40	1.31
Brwd1	16	95992092	protein coding gene	MGI:1890651	1.43	1.43	1.50	1.47
Hmgn1	16	96120618	protein coding gene	MGI:96120	1.40	1.40	1.51	1.49
Wrb	16	96145407	protein coding gene	MGI:2136882	1.53	1.55	1.58	1.57
Lca5l	16	96158407	protein coding gene	MGI:3041157	1.60	1.41	1.59	1.62
Sh3bgr	16	96200450	protein coding gene	MGI:1354740	2.87	1.29	1.34	1.68
B3galt5	16	96235801	protein coding gene	MGI:2136878	1.31	1.33	1.54	1.49
Igsf5	16	96361668	protein coding gene	MGI:1919308	1.42	1.36	2.12	2.05
Itgb2l	16	96422288	protein coding gene	MGI:1277979	1.63	1.34	0.94	1.12
Pcp4	16	96467606	protein coding gene	MGI:97509	1.57	1.12	1.15	1.18
Dscam	16	96590840	protein coding gene	MGI:1196281	1.50	1.50	1.63	1.53
Bace2	16	97356742	protein coding gene	MGI:1860440	1.45	1.66	1.61	1.76
Mx1	16	97447035	polymorphic pseudogene	MGI:97243	1.55	1.50	2.22	1.89
Mx2	16	97535308	polymorphic pseudogene	MGI:97244	1.54	1.55	1.95	1.59
Tmprss2	16	97564682	protein coding gene	MGI:1354381	1.19	0.74	1.42	2.29
Ripk4	16	97741933	protein coding gene	MGI:1919638	3.05	2.38	2.44	2.01
Prdm15	16	97791467	protein coding gene	MGI:1930121	1.24	1.22	1.42	1.36
C2cd2	16	97855209	protein coding gene	MGI:1891883	1.37	1.44	1.72	1.61
Zbtb21	16	97943357	protein coding gene	MGI:1927240	1.22	1.30	1.45	1.42

Disease Models & Mechanisms • Supplementary information

Table S10. Summary of the functional pathways found in the models, the number of pathways grouped on each meta-pathway and the percentage of pathways inside each meta-pathway considering the total number of pathways.

	Ts66Yah HIP		Ts66Yah EC		Ts65Dn HIP		Ts65Dn EC	
Group of pathways	pathways/ group	% pathways per group	nb total pathways per group	% pathways per group	nb total pathways per group	% pathways per group	nb total pathways per group	% pathways per group
Synaptic	12	3,72	4	10	8	1,51	10	6,62
GABA system	1	0,31			1	0,19	5	3,31
Myelin sheat:SNARE complex					2	0,38		
Behaviour	3	0,93			2	0,38	1	0,66
Synaptic: Other pathways	12	3,72	4	10	4	0,75	10	6,62
Transcription & epigenomic regulation	22	6,81	2	5	24	4,53	10	6,62
Enzymes activity	52	16,1			88	16,6	40	26,49
Mitochondria	1	0,31					5	3,31
Ribosome	3	0,93			6	1,13	4	2,65
Compounds binding	16	4,95	3	7,5	28	5,28	19	12,58
Cell Structure & organelles	12	3,72	7	17,5	82	15,47	40	26,49
Phospho-Kinase	8	2,48			8	1,51	3	1,99
Hormone regulation	4	1,24						
Post-translation modification	1	0,31			4	0,75		
Replication	4	1,24	1	2,5	7	1,32		
Morphogenesis & development	32	9,91	14	35	89	16,79	9	5,96
Host response & defense	127	39,32	2	5	174	32,83	18	11,92
Metabolism	67	20,74	9	22,5	118	22,26	33	21,85
Degradation system	1	0,31			7	1,32	7	4,64
Cardiovascular & homeostasis	16	4,95			19	3,58	4	2,65
Apoptosis & cell death	2	0,62			9	1,7		
Connective-tissue cells			2	5	11	2,08	1	0,66
Fibers and cytoskeleton	5	1,55			5	0,94	18	11,92
Stimulus detection	2	0,62			4	0,75		
Signaling pathways	14	4,33	2	5	30	5,66	6	3,97

Disease Models & Mechanisms • Supplementary information

Channels and location signals	55	17,03			81	15,28	14	9,27
Sexual development & embryogenesis	2	0,62					3	1,99
Aging			1	2,5				

REFERENCES

Avants, B. B., C. L. Epstein, M. Grossman and J. C. Gee, 2008 Symmetric diffeomorphic image registration with cross-correlation: Evaluating automated labeling of elderly and neurodegenerative brain. Medical Image Analysis 12**:** 26-41.

Birling, M. C., L. Schaeffer, P. Andre, L. Lindner, D. Marechal *et al.*, 2017 Efficient and rapid generation of large genomic variants in rats and mice using CRISMERE. Sci Rep 7**:** 43331.

Duchon, A., M. Del Mar Muniz Moreno, S. Martin Lorenzo, M. P. Silva de Souza, C. Chevalier *et al.*, 2021 Multi-influential genetic interactions alter behaviour and cognition through six main biological cascades in Down syndrome mouse models. Hum Mol Genet 30**:** 771-788.

Duchon, A., M. Raveau, C. Chevalier, V. Nalesso, A. J. Sharp *et al.*, 2011 Identification of the translocation breakpoints in the Ts65Dn and Ts1Cje mouse lines: relevance for modeling down syndrome. Mammalian Genome 22**:** 674-684.

Ma, Y., P. R. Hof, S. C. Grant, S. J. Blackband, R. Bennett *et al.*, 2005 A three-dimensional digital atlas database of the adult C57BL/6J mouse brain by magnetic resonance microscopy. Neuroscience 135**:** 1203-1215.

Sawiak, S. J., N. I. Wood, G. B. Williams, A. J. Morton and T. A. Carpenter, 2009 SPMMouse: A new toolbox for SPM in animal brain. Proc. Int'l. Soc. Mag. Res. Med.**:** 1086.

Szklarczyk, D., J. H. Morris, H. Cook, M. Kuhn, S. Wyder *et al.*, 2017 The STRING database in 2017: quality-controlled protein-protein association networks, made broadly accessible. Nucleic Acids Res 45**:** D362-D368.

Tang, H., F. Zhong, W. Liu, F. He and H. Xie, 2015 PathPPI: an integrated dataset of human pathways and protein-protein interactions. Sci China Life Sci 58**:** 579-589.

Tustison, N. J., B. B. Avants, P. A. Cook, Y. J. Zheng, A. Egan *et al.*, 2010 N4ITK: Improved N3 Bias Correction. Ieee Transactions on Medical Imaging 29**:** 1310-1320.

Warfield, S. K., K. H. Zou and W. M. Wells, 2004 Simultaneous truth and performance level estimation (STAPLE): An algorithm for the validation of image segmentation. Ieee Transactions on Medical Imaging 23**:** 903-921.
